# An effective one-pot access to polynuclear dispiroheterocyclic structures comprising pyrrolidinyloxindole and imidazothiazolotriazine moieties via a 1,3-dipolar cycloaddition strategy

**DOI:** 10.3762/bjoc.12.216

**Published:** 2016-10-24

**Authors:** Alexei N Izmest’ev, Galina A Gazieva, Natalya V Sigay, Sergei A Serkov, Valentina A Karnoukhova, Vadim V Kachala, Alexander S Shashkov, Igor E Zanin, Angelina N Kravchenko, Nina N Makhova

**Affiliations:** 1 N. D. Zelinsky Institute of Organic Chemistry, Russian Academy of Sciences, Leninsky Prosp., 47, Moscow 119991, Russian Federation; 2 A. N. Nesmeyanov Institute of Organoelement Compounds, Russian Academy of Sciences, Vavilova Str., 28, Moscow 119991, Russian Federation; 3 Voronezh State University, Universitetskaya Pl., 1, Voronezh 394000, Russian Federation

**Keywords:** azomethine ylides, cycloaddition, diastereoselectivity, nitrogen heterocycles, spiro compounds

## Abstract

An effective and highly regio- and diastereoselective one-pot method for the synthesis of new polynuclear dispiroheterocyclic systems with five stereogenic centers (dispiro[imidazo[4,5-*e*]thiazolo[3,2-*b*]-1,2,4-triazine-6,3′-pyrrolidine-2′,3′′-indoles]) comprising pyrrolidinyloxindole and imidazo[4,5-*e*]thiazolo[3,2-*b*]-1,2,4-triazine moieties has been developed. The method relies on a 1,3-dipolar cycloaddition of azomethine ylides generated in situ from isatin derivatives and sarcosine to 6-benzylideneimidazo[4,5-*e*]thiazolo[3,2-*b*]-1,2,4-triazine-2,7-diones.

## Introduction

**Figure 1:**
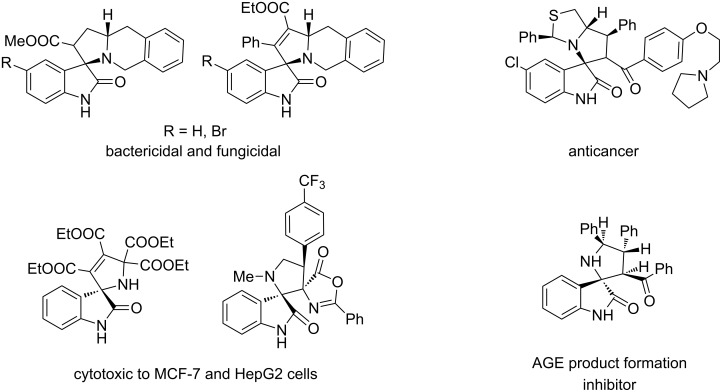
Bioactive 2,3’-spiropyrrolidinyloxindoles.

A global trend in modern organic chemistry is the design of molecular systems with various degrees of complexity to maximize the incorporation of useful properties while optimizing cost and efficiency [1]. A very extended and powerful approach for constructing complex N-heterocyclic systems in a regio and stereocontrolled fashion is the 1,3-dipolar cycloaddition of azomethine ylides to electron-deficient alkenes as dipolarophiles [2–8]. The in situ preparation of azomethine ylides from different carbonyl and amino components makes the cycloaddition one of the most valuable means of combinatorial chemistry. Such multicomponent reactions are characterized by productivity, operational simplicity, and efficiency [9–13]. A highly interesting class of heterocycles which is accessible through 1,3-dipolar cycloaddition reactions are compounds having the spiropyrrolidinyloxindole core [14–18]. The 3,3’-spiropyrrolidinyloxindole unit is found in the molecular skeleton of a large family of natural alkaloids with remarkable bioactivity profiles and interesting structural properties [19–20]. Derivatives with the 2,3’-spiropyrrolidinyloxindole core show various biological effects such as bactericidal and fungicidal [21], anticancer [22], cytotoxic to MCF-7 and HepG2 cells [23–24], and advanced glycation end (AGE) product formation inhibitory activities [25] (Figure 1).

Over the last decade, a lot of publications have been devoted to the synthesis of dispiro compounds comprising pyrrolidine, oxindole, and other heterocycle moieties and to the evaluation of their physiological properties [24,26–32]. In this regard, our attention was directed towards hetero-annelated 1,2,4-triazines, because this motif is part of many natural and synthetic bioactive products [33–40]. The pyrimido[5,4-*e*]-1,2,4-triazine constitutes the core of the antibiotics fervenulin, xanthothricin, and reumycin [33–34]. Other hetero-annelated 1,2,4-triazines reveal antiviral effect against influenza A and B viruses [35], anti-HIV and anticancer [36–37], antimicrobial and antifungal activities as well as cytotoxicity to MCF-7 cells [38–39]. Based on these observations we therefore aimed at combining the spiropyrrolidinyloxindole motif with hetero-annelated 1,2,4-triazine scaffolds.

**Scheme 1:**
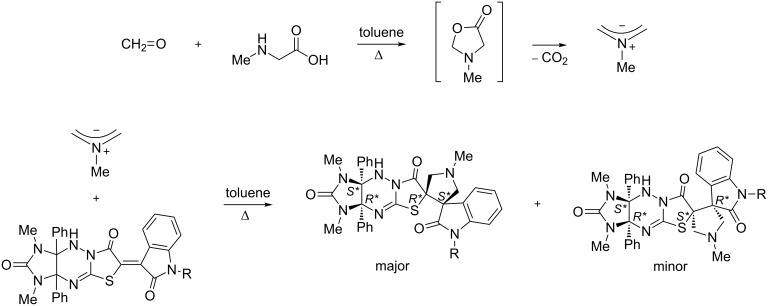
Earlier studied cycloaddition reaction.

Recently, we have already combined the imidazothiazolotriazine and 3,3’-spiropyrrolidinyloxindole moieties by a 1,3-dipolar cycloaddition of an azomethine ylide generated in situ from paraformaldehyde and sarcosine to oxoindolylidene derivatives of imidazothiazolotriazine. During this work we have found that the “small” azomethine ylide generated from paraformaldehyde and sarcosine approaches the double bond plane in (oxoindolylidene)imidazothiazolotriazines mainly from the side of the imidazolidine ring opposite to the phenyl groups (syn attack) (Scheme 1) [5].

To further expand the spectrum of biological activity, it is of interest to synthesize the other types of spiro compounds such as the 2,3’-spiropyrrolidinyloxindole structure which is isosteric with the 3,3’-spiropyrrolidinyloxindole and to study the diastereoselectivity of its formation. It could be expected that the cycloaddition of more bulky azomethine ylides generated from isatins and sarcosine to benzylidene derivatives of the same imidazothiazolotriazine will proceed from the less sterically hindered side [41] (anti attack).

Herein, we report a regio- and diastereoselective one-pot method for the synthesis of a novel class of polynuclear dispiroheterocyclic structures comprising 2,3’-spiropyrrolidinyloxindole and imidazo[4,5-*e*]thiazolo[3,2-*b*]-1,2,4-triazine moieties. The synthesis is based on a 1,3-dipolar cycloaddition of azomethine ylides generated in situ from isatins and sarcosine to tailor-made 6-benzylideneimidazo[4,5-*e*]thiazolo[3,2-*b*]-1,2,4-triazine-2,7-diones.

## Results and Discussion

**Scheme 2:**
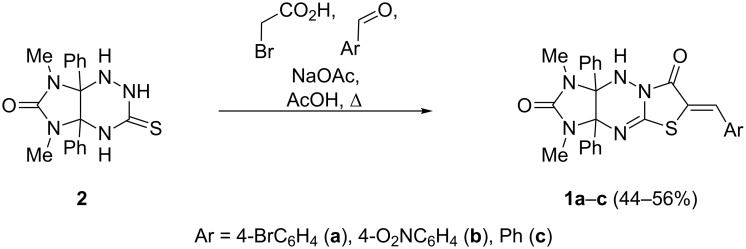
Synthesis of dipolarophiles **1a**–**c**.

The required dipolarophiles **1a–c** were prepared by the three-component condensation of imidazotriazinethione **2**, bromoacetic acid, and aromatic aldehydes (Scheme 2), as was described earlier by us [41]. The starting compound **2** is readily accessible and can be synthesized from 4,5-dihydroxy-4,5-diphenylimidazolidine-2-one [42] and thiosemicarbazide in 96% yield [43].

To optimize the 1,3-dipolar cycloaddition reaction conditions, 4-bromobenzylidene derivative **1a** was chosen as a model substrate in the reaction with sarcosine and isatin **3a**. The solvent, reaction time, and temperature were varied (Table 1).

**Table 1:** Optimization of the 1,3-dipolar cycloaddition reaction conditions.^a^

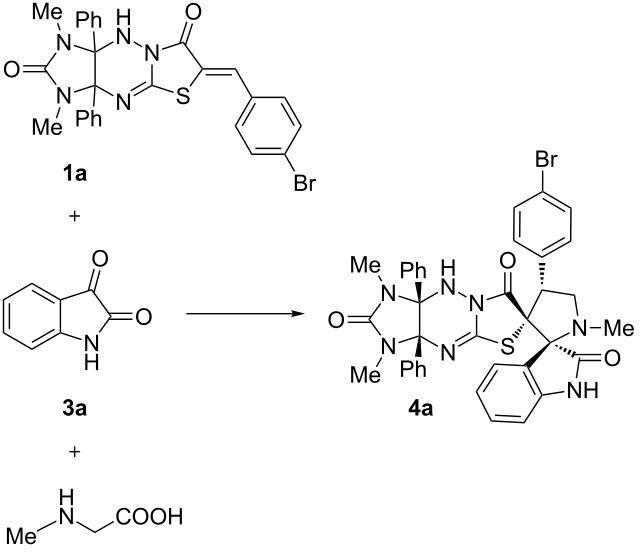				

Entry	Solvent	Temp. (°C)	Time (h)	Yield (%)^b^

1	EtOH	reflux	24	20
2	EtOH	reflux	42	23
3	toluene	reflux	24	15
4	CHCl_3_	reflux	24	50
5	CHCl_3_	reflux	42	59
6	MeCN	reflux	24	56
7	MeCN	reflux	30	71
8	MeCN	reflux	36	78
9	MeCN	reflux	42	79
10	MeCN	rt	42	0

^a^Reaction conditions: heating the mixture of compound **1a** (0.5 mmol), isatin (**3a**, 0.5 mmol), and sarcosine (0.5 mmol) in the corresponding solvent (40 mL) for the indicated time. ^b^Isolated yield.

As can be seen from Table 1, the cycloaddition was carried out in different solvents such as ethanol, chloroform, acetonitrile, and toluene. When the reaction was performed in ethanol or toluene, product **4a** was obtained in only low yields (Table 1, entries 1–3). Slightly increased yields were achieved in refluxing chloroform (Table 1, entries 4 and 5) and the best results were obtained in refluxing acetonitrile (Table 1, entries 6–9). At room temperature, the reaction does not proceed at all (Table 1, entry 10). Changing the reaction time from 24 h to 30 h significantly improved the yield of product **4a** whereas a further increase of the reaction time (>36 h) did not further improve the yield (Table 1, entries 6–9). The reaction progress was monitored by recording ^1^H NMR spectra of samples taken from the reaction mixture after 24, 30, 36, and 42 h. The formation of the pyrrolidine ring was detected by the appearance of triplets for the ring CH_2_ and CH group protons at 3.56, 3.95, and 4.45 ppm. Doublets for the C-3a and C-9a phenyl *ortho*-protons in compound **4a** (6.13 and 6.55 ppm, respectively) were observed at higher field than those of compound **1a** (6.75 and 6.83 ppm). The integrated intensity ratio of these protons was used to calculate the ratio of the target and starting compounds.

**Scheme 3:**
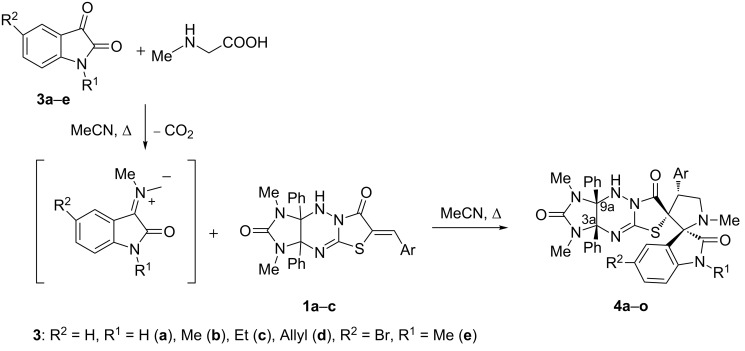
Synthesis of dispirocompounds **4a–o**.

**Figure 2:**
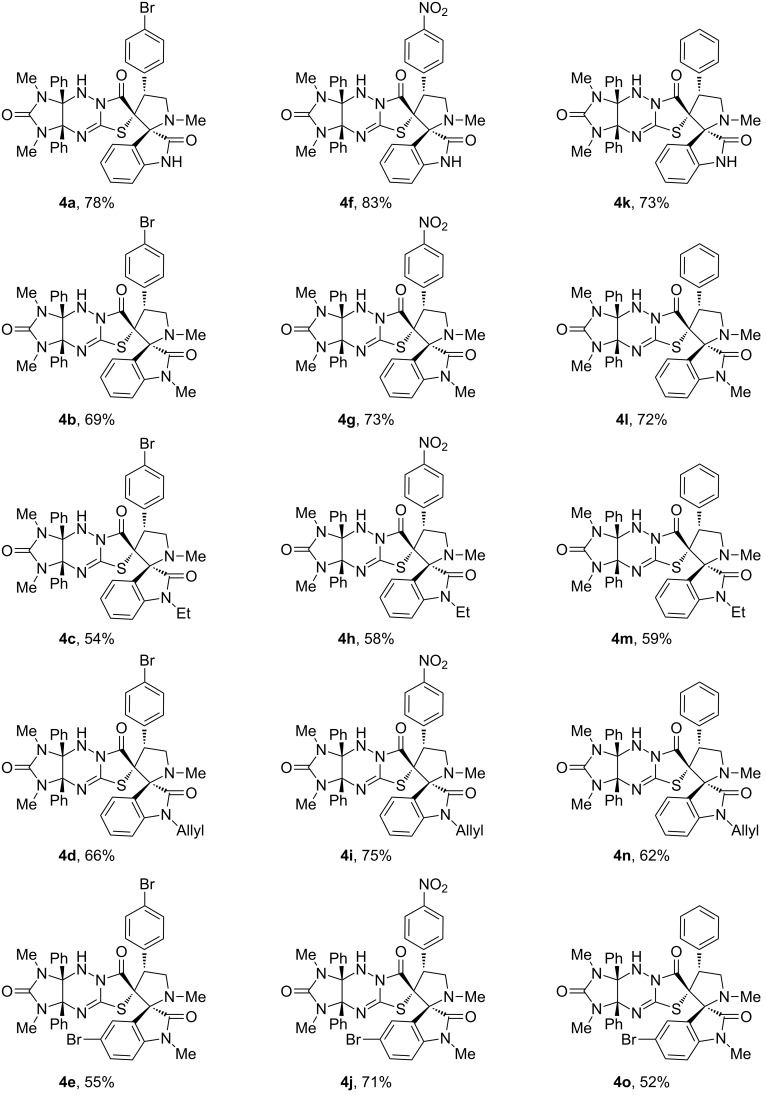
Synthesis of dispiro compounds **4a**–**o**. Reaction conditions: heating the mixture of compounds **1** (0.5 mmol), isatins **3** (0.5 mmol), and sarcosine (0.5 mmol) in acetonitrile (40 mL) for 36 h.

With the optimized conditions in hand, we next investigated the substrate scope for the reaction. First, various isatins **3a**–**e** were used for the generation of the azomethine ylides for the cycloaddition with compound **1a** (Scheme 3, Figure 2).

It was found that apart from model substrate **3a**, *N*-alkyl- (**3b**,**c**), *N*-allyl- (**3d**) and *N*-methyl-5-bromoisatins (**3e**) reacted with sarcosine and dipolarophile **1a** to afford the desired products **4a–e** in 54–78% yields. Next, the nitrobenzylidene and benzylidene derivatives **1b**,**c** were subjected to the reaction with isatins **3a–e** and sarcosine under the optimized conditions to afford the dispiro compounds **4f–o** in 52–83% yields. As shown in Figure 2, the best yields of the cycloadducts **4** were observed in the reaction of unsubstituted isatin (**3a**) as the carbonyl component for the generation of the azomethine ylide as well as for nitrobenzylidene derivative **1b** as dipolarophile.

**Scheme 4:**
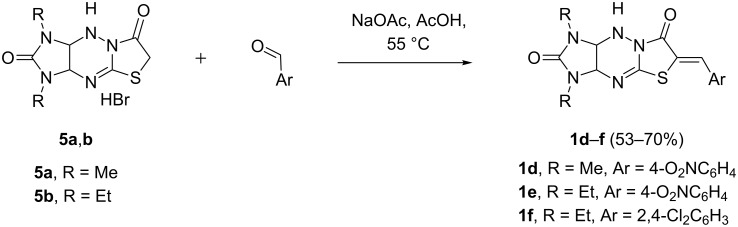
Synthesis of dipolarophiles **1d–f**.

To further extend the substrate scope of this reaction, we used benzylidene derivatives of other imidazothiazolotriazines **1d–f** without substituents at the bridge carbon atoms C(3a) and C(9a). The previously unknown compounds **1d–f** were synthesized in good yields by the condensation of imidazothiazolotriazines **5a**,**b** [44] with the corresponding aromatic aldehydes (Scheme 4).

These derivatives cannot be prepared by a three-component condensation of imidazotriazinethione with bromoacetic acid and an aromatic aldehyde, similarly to the synthesis of compounds **1a–c**. The reaction of aromatic aldehydes with imidazotriazinethiones without phenyl substituents in acidic media results in hydrazone formation and triazine-ring contraction [45].

**Figure 3:**
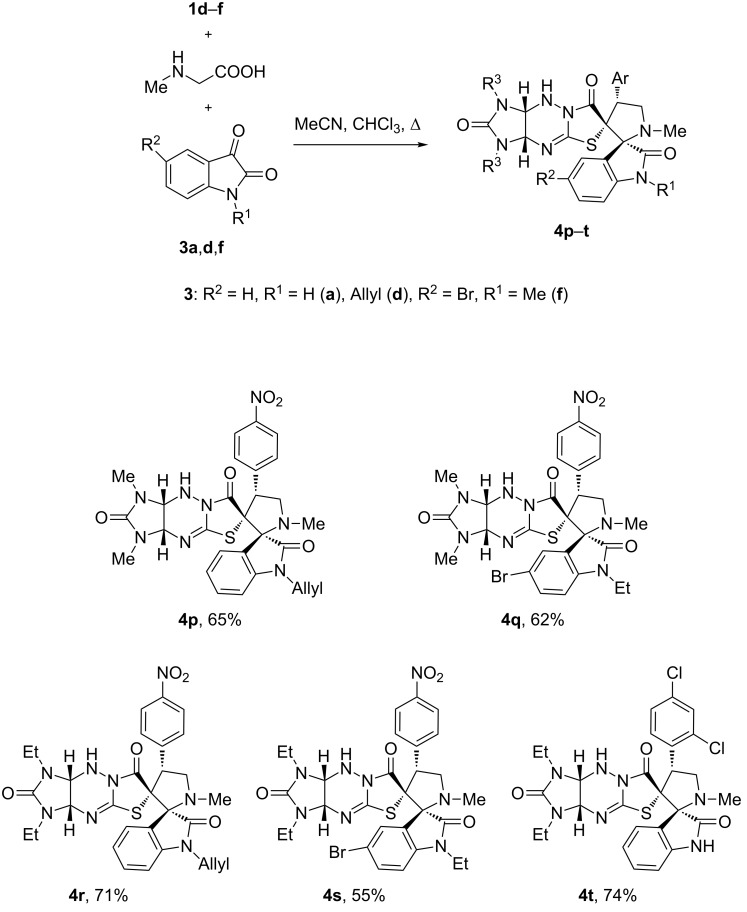
Synthesis of dispiro compounds **4p–t**. Reaction conditions: heating the solution of compounds **1** (0.5 mmol), isatins **3** (0.5 mmol), and sarcosine (0.5 mmol) in the mixture of acetonitrile (30 mL) and chloroform (10 mL) for 72 h.

The reaction of compounds **1d**–**f** with sarcosine and isatins **3a**,**d**,**f** also proceeded successfully, but required the addition of chloroform to the reaction mixture and an increased reaction time of 72 h. The novel dispiro compounds **4p–t** were finally obtained in 55–74% yields (Figure 3).

The structures of the synthesized compounds as well as the regioselectivity and diastereoselectivity of the cycloaddition were elucidated by spectroscopic methods and single crystal X-ray diffraction. The cycloadducts **4a–t** were characterized by IR, NMR, and HRMS analytical methods. The IR spectra of compounds **4** showed three intense absorption bands at 1728–1697, 1709–1680 (in some cases instead of these two bands, one broad band is observed), and 1649–1634 cm^−1^ that are characteristic of oxindole, imidazolidinone, and thiazolidinone ring carbonyl groups. The ^1^H NMR spectra of compounds **4** exhibited, along with the proton signals of the imidazothiazolotriazine and oxindole moieties, the signals for pyrrolidine ring protons: a singlet at 2.05–2.18 ppm for N(1’)Me group protons, two triplets at 3.49–3.66 and 3.93–4.05 ppm assignable to the protons of methylene C(5’)H_2_ group, and one triplet at 4.45–4.70 ppm corresponding to the proton of C(4’)H. This clearly demonstrates the regiochemistry of the cycloaddition. If the other possible regioisomer had formed, the ^1^H NMR spectra would have shown a singlet for the C(4’)H proton.

**Figure 4:**
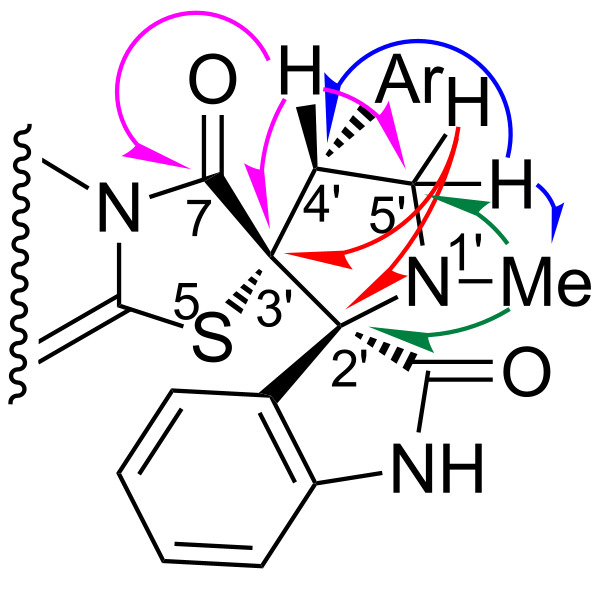
Key interactions in {^1^H-^13^C}HMBC spectrum of **4f**.

In more detail, the structure of compounds **4** was studied on the example of **4f** by COSY, {^1^H-^13^C}HSQC, {^1^H-^13^C}- and {^1^H-^15^N}HMBC NMR experiments. For instance, in the {^1^H-^13^C}HMBC spectrum of **4f**, the N(1’)Me protons (2.17 ppm) correlate with the C-5’ (57.52 ppm) and spiro C-2’ (80.29 ppm) carbons; the proton of C(4’)H (4.63 ppm) of the pyrrolidine ring correlates with the spiro carbon C-3’ (68.47 ppm) and the carbon atoms of the C(5’)H_2_ and C(7)=O (167.31 ppm) groups (Figure 4). The correlations of the C(5’)H_2_ group protons are different. One of them (4.02 ppm) shows cross-peaks with a neighboring C-4’ carbon atom (50.90 ppm) and the carbon atom of the N(1’)Me (35.38 ppm) group. Another one (3.61 ppm), correlates with both spiro carbon atoms C-2’ and C-3’ (see Supporting Information File 1 for full experimental data).

**Figure 5:**
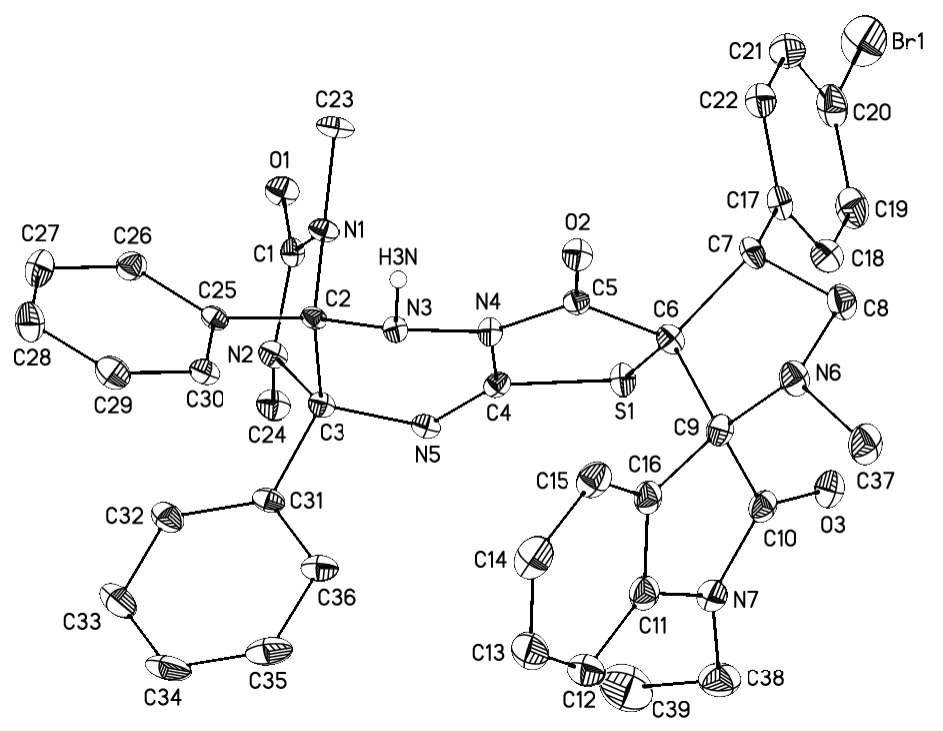
General view of **4c** in the crystal in thermal ellipsoids representation (50% probability). Hydrogen atoms connected to carbon atoms are omitted for clarity.

**Figure 6:**
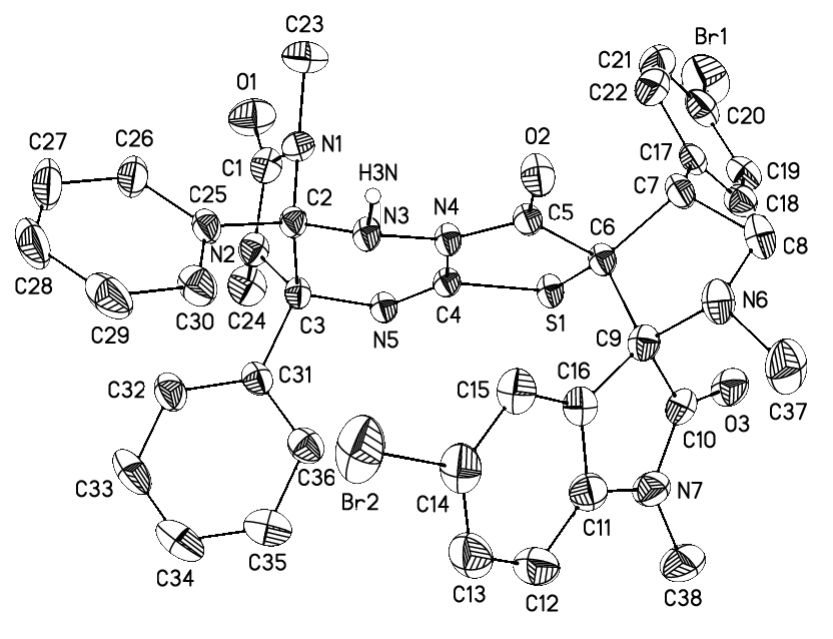
General view of **4e** in the crystal in thermal ellipsoids representation (40% probability). Hydrogen atoms connected to carbon atoms are omitted for clarity.

**Figure 7:**
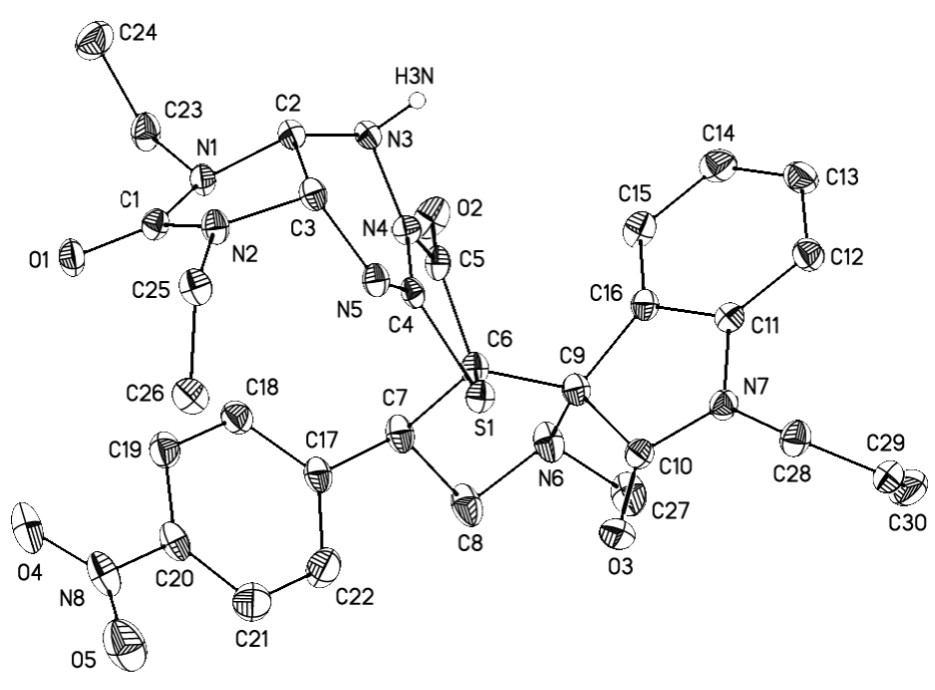
General view of **4r** in the crystal in thermal ellipsoids representation (50% probability). Hydrogen atoms connected to carbon atoms are omitted for clarity.

Finally, the regio- and stereochemistry of the cycloaddition were confirmed by single crystal X-ray diffraction analysis of compounds **4c** (Figure 5), **4e** (Figure 6), and **4r** (Figure 7) (see Supporting Information Files 2–4). The relative configurations of the stereocenters of compounds **4** are 2’R*, 3aS*, 3’R*, 4’R*, 9aR*.

The homogeneity of compounds **4b–d**,**f–i**,**l–n** was additionally confirmed by powder X-ray diffraction. The analysis of the experimental powder diffraction patterns of compounds **4b–d**,**f–i**,**l–n** show that the investigated samples were single phase (see Supporting Information File 1). Thus, the cycloaddition of azomethine ylides was found to be highly regioselective, as the electron-rich carbon of the dipole adds to the β-carbon of the α,β-unsaturated moiety of dipolarophile **1**. Further the reaction is diastereoselective, as only one diastereomer is obtained in good to high yields, although multiple (five) stereocenters are present in products **4**.

**Figure 8:**
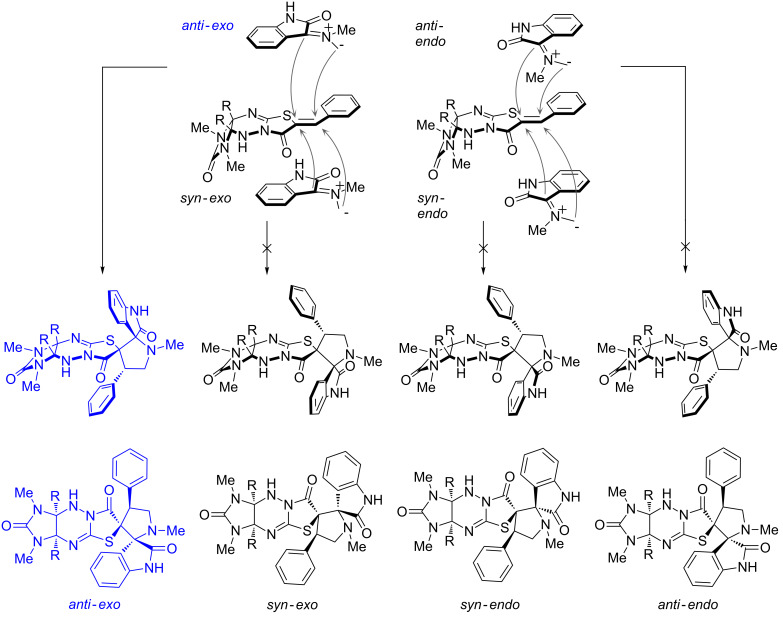
Modes of approach of azomethine ylide (R = H, Ph).

The possible approaches of the azomethine ylide are shown in Figure 8. The X-ray diffraction structures of **4c**, **4e**, and **4r** reflect that the cycloaddition proceeds via an *exo*-transition state, because the corresponding *endo*-transition state would require more energy of activation, as it would result in an electrostatic repulsion between the cis carbonyls thus increasing the free energy of activation [46–47]. As expected, the azomethine ylide adds at the double bond of **1a–c** from that side in which the phenyl substituents are directed (*anti*-*exo*).

## Conclusion

In summary, a simple, general, and efficient one-pot method for the construction of previously unknown substituted dispiro[imidazo[4,5-*e*]thiazolo[3,2-*b*]-1,2,4-triazine-6,3′-pyrrolidine-2′,3′′-indoles] was developed. This method is based on the highly regio- and diastereoselective 1,3-dipolar cycloaddition reaction of azomethine ylides generated in situ from various isatin derivatives and sarcosine to readily available 6-benzylideneimidazo[4,5-*e*]thiazolo[3,2-*b*]-1,2,4-triazine-2,7-diones. The synthesized structures represent a new class of promising bioactive polynuclear dispiroheterocyclic structures comprising pyrrolidinyloxindole and imidazothiazolotriazine moieties. Investigations of cytotoxic activities of the synthesized products against A549, HCT116, RD, and MCF7 cell lines are in progress.

## Supporting Information

File 1 Experimental and analytical data.

File 2 CIF file for compound **4c**.

File 3 CIF file for compound **4e**.

File 4 CIF file for compound **4r**.
