# Supplementary material for: An effective one-pot access to polynuclear dispiroheterocyclic structures comprising pyrrolidinyloxindole and imidazothiazolotriazine moieties via a 1,3-dipolar cycloaddition strategy
Source: Beilstein J Org Chem. 2016 Oct 24;12:2240–9. doi: 10.3762/bjoc.12.216 (PMC5238633; doi:10.3762/bjoc.12.216)
Supplement: File 1 — Experimental and analytical data. [file Beilstein_J_Org_Chem-12-2240-s001.pdf]

Supporting Information

for

An effective one-pot access to polynuclear dispiroheterocyclic structures comprising pyrrolidinyloxindole and imidazothiazolotriazine moieties via a 1,3-dipolar cycloaddition strategy

Alexei N. Izmest'ev[1], Galina A. Gazieva*[1], Natalya V. Sigay[1], Sergei A. Serkov[1], Valentina A. Karnoukhova*[2], Vadim V. Kachala[1], Alexander S. Shashkov[1], Igor E. Zanin[3], Angelina N. Kravchenko[1] and Nina N. Makhova[1]

Address: [1]N. D. Zelinsky Institute of Organic Chemistry, Russian Academy of Sciences, Leninsky Prosp., 47, Moscow 119991, Russian Federation, [2]A. N. Nesmeyanov Institute of Organoelement Compounds, Russian Academy of Sciences, Vavilova Str., 28, Moscow 119991, Russian Federation and [3]Voronezh State University, Universitetskaya Pl., 1, Voronezh 394000, Russian Federation

Email: Galina A. Gazieva* - gaz@ioc.ac.ru; Valentina A. Karnoukhova* - v_a_k_h_h@mail.ru

*Corresponding author

Experimental and analytical data

Experimental..........S2
Spectra ^{1}H, ^{13}C NMR(for **1d**–**f**, **4a**–**t**), COSY, HSQC, HMBC (for **4f**) S14
HRMS spectra of compounds **4e**,**g**,**h**,**n** S42
Powder diffraction pattern for compounds **4b**–**d**,**f**–**i**,**l**–**n**..........S46
Space groups, unit cell parameters and characteristics of the investigated verification phases of compounds **4b**–**d**,**f**–**i**,**l**–**n** (Table S2) S51

Experimental

All reagents were purchased from Acros organics and used without further purification. Melting points were determined in open glass capillaries on a Gallenkamp (Sanyo) melting point apparatus. The ^{1}H NMR and ^{13}C NMR spectra were recorded on Bruker AM300 (300.13 MHz and 75.5 MHz, respectively) and Bruker DRX500 (500.13 MHz and 125.76 MHz, respectively) spectrometers using DMSO-d_6 as solvent. Chemical shifts (δ) are given in ppm from TMS as internal standard. The COSY and {^{1}H-^{13}C}HSQC, {^{1}H-^{13}C}- and {^{1}H-^{15}N}HMBC experiments were carried out on Bruker DRX500 spectrometer. Infrared (IR) spectra were recorded on a Bruker ALPHA instrument as KBr pellets. High resolution mass spectra (HRMS) were measured on a Bruker micrOTOF II instrument using electrospray ionization (ESI).

Crystallographic data: Data collection for sample **4c** was performed on a Bruker APEX DUO diffractometer, and for samples **4e** and **4r** on a Bruker SMART APEX II diffractometer, both equipped with Apex II CCD detector and graphite monochromators for MoKα radiation (λ = 0.71073 Å). Frames were integrated using the Bruker SAINT software package [1] by a narrow-frame algorithm. A semiempirical absorption correction was applied with the SADABS [2] program using the intensity data of equivalent reflections. The structures were solved with direct methods and refined by the full-matrix least-squares technique against F2hkl in anisotropic approximation with the SHELX [3] software package. Hydrogen atoms of amino groups were found from difference Fourier synthesis and refined isotropically. The positions of other hydrogen atoms were calculated, and they were refined in riding model with Uiso(H) = 1.5Ueq(Cm) and 1.2Ueq(Ci), where Ueq(Cm) and 1.2Ueq(Ci) are respectively the equivalent thermal parameters of the methyl carbon and all other carbon atoms to which corresponding H atoms are bonded. The contribution of heavily disordered solvating DMSO molecule to the diffraction in **4e** was modeled with the SQUEEZE method implemented in PLATON program [4]. Detailed crystallographic information is given in Table S1.

Table S1. Crystallographic data for crystals **4c, 4e** and **4r**.

	**4c•3MeCN**	**4e•Me$_2$SO**	**4r**
Empirical formula	$C_{45}H_{45}BrN_{10}O_3S$	$C_{40}H_{39}Br_2N_7O_4S_2$	$C_{30}H_{32}N_8O_5S_1$
Formula weight	885.88	905.72	616.69
T, K	120	120	120
Crystal system	Monoclinic	Orthorhombic	Monoclinic

Space group	$P2_1/c$	Pbca	$P2_1/c$
Z / Z'	4/1	8/1	4/1
a, Å	10.2603(5)	17.5326(19)	15.7105(6)
b, Å	11.4150(6)	20.845(2)	13.4273(5)
c, Å	36.6392(19)	22.514(2)	15.0819(5)
α, °	90	90	90
β, °	94.6830(10)	90	112.9950(10)
γ, °	90	90	90
V, $Å^3$	4276.9(4)	8228.0(15)	2928.71(19)
d_{calc}, g cm^{-3}	1.376	1.462	1.399
μ, $см^{-1}$	10.63	21.21	1.66
F(000)	1840	3696	1296
$2\theta_{max}$, °	54	61	50
Refls collected	59467	141983	38747
Independent refls	9330	12590	9040
Observed refls [I>2δ(I)]	8271	7565	7189
R1	0.0564	0.0466	0.0394
wR2	0.1261	0.1290	0.1022
GOF	1.161	1.012	1.018
Residual density, e $Å^{-3}$ (d_{max}/d_{min})	0.787/-0.833	0.749/-0.755	0.497/-0.230

Crystallographic data have been deposited to the Cambridge Crystallographic Data Centre, CCDC numbers 1473022-1473023 and 1491674. Copies of the data can be obtained free of charge via

http://www.ccdc.cam.ac.uk/data_request/cif, or by e mailing data_request@ccdc.cam.ac.uk, or by contacting The Cambridge Crystallographic Data Centre, 12 Union Road, Cambridge CB2 1EZ, UK; fax: +44(0)1223-336033.

1. Bruker, SAINT v7.23A, 2005.
2. Sheldrick, G. M. SADABS v2008/1, Bruker/Siemens Area Detector Absorption Correction Program, 2008.
3. Sheldrick, G. M. *Acta Crystallogr. Sect. A* **2008**, *64*, 112-122.
4. Spek, A. L. *Acta Crystallogr. Sect. D* **2009**, *65*, 148-155.

Powder diffraction data: High-quality experimental powder X-ray diffraction data for compounds **4b–d**,**f–i**,**l–n** were obtained with a PANalytical EMPYREAN diffractometer (fine-focus sealed tube, CuKα1 radiation (λ = 1.5406 Å), Johanson‘s Hybrid Ge{111} monochromator for the primary beam, Bragg–Brentano geometry) using a position-sensitive detector PIXcel1D. The patterns were scanned in reflection mode, θ/2θ continuously scanned over the angular range 5°–55° (2θ) with a step 0.013° (2θ) and counting time of 1000 s $step^{-1}$. Preferred orientation effects were reduced by grinding. Alignment and calibration were checked using Al_2O_3 (SRM676). Diffraction data were collected at room temperature (296 K). The extraction of peak positions for indexing was performed with the Pawley method. Patterns indexing was carried out by means of the program Ito or TREOR. Unit-cell parameters were refined by least-squares fitting of Bragg’s equation to the position of the diffraction lines. All calculations for the refinement of the diffraction patterns and refine of the unit cell parameters were performed using complex programs available in PC software “HighScore Plus” supplied by PANalytical EMPYREAN (Version: 3.0.t (3.0.5), Date 30-01-2012, produced by PANalytical B.V. Amelo, The Netherlands). The experimental powder XRD data and cell parameters obtained for compounds **4b–d**,**f–i**,**l–n**), are deposited at the PDF base of the International Centre for Diffraction Data (ICDD). Space groups, unit cell parameters and characteristics of the investigated verification phases are shown in Table S2.

General Procedure for the synthesis of compounds 1d–f: The corresponding aromatic aldehyde (2 mmol) was added to a suspension of imidazothiazolotriazine hydrobromide **5** (2 mmol) and anhydrous NaOAc (0.33 g, 4 mmol) in glacial AcOH (15 mL), and the mixture was heated at 55 °C with stirring for 12 h. The precipitate of compound **1** which formed upon cooling the reaction mixture was filtered off, washed with acetic acid and water, and dried.

(Z)-1,3-Dimethyl-6-(4-nitrobenzylidene)-3,3a,9,9a-tetrahydroimidazo[4,5-e]thiazolo[3,2-b]-1,2,4-triazine-2,7(1H,6H)-dione (1d): yellowish solid (66% yield); m.p. 262-263 °C (from acetic acid); ^{1}H NMR (300 MHz, DMSO-d_6): δ=2.60 (s, 3H; NMe), 2.78 (s, 3H; NMe), 4.78 (dd, *J*=5.7 Hz, *J*=1.8 Hz, 1H; 9a-H), 4.92 (d, J=5.7 Hz, 1H; 3a-H), 6.97 (d, *J*=1.8 Hz, 1H; NH), 7.86 (d, *J*=8.6 Hz, 2H; Ar-H), 7.90 ppm (s, 1H; =CH), 8.33 (d, *J*=8.6 Hz, 2H; Ar-H); ^{13}C NMR (75 MHz, DMSO-d_6): δ=26.8, 27.6 (NMe), 65.8,

66.2 (C-3a, C-9a), 123.4, 124.1, 128.1, 130.5, 139.5, 147.2, 147.7 (C=C, Ar, C=N), 158.5, 160.3 (C=O) ppm; IR (KBr): ν=3437, 3189 (NH), 3112 (Ar), 2922 (Alk), 1727, 1684, 1646, 1612 (C=O, C=N, C=C), 1509, 1338 cm^{-1} (NO_2); HRMS (ESI): *m/z* calcd for $C_{15}H_{14}N_6O_4S+H^+$: 375.0870 [$M+H^+$]; found: 375.0867.

(Z)-1,3-Diethyl-6-(4-nitrobenzylidene)-3,3a,9,9a-tetrahydroimidazo[4,5-e]thiazolo[3,2-b]-1,2,4-triazine-2,7(1H,6H)-dione (1e): yellowish solid (70% yield); m.p. 247-249 °C (from acetic acid); ^1H NMR (300 MHz, DMSO-d_6): δ=0.97 (t, *J*=7.0 Hz, 3H; Me), 1.15 (t, *J*=7.0 Hz, 3H; Me), 3.10-3.17 (m, 3H; NCH_2), 3.34-3.39 (m, 1H; NCH_2), 4.92 (dd, *J*=5.9 Hz, *J*=2.3 Hz, 1H; 9a-H), 4.90 (d, *J*=5.9 Hz, 1H; 3a-H), 6.93 (d, *J*=2.3 Hz, 1H; NH), 7.86 (d, *J*=8.6 Hz, 2H; Ar-H), 7.90 (s, 1H; =CH), 8.33 ppm (d, *J*=8.6 Hz, 2H; Ar-H); ^{13}C NMR (75 MHz, DMSO-d_6): δ=12.8, 13.4 (Me), 34.4, 35.1 (NCH_2), 63.7, 64.5 (C-3a, C-9a), 123.3, 124.2, 128.2, 130.7, 139.5, 147.2, 147.8 (C=C, Ar, C=N), 157.6, 160.4 ppm (C=O); IR (KBr): ν=3443, 3211 (NH), 3120 (Ar), 2974, 2935 (Alk), 1727, 1697, 1645, 1611 (C=O, C=N, C=C), 1515, 1341 cm^{-1} (NO_2); HRMS (ESI): *m/z* calcd for $C_{17}H_{18}N_6O_4S+H^+$: 403.1183 [$M+H^+$]; found: 403.1181.

(Z)-6-(2,4-Dichlorobenzylidene)-1,3-diethyl-3,3a,9,9a-tetrahydroimidazo[4,5-e]thiazolo[3,2-b]-1,2,4-triazine-2,7(1H,6H)-dione (1f): pale pink solid (53% yield); m.p. 226-228 °C (from acetic acid); ^1H NMR (300 MHz, DMSO-d_6): δ=0.97 (t, *J*=7.1 Hz, 3H; Me), 1.14 (t, *J*=7.2 Hz, 3H; Me), 3.06-3.15 (m, 3H; NCH_2), 3.32-3.38 (m, 1H; NCH_2), 4.90 (dd, *J*=5.9 Hz, *J*=2.0 Hz, 1H; 9a-H), 4.97 (d, *J*=5.9 Hz, 1H; 3a-H), 6.91 (d, *J*=2.0 Hz, 1H; NH), 7.57-7.64 (m, 2H; Ar-H), 7.83 ppm (s, 2H; =CH, Ar-H); ^{13}C NMR (75 MHz, DMSO-d_6): δ=12.7, 13.3 (Me), 34.4, 35.1 (NCH_2), 63.6, 64.4 (C-3a, C-9a), 123.2, 124.7, 128.1, 129.75, 129.84, 130.1, 134.95, 135.04, 147.7 (C=C, Ar, C=N), 157.6, 160.3 ppm (C=O); IR (KBr): ν=3186 (NH), 3071, 3018 (Ar), 2974, 2932 (Alk), 1730, 1669, 1647, 1604 cm^{-1} (C=O, C=N, C=C); HRMS (ESI): *m/z* calcd for $C_{17}H_{17}Cl_2N_5O_2S+H^+$: 426.0553 [$M+H^+$]; found: 426.0548.

General procedure for the synthesis of compounds 4a–t: A mixture of corresponding compound **1** (0.5 mmol), isatin **3** (0.5 mmol), and sarcosine (0.5 mmol) in acetonitrile (40 mL) (for **1a–c**) or in a mixture of acetonitrile (30 mL) and chloroform (10 ml) (for **1d–f**) was refluxed with stirring for 36 or 72 h for starting compounds **1a–c** and **1d–f**, respectively. Then the reaction mixture was cooled to room temperature, the precipitate of compounds **4a–e**,**j**,**o** was filtered off and washed with acetonitrile and water. The reaction mixture of compounds **4f–i**,**k–n**,**p-t** was concentrated on a rotary evaporator to half of an original volume. After cooling, the precipitate was filtered off and washed with water. Recrystallization from acetonitrile or methanol (**4n**) gave dispirocompounds **4a–t**.

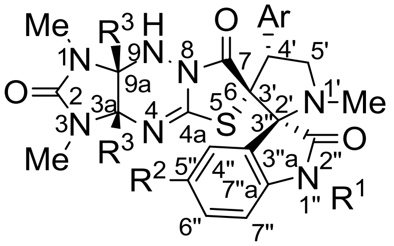

4a–**o**, R^3 = Ph; **4p**–**t**, R^3 = H

4'-(4-Bromophenyl)-1,1',3-trimethyl-3a,9a-diphenyl-3,3a,9,9a-tetrahydrodispiro[imidazo[4,5-e]thiazolo[3,2-b]-1,2,4-triazine-6,3'-pyrrolidine-2',3''-indole]-2,2'',7(1H,1''H)-trione (4a): white solid (78% yield); m.p. 231-233 °C (from acetonitrile); ^{1}H NMR (300 MHz, DMSO-d_6): δ=2.15 (s, 3H; N(1')Me), 2.41 (s, 3H; N(1)Me), 2.63 (s, 3H; N(3)Me), 3.56 (t, *J*=8.4 Hz, 1H, 5′-H), 3.95 (t, *J*=9.2 Hz, 1H; 5′-H), 4.45 (t, *J*=8.7 Hz, 1H; 4′-H), 6.13 (d, *J*=7.6 Hz, 2H; o-Ph-H), 6.55 (d, *J*=7.6 Hz, 2H; o-Ph-H), 6.92 (t, *J*=7.4 Hz, 2H; m-Ph-H), 7.00-7.14 (m, 6H; Ph-H, 5′′-H, 7′′-H), 7.38-7.41 (m, 3H; o-Ar-H, 4′′-H), 7.51 (t, *J*=7.6 Hz, 1H; 6′′-H), 7.61 (d, *J*=7.9 Hz, 2H; m-Ar-H), 7.84 (s, 1H; N(9)H), 10.84 ppm (s, 1H; N(1'')H); ^{13}C NMR (75 MHz, DMSO-d_6): δ=25.2, 25.9 (N(3)Me, N(1)Me), 34.9 (N(1')Me), 50.2 (C-4′), 57.1 (C-5′), 68.4 (C-6), 79.3, 79.8, 82.8 (C-3a, C-9a, C-2′), 109.9 (C-7′′), 121.0, 122.2, 123.1, 126.1, 126.6, 126.9, 127.1, 127.6, 128.1, 130.7, 131.7, 131.8, 132.3, 133.7, 134.7, 137.5 (Ph, Ar, C-3a′′, C-4′′, C-5′′, C-6′′), 143.9 (C-7a′′), 146.7 (C-4a), 159.1 (C-2), 167.1 (C-7), 176.2 ppm (C-2′′). IR (KBr): ν=3423, 3150 (NH), 3081, 3065, 3033 (Ar), 2947, 2908, 2869, 2858 (Alk), 1720, 1709, 1644 (C=O), 1621 cm^{-1} (C=N); HRMS (ESI): *m/z* calcd for $C_{37}H_{32}BrN_7O_3S+H^+$: 734.1543 [M+H$^+$], found: 734.1538.

4'-(4-Bromophenyl)-1,1',1'',3-tetramethyl-3a,9a-diphenyl-3,3a,9,9a-tetrahydrodispiro[imidazo[4,5-e]thiazolo[3,2-b]-1,2,4-triazine-6,3'-pyrrolidine-2',3''-indole]-2,2'',7(1H,1''H)-trione (4b): white solid (69% yield); m.p. 269-271 °C (from acetonitrile); ^{1}H NMR (300 MHz, DMSO-d_6): δ=2.12 (s, 3H; N(1')Me), 2.41 (s, 3H; N(1)Me), 2.64 (s, 3H; N(3)Me), 3.21 (s, 3H; N(1'')Me), 3.60 (t, *J*=8.3 Hz, 1H; 5′-H), 3.98 (t, *J*=9.1 Hz, 1H; 5′-H), 4.48 (t, *J*=8.5 Hz, 1H; 4′-H), 6.12 (d, *J*=7.6 Hz, 2H; o-Ph-H), 6.56 (d, *J*=7.3 Hz, 2H; o-Ph-H), 6.92 (t, *J*=7.3 Hz, 2H; m-Ph-H), 7.03-7.28 (m, 6H; Ph-H, 5′′-H, 7′′-H), 7.42-7.44 (m, 3H; o-Ar-H, 4′′-H), 7.62-7.64 (m, 3H; m-Ar-H, 6′′-H), 7.88 ppm (s, 1H; N(9)H); ^{13}C NMR (75 MHz, DMSO-d_6): δ=25.2, 25.7 (N(3)Me, N(1)Me), 25.9 (N(1′′)Me), 34.9 (N(1')Me), 50.2 (C-4′), 57.3 (C-5′), 68.5 (C-6), 79.3, 79.6, 82.9 (C-3a, C-9a, C-2′), 108.9 (C-7′′), 121.1, 122.4, 123.0, 126.5, 126.7, 127.1, 127.1, 127.2, 127.6, 128.1, 130.9, 131.7, 131.9, 133.8, 134.7, 137.5 (Ph, Ar, C-3a′′, C-4′′, C-5′′, C-6′′), 145.2 (C-7a′′), 146.5 (C-4a), 159.2 (C-2), 167.0 (C-7), 174.5 ppm (C-2′′); IR (KBr): ν=3413, 3252 (NH), 3059, 3027 (Ar), 2940 (Alk), 1715 (broad), 1640 (C=O), 1612 cm^{-1} (C=N); HRMS (ESI): *m/z* calcd for $C_{38}H_{34}BrN_7O_3S+H^+$: 748.1700 [M+H$^+$]; found: 748.1695.

4'-(4-Bromophenyl)-1''-ethyl-1,1',3-trimethyl-3a,9a-diphenyl-3,3a,9,9a-tetrahydrodispiro[imidazo[4,5-e]thiazolo[3,2-b]-1,2,4-triazine-6,3'-pyrrolidine-2',3''-indole]-2,2'',7(1H,1''H)-trione (4c): white solid

(54% yield); m.p. 257-258 °C (from acetonitrile); ^{1}H NMR (500 MHz, DMSO-d_6): δ=1.18 (t, *J*=7.0 Hz, 3H: Me), 2.11 (s, 3H; N(1')Me), 2.40 (s, 3H; N(1)Me), 2.63 (s, 3H; N(3)Me), 3.59 (t, *J*=8.4 Hz, 1H; 5'-H), 3.71-3.75 (m, 1H; N(1")CH_2), 3.84-3.88 (m, 1H; N(1")CH_2), 3.97 (t, *J*=9.4 Hz, 1H, 5'-H), 4.48 (t, *J*=8.8 Hz, 1H; 4'-H), 6.12 (d, *J*=7.6 Hz, 2H; o-Ph-H), 6.54 (d, *J*=7.3 Hz, 2H; o-Ph-H), 6.93 (t, *J*=7.5 Hz, 2H; m-Ph-H), 7.02-7.13 (m, 4H; Ph-H), 7.17 (t, *J*=7.4 Hz, 1H; 5″-H), 7.31 (d, *J*=7.8 Hz, 1H; 7″-H), 7.42-7.46 (m, 3H; o-Ar-H, 4″-H), 7.59-7.64 (m, 3H; m-Ar-H, 6″-H), 7.85 ppm (s, 1H; N(9)H); ^{13}C NMR (125 MHz, DMSO-d_6): δ=12.7 (Me), 25.3, 26.0 (N(3)Me, N(1)Me), 34.1 (N(1")CH_2), 34.8 (N(1')Me), 50.1 (C-4'), 57.50 (C-5'), 68.7 (C-6), 79.3, 79.4, 82.8 (C-3a, C-9a, C-2'), 109.1 (C-7"), 121.1, 122.6, 122.9, 126.7, 126.8, 127.2, 127.2, 127.7, 128.2, 131.0, 131.8, 132.0, 133.9, 134.8, 137.6 (Ph, Ar, C-3a", C-4", C-5", C-6"), 144.1 (C-7a"), 146.5 (C4a), 159.2 (C-2), 167.0 (C-7), 174.2 ppm (C-2"); IR (KBr): ν=3435 (NH), 3054, 3042 (Ar), 2975, 2945 (Alk), 1717, 1705, 1649 (C=O), 1608 cm^{-1} (C=N); HRMS (ESI): *m/z* calcd for $C_{39}H_{36}BrN_7O_3S+H^+$: 762.1856 [M+$H^+$]; found: 762.1851.

1''-Allyl-4'-(4-bromophenyl)-1,1',3-trimethyl-3a,9a-diphenyl-3,3a,9,9a-tetrahydrodispiro[imidazo[4,5-e]thiazolo[3,2-b]-1,2,4-triazine-6,3'-pyrrolidine-2',3''-indole]-2,2',7(1H,1''H)-trione (4d): white solid (66% yield); m.p. 219-221 °C (from acetonitrile); ^{1}H NMR (300 MHz, DMSO-d_6): δ=2.14 (s, 3H; N(1')Me), 2.41 (s, 3H; N(1)Me), 2.65 (s, 3H; N(3)Me), 3.60 (t, *J*=8.3 Hz, 1H; 5'-H), 3.97 (t, *J*=9.5 Hz, 1H; 5'-H), 4.30-4.52 (m, 3H; 4'-H, N(1")CH_2), 5.13-5.30 (m, 2H; C=CH_2), 5.82-5.91 (m, 1H; N(1")$CH_2C\underline{H}$), 6.15 (d, *J*=7.5 Hz, 2H; o-Ph-H), 6.56 (d, *J*=7.4 Hz, 2H; o-Ph-H), 6.91 (t, *J*=7.7 Hz, 2H; m-Ph-H), 7.05-7.20 (m, 6H; Ph-H, 5"-H, 7"-H), 7.40-7.49 (m, 3H; o-Ar-H, 4″-H), 7.55-7.63 (m, 3H; m-Ar-H, 6″-H), 7.71 ppm (s, 1H; N(9)H); ^{13}C NMR (125 MHz, DMSO-d_6): δ=25.3, 26.0 (N(3)Me, N(1)Me), 35.0 (N(1')Me), 41.4 (N(1")CH_2), 50.1 (C-4'), 57.4 (C-5'), 68.6 (C-6), 79.4, 79.7, 82.9 (C-3a, C-9a, C-2'), 109.7 (C-7"), 117.1, 121.1, 122.4, 123.0, 126.7, 127.2, 127.7, 128.1, 128.2, 130.9, 131.5, 131.8, 131.8, 131.9, 132.4, 133.8, 134.7, 137.5 (Ph, Ar, HC=CH_2, C-3a", C-4", C-5", C-6"), 144.4 (C-7a"), 146.5 (C-4a), 159.2 (C-2), 167.0 (C-7), 174.3 ppm (C-2"); IR (KBr): ν=3435 (NH), 3058 (Ar), 2972, 2944, 2913 (Alk), 1712 (broad), 1642 (C=O), 1611 cm^{-1} (C=N); HRMS (ESI): *m/z* calcd for $C_{40}H_{36}BrN_7O_3S+H^+$: 774.1856 [M+$H^+$]; found: 774.1858.

5''-Bromo-4'-(4-bromophenyl)-1,1',1'',3-tetramethyl-3a,9a-diphenyl-3,3a,9,9a-tetrahydrodispiro[imidazo[4,5-e]thiazolo[3,2-b]-1,2,4-triazine-6,3'-pyrrolidine-2',3''-indole]-2,2'',7(1H,1''H)-trione (4e): white solid (55% yield); m.p. 269-270 °C (from acetonitrile); ^{1}H NMR (300 MHz, DMSO-d_6): δ=2.14 (s, 3H; N(1')Me), 2.43 (s, 3H; N(1)Me), 2.65 (s, 3H; N(3)Me), 3.22 (s, 3H; N(1")Me), 3.63 (t, *J*=8.3 Hz, 1H; 5'-H), 3.95 (t, *J*=9.6 Hz, 1H; 5'-H), 4.49 (t, *J*=8.7 Hz, 1H; 4'-H), 6.10 (d, *J*=7.9 Hz, 2H; o-Ph-H), 6.68 (d, *J*=8.1 Hz, 2H; o-Ph-H), 6.96 (t, *J*=7.3 Hz, 2H; m-Ph-H), 7.02-7.15 (m, 4H; Ph-H), 7.28 (d, *J*=7.5 Hz, 1H; 7"-H), 7.46 (d, *J*=8.4 Hz, 2H; o-Ar-H), 7.55 (s, 1H; 4"-H), 7.63 (d, *J*=8.3 Hz, 2H; m-Ar-H), 7.89 (d, *J*=7.3 Hz, 1H; 6"-H), 8.26 ppm (s, 1H; N(9)H); ^{13}C NMR (75 MHz, DMSO-d_6): δ=25.3, 25.9, 25.9 (N(3)Me, N(1)Me, N(1")Me), 34.9 (N(1')Me), 49.6 (C-4'), 57.5 (C-5'), 68.4 (C-6), 79.2, 79.7, 83.1 (C-3a, C-9a, C-2'), 111.0 (C-7"), 115.2, 121.1, 124.4, 126.9, 127.0, 127.1,

127.5, 127.6, 128.0, 129.0, 131.8, 131.9, 133.8, 133.9, 134.6, 137.3 (Ph, Ar, C-3a", C-4", C-5", C-6"), 144.54 (C-7a"), 145.9 (C-4a), 159.1 (C-2), 166.6 (C-7), 173.9 ppm (C-2"); IR (KBr): ν=3400, 3247 (NH), 3055 (Ar), 2973, 2940 (Alk), 1713, 1704, 1638 (C=O), 1605 cm^{-1} (C=N); HRMS (ESI): *m/z* calcd for $C_{38}H_{33}Br_2N_7O_3S+H^+$: 826.0805 [$M+H^+$]; found: 826.0799.

1,1',3-Trimethyl-4'-(4-nitrophenyl)-3a,9a-diphenyl-3,3a,9,9a-tetrahydrodispiro[imidazo[4,5-e]thiazolo[3,2-b]-1,2,4-triazine-6,3'-pyrrolidine-2',3''-indole]-2,2'',7(1H,1''H)-trione (4f): white solid (83% yield); m.p. 265-267 °C (from acetonitrile); ^1H NMR (300 MHz, DMSO-d_6): δ=2.17 (s, 3H; N(1')Me), 2.38 (s, 3H; N(1)Me), 2.65 (s, 3H; N(3)Me), 3.61 (t, *J*=8.2 Hz, 1H; 5'-H), 4.02 (t, *J*=9.2 Hz, 1H; 5'-H), 4.63 (t, *J*=8.4 Hz, 1H; 4'-H), 6.14 (d, *J*=7.6 Hz, 2H; o-Ph-H), 6.57 (d, *J*=7.4 Hz, 2H; o-Ph-H), 6.91 (t, *J*=7.5 Hz, 2H; m-Ph-H), 7.01-7.13 (m, 6H; Ph-H, 5"-H, 7"-H), 7.41 (d, *J*=7.5 Hz, 1H; 4"-H), 7.51 (t, *J*=7.7 Hz, 1H; 6"-H), 7.73 (d, *J*=8.4 Hz, 2H; o-Ar-H), 7.82 (s, 1H; N(9)H), 8.29 (d, *J*=8.4 Hz, 2H; m-Ar-H), 10.88 ppm (s, 1H; N(1")H); ^{13}C NMR (125 MHz, DMSO-d_6): δ=25.8, 26.3 (N(3)Me, N(1)Me), 35.4 (N(1')Me), 50.9 (C-4'), 57.5 (C-5'), 68.5 (C-6), 79.8, 80.3, 83.3 (C-3a, C-9a, C-2'), 110.5 (C-7"), 122.8, 123.4, 124.3, 127.2, 127.5, 127.6, 127.7, 128.1, 128.6, 131.3, 131.6, 134.2, 135.1 (Ph, Ar, C-3a", C-4", C-5", C-6"), 144.4 (C-7a"), 146.3 (ipso-Ar), 147.0 (C-4a), 147.5 (CNO_2), 159.6 (C-2), 167.3 (C-7), 176.7 ppm (C-2"); IR (KBr): ν=3499, 3414, 3240 (NH), 3060, 3034 (Ar), 2972, 2949 (Alk), 1709, 1684, 1636 (C=O), 1618 (C=N), 1520, 1349 cm^{-1} (NO_2); HRMS (ESI): *m/z* calcd for $C_{37}H_{32}N_8O_5S+H^+$: 701.2289 [$M+H^+$]; found: 701.2307.

1,1',1'',3-Tetramethyl-4'-(4-nitrophenyl)-3a,9a-diphenyl-3,3a,9,9a-tetrahydrodispiro[imidazo[4,5-e]thiazolo[3,2-b]-1,2,4-triazine-6,3'-pyrrolidine-2',3''-indole]-2,2'',7(1H,1''H)-trione (4g): yellowish solid (73% yield); m.p. 227-230 °C (from acetonitrile); ^1H NMR (300 MHz, DMSO-d_6): δ=2.13 (s, 3H; N(1')Me), 2.38 (s, 3H; N(1)Me), 2.66 (s, 3H; N(3)Me), 3.21 (s, 3H; N(1")Me), 3.65 (t, *J*=8.0 Hz, 1H; 5'-H), 4.05 (t, *J*=9.2 Hz, 1H; 5'-H), 4.66 (t, *J*=8.2 Hz, 1H; 4'-H), 6.16 (d, *J*=7.2 Hz, 2H; o-Ph-H), 6.58 (d, *J*=7.0 Hz, 2H; o-Ph-H), 6.92 (t, *J*=6.7 Hz, 2H; m-Ph-H), 7.05-7.26 (m, 6H; Ph-H, 5"-H, 7"-H), 7.47 (d, *J*=7.4 Hz, 1H; 4"-H), 7.62 (t, *J*=7.2 Hz, 1H; 6"-H), 7.71-7.76 (3 H, m, o-Ar-H, N(9)H), 8.28 ppm (2 H, d, *J*=8.0, m-Ar-H); ^{13}C NMR (75 MHz, DMSO-d_6): δ=25.2, 25.7, 25.8 (N(3)Me, N(1)Me, N(1")Me), 34.8 (N(1')Me), 50.4 (C-4'), 57.1 (C-5'), 68.0 (C-6), 79.3, 79.6, 82.7 (C-3a, C-9a, C-2'), 108.9 (C-7"), 122.1, 123.0, 123.8, 126.5, 126.6, 127.1, 127.1, 127.6, 128.0, 130.9, 131.1, 133.6, 134.5 (Ph, Ar, C-3a", C-4", C-5", C-6"), 145.2 (C-7a"), 145.6 (ipso-Ar), 146.2 (C-4a), 147.0 (CNO_2), 159.1 (C-2), 166.7 (C-7), 174.4 ppm (C-2"); IR (KBr): ν=3412, 3189 (NH), 3059, 3028 (Ar), 2945 (Alk), 1714 (broad), 1642 (C=O), 1611 (C=N), 1522, 1346 cm^{-1} (NO_2); HRMS (ESI): *m/z* calcd for $C_{38}H_{34}N_8O_5S+H^+$: 715.2446 [$M+H^+$]; found: 715.2446.

1''-Ethyl-1,1',3-trimethyl-4'-(4-nitrophenyl)-3a,9a-diphenyl-3,3a,9,9a-tetrahydrodispiro[imidazo[4,5-e]thiazolo[3,2-b]-1,2,4-triazine-6,3'-pyrrolidine-2',3''-indole]-2,2'',7(1H,1''H)-trione (4h): yellow solid (58% yield); m.p. 283-285 °C (from acetonitrile); ^1H NMR (300 MHz, DMSO-d_6): δ=1.18 (t, *J*=6.4 Hz,

3H; Me), 2.13 (s, 3H; N(1')Me), 2.37 (s, 3H; N(1)Me), 2.64 (s, 3H; N(3)Me), 3.64-3.90 (m, 3H; 5'-H, N(1'')CH_2), 4.03 (t, *J*=8.9 Hz, 1H; 5'-H), 4.66 (t, *J*=7.9 Hz, 1H; 4'-H), 6.12 (d, *J*=7.3 Hz, 2H; o-Ph-H), 6.54 (d, *J*=7.0 Hz, 2H; o-Ph-H), 6.92 (t, *J*=7.3 Hz, 2H; m-Ph-H), 7.04-7.21 (m, 5H; Ph-H, 5''-H), 7.32 (d, *J*=7.9 Hz, 1H; 7''-H), 7.46 (d, *J*=7.0 Hz, 1H; 4''-H), 7.61 (t, *J*=7.5 Hz, 1H; 6''-H), 7.75 (d, *J*=8.1 Hz, 2H; o-Ar-H), 7.88 (s, 1H; N(9)H), 8.30 ppm (d, *J*=8.1 Hz, 2H; m-Ar-H); ^{13}C NMR (75 MHz, DMSO-d_6): δ=13.0 (Me), 25.6, 26.2 (N(3)Me, N(1)Me), 34.4 (N(1'')CH_2), 35.1 (N(1')Me), 50.6 (C-4'), 57.6 (C-5'), 68.6 (C-6), 79.7, 79.7, 83.1 (C-3a, C-9a, C-2'), 109.4 (C-7''), 122.7, 123.2, 124.2, 127.0, 127.2, 127.4, 127.5, 128.0, 128.4, 131.4, 131.5, 134.1, 135.0 (Ph, Ar, C-3a'', C-4'', C-5'', C-6''), 144.4 (C-7a''), 146.1 (ipso-Ar), 146.6 (C-4a), 147.3 (CNO_2), 159.5 (C-2), 167.0 (C-7), 174.5 ppm (C-2''); IR (KBr): ν=3430, 3195, 3164 (NH), 3071, 3059 (Ar), 2975, 2943 (Alk), 1724, 1709, 1641 (C=O), 1610 (C=N), 1522, 1346 cm^{-1} (NO_2); HRMS (ESI): *m/z* calcd for $C_{39}H_{36}N_8O_5S+H^+$: 729.2602[M+H$^+$]; found: 729.2598.

1''-Allyl-1,1',3-trimethyl-4'-(4-nitrophenyl)-3a,9a-diphenyl-3,3a,9,9a-tetrahydrodispiro[imidazo[4,5-e]thiazolo[3,2-b]-1,2,4-triazine-6,3'-pyrrolidine-2',3''-indole]-2,2'',7(1H,1''H)-trione (4i): white solid (75% yield); m.p. 254-255 °C (from acetonitrile); ^{1}H NMR (300 MHz, DMSO-d_6): δ=2.16 (s, 3H; N(1')Me), 2.38 (s, 3H; N(1)Me), 2.66 (s, 3H; N(3)Me), 3.67 (t, *J*=8.3 Hz, 1H; 5'-H), 4.04 (t, *J*=9.2 Hz, 1H; 5'-H), 4.32-4.52 (m, 2H; N(1'')CH_2), 4.68 (t, *J*=8.7 Hz, 1H; 4'-H), 5.14-5.30 (m, 2H; C=CH_2), 5.82-5.92 (m, 1H; N(1'')$CH_2C\underline{H}$), 6.14 (d, *J*=7.7 Hz, 2H; o-Ph-H), 6.56 (d, *J*=7.6 Hz, 2H; o-Ph-H), 6.91 (t, *J*=7.6 Hz, 2H; m-Ph-H), 7.03-7.22 (m, 6H; Ph-H, 5''-H, 7''-H), 7.49 (d, *J*=7.5 Hz, 1H; 4''-H), 7.60 (t, *J*=7.7 Hz, 1H; 6''-H), 7.75 (d, *J*=8.3 Hz, 2H; o-Ar-H), 7.89 (s, 1H; N(9)H), 8.30 ppm (d, *J*=8.3 Hz, 2H; m-Ar-H); ^{13}C NMR (75 MHz, DMSO-d_6): δ=25.3, 25.9 (N(3)Me, N(1)Me), 34.9 (N(1')Me), 41.4 (N(1'')CH_2), 50.4 (C-4'), 57.2 (C-5'), 68.1 (C-6), 79.4, 79.6, 82.8 (C-3a, C-9a, C-2'), 109.7 (C-7''), 117.1, 122.2, 123.1, 123.9, 126.7, 126.8, 127.2, 127.7, 128.2, 131.0, 131.2, 131.4, 133.8, 134.6 (Ph, Ar, HC=CH_2, C-3a'', C-4'', C-5'', C-6''), 144.3 (C-7a''), 145.7 (ipso-Ar), 146.3 (C-4a), 147.1 (CNO_2), 159.2 (C-2), 166.7 (C-7), 174.3 ppm (C-2''); IR (KBr): ν=3435, 3153 (NH), 3079, 3028 (Ar), 2975, 2947, 2915 (Alk), 1705 (broad), 1641 (C=O), 1609 (C=N), 1521, 1346 cm^{-1} (NO_2); HRMS (ESI): *m/z* calcd for $C_{40}H_{36}N_8O_5S+H^+$: 741.2602 [M+H$^+$]; found: 741.2586.

5''-Bromo-1,1',1'',3-tetramethyl-4'-(4-nitrophenyl)-3a,9a-diphenyl-3,3a,9,9a-tetrahydrodispiro[imidazo[4,5-e]thiazolo[3,2-b]-1,2,4-triazine-6,3'-pyrrolidine-2',3''-indole]-2,2'',7(1H,1''H)-trione (4j): yellow solid (71% yield); m.p. 260-262 °C (from acetonitrile); ^{1}H NMR (300 MHz, DMSO-d_6): δ=2.13 (s, 3H; N(1')Me), 2.36 (s, 3H; N(1)Me), 2.63 (s, 3H; N(3)Me), 3.19 (s, 3H; N(1'')Me), 3.65 (t, *J*=8.4 Hz, 1H; 5'-H), 3.98 (t, *J*=9.3 Hz, 1H; 5'-H), 4.64 (t, *J*=8.5 Hz, 1H; 4'-H), 6.06 (d, *J*=7.7 Hz, 2H; o-Ph-H), 6.64 (d, *J*=7.6 Hz, 2H; o-Ph-H), 6.92 (t, *J*=7.7 Hz, 2H; m-Ph-H), 6.99-7.12 (m, 4H; Ph-H), 7.26 (d, *J*=8.4 Hz, 1H; 7''-H), 7.52 (d, *J*=1.6 Hz, 1H; 4''-H), 7.76 (d, *J*=8.6 Hz, 2H; o-Ar-H), 7.87 (dd, ^3J=8.4 Hz, ^4J=1.6 Hz, 1H; 6''-H), 8.27-8.29 ppm (m, 3H; m-Ar-H, N(9)H); ^{13}C NMR (75 MHz, DMSO-d_6): δ=25.3, 25.9, 25.9 (N(3)Me, N(1)Me, N(1'')Me), 34.9 (N(1')Me), 49.9 (C-4'), 57.4 (C-5'), 68.0 (C-6), 79.2, 79.7, 83.1 (C-3a, C-9a, C-2'), 111.1 (C-7''), 115.3, 123.9, 124.2, 126.9, 127.0, 127.2,

127.5, 127.7, 128.0, 129.0, 131.2, 133.8, 133.9, 134.5 (Ph, Ar, C-3a", C-4", C-5", C-6"), 144.5 (C-7a"), 145.5(ipso-Ar), 145.7 (C-4a), 147.0 (CNO_2), 159.1 (C-2), 166.3 (C-7), 173.9 ppm (C-2"); IR (KBr): ν=3475, 3155 (NH), 3091, 3067 (Ar), 2970, 2943 (Alk), 1719, 1688, 1636 (C=O), 1608 (C=N), 1522, 1343 cm^{-1} (NO_2); HRMS (ESI): *m/z* calcd for $C_{38}H_{33}BrN_8O_5S+Na^+$: 815.1370 $[M+Na^+]$; found: 815.1340.

1,1',3-Trimethyl-3a,4',9a-triphenyl-3,3a,9,9a-tetrahydrodispiro[imidazo[4,5-e]thiazolo[3,2-b]-1,2,4-triazine-6,3'-pyrrolidine-2',3''-indole]-2,2'',7(1H,1''H)-trione (4k): white solid (73% yield); m.p. 207-209 °C (from acetonitrile); ^1H NMR (300 MHz, DMSO-d_6): δ=2.17 (s, 3H; N(1')Me), 2.40 (s, 3H; N(1)Me), 2.63 (s, 3H; N(3)Me), 3.55 (t, *J*=8.2 Hz, 1H; 5′-H), 4.02 (t, *J*=9.0 Hz, 1H; 5′-H), 4.48 (t, *J*=8.5 Hz, 1H; 4′-H), 6.12 (d, *J*=7.4 Hz, 2H; o-Ph-H), 6.55 (d, *J*=7.3 Hz, 2H; o-Ph-H), 6.92 (t, *J*=7.3 Hz, 2H; m-Ph-H), 7.01-7.12 (m, 6H; Ph-H, 5″-H, 7″-H), 7.32-7.54 (m, 7H; 4′-Ph, 4″-H, 6″-H), 7.75 (s, 1H; N(9)H), 10.79 ppm (s, 1H; N(1″)H); ^{13}C NMR (75 MHz, DMSO-d_6): δ=25.1, 25.84 (N(3)Me, N(1)Me), 34.9 (N(1')Me), 50.5 (C-4′), 57.3 (C-5′), 68.8 (C-6), 79.1, 79.9, 82.9 (C-3a, C-9a, C-2′), 109.8 (C-7″), 122.1, 123.2, 126.68, 126.73, 127.1, 127.1, 127.5, 127.6, 128.0, 128.8, 129.6, 130.6, 133.8, 134.7, 138.1 (Ph, C-3a″, C-4″, C-5″, C-6″), 143.9 (C-7a″), 146.7 (C-4a), 159.1 (C-2), 167.1 (C-7), 176.2 ppm (C-2″); IR (KBr): ν=3416, 3169 (NH), 3061, 3030 (Ar), 2979, 2939 (Alk), 1718 (br), 1639 (br) cm^{-1} (C=O, C=N); HRMS (ESI): *m/z* calcd for $C_{37}H_{33}N_7O_3S+H^+$: 656.2438 $[M+H^+]$; found: 656.2432.

1,1',1'',3-Tetramethyl-3a,4',9a-triphenyl-3,3a,9,9a-tetrahydrodispiro[imidazo[4,5-e]thiazolo[3,2-b]-1,2,4-triazine-6,3'-pyrrolidine-2',3''-indole]-2,2'',7(1H,1''H)-trione (4l): white solid (72% yield); m.p. 265-266 °C (from acetonitrile); ^1H NMR (300 MHz, DMSO-d_6): δ=2.13 (s, 3H; N(1')Me), 2.39 (s, 3H; N(1)Me), 2.63 (s, 3H; N(3)Me), 3.21 (s, 3H; N(1")Me), 3.59 (t, *J*=8.2 Hz, 1H; 5′-H), 4.04 (t, *J*=8.9 Hz, 1H; 5′-H), 4.50 (t, *J*=8.5 Hz, 1H; 4′-H), 6.09 (d, *J*=7.5 Hz, 2H; o-Ph-H), 6.54 (d, *J*=7.3 Hz, 2H; o-Ph-H), 6.93 (t, *J*=7.5 Hz, 2H; m-Ph-H), 7.02-7.49 (m, 12H; Ph-H, 4″-H, 5″-H, 7″-H), 7.64 (t, *J*=7.5 Hz, 1H; 6″-H), 7.86 ppm (s, 1H; N(9)H); ^{13}C NMR (75 MHz, DMSO-d_6): δ=25.1, 25.6, 25.8 (N(3)Me, N(1)Me, N(1")Me), 34.8 (N(1')Me), 50.5 (C-4′), 57.5 (C-5′), 68.8 (C-6), 79.1, 79.7, 82.9 (C-3a, C-9a, C-2′), 108.8 (C-7″), 122.4, 122.8, 126.3, 126.7, 127.1, 127.1, 127.1, 127.5, 127.7, 128.0, 128.8, 129.6, 130.8, 133.7, 134.6, 138.0 (Ph, C-3a″, C-4″, C-5″, C-6″), 145.2 (C-7a″), 146.4 (C-4a), 159.1 (C-2), 167.0 (C-7), 174.4 ppm (C-2″); IR (KBr): ν=3412, 3161 (NH), 3073, 3054, 3027 (Ar), 2940, 2907, 2861 (Alk), 1715 (br), 1638 (C=O), 1611 cm^{-1} (C=N); HRMS (ESI): *m/z* calcd for $C_{38}H_{35}N_7O_3S+H^+$: 670.2595 $[M+H^+]$; found: 670.2584.

1''-Ethyl-1,1',3-trimethyl-3a,4',9a-triphenyl-3,3a,9,9a-tetrahydrodispiro[imidazo[4,5-e]thiazolo[3,2-b]-1,2,4-triazine-6,3'-pyrrolidine-2',3''-indole]-2,2'',7(1H,1''H)-trione (4m): white solid (59% yield); m.p. 252-253 °C (from acetonitrile); ^1H NMR (300 MHz, DMSO-d_6): δ=1.17 (t, *J*=7.0 Hz, 3H; Me), 2.12 (s, 3H; N(1')Me), 2.37 (s, 3H; N(1)Me), 2.61 (s, 3H; N(3)Me), 3.57 (t, *J*=8.2 Hz, 1H; 5′-H), 3.68-3.75 (m, 1H; N(1")CH_2), 3.82-3.89 (m, 1H; N(1")CH_2), 4.02 (t, *J*=9.3 Hz, 1H; 5′-H), 4.49 (t, *J*=8.8 Hz, 1H; 4′-H), 6.08 (d, *J*=7.7 Hz, 2H; o-Ph-H), 6.51 (d, *J*=7.3 Hz, 2H; o-Ph-H), 6.92 (t, *J*=7.7 Hz, 2H; m-Ph-H), 7.00-

7.18 (m, 5H; Ph-H, 7″-H), 7.31 (t, *J*=8.2 Hz, 2H; o-Ph-H), 7.39-7.47 (m, 5H; 4′-Ph-H, 5″-H, 4″-H), 7.60 (t, *J*=7.6 Hz, 1H; 6″-H), 7.81 ppm (s, 1H; N(9)H); ^{13}C NMR (75 MHz, DMSO-d_6): δ=12.6 (Me), 25.2, 25.9 (N(3)Me, N(1)Me), 34.1 (N(1")CH_2), 34.8 (N(1')Me), 50.4 (C-4'), 57.7 (C-5'), 69.1 (C-6), 79.2, 79.5, 83.0 (C-3a, C-9a, C-2'), 109.0 (C-7"), 122.8, 126.6, 126.8, 127.1, 127.2, 127.6, 127.8, 128.1, 128.9, 129.7, 130.9, 133.8, 134.7, 138.1 (Ph, C-3a", C-4", C-5", C-6"), 144.11 (C-7a"), 146.5 (C-4a), 159.2 (C-2), 167.1 (C-7), 174.2 ppm (C-2"); IR (KBr): ν=3412, 3163 (NH), 3072, 3051 (Ar), 2975, 2938, 2908 (Alk), 1712, 1638 (C=O), 1610 cm^{-1} (C=N); HRMS (ESI): *m/z* calcd for $C_{39}H_{37}N_7O_3S+H^+$: 684.2751 [M+H$^+$]; found: 684.2752.

1''-Allyl-1,1',3-trimethyl-3a,4',9a-triphenyl-3,3a,9,9a-tetrahydrodispiro[imidazo[4,5-e]thiazolo[3,2-b]-1,2,4-triazine-6,3'-pyrrolidine-2',3''-indole]-2,2'',7(1H,1''H)-trione (4n): white solid (62% yield); m.p. 249-250 °C (from methanol); ^{1}H NMR (300 MHz, DMSO-d_6): δ=2.15 (s, 3H; N(1')Me), 2.39 (s, 3H; N(1)Me), 2.63 (s, 3H; N(3)Me), 3.59 (t, *J*=8.3 Hz, 1H; 5′-H), 4.04 (t, *J*=9.3 Hz, 1H; 5′-H), 4.28-4.55 (m, 3H; 4'-H, N(1")CH_2), 5.13-5.31 (m, 2H; C=CH_2), 5.80-5.93 (m, 1H; N(1")CH_2C<u>H</u>), 6.13 (d, *J*=7.6 Hz, 2H; o-Ph-H), 6.55 (d, *J*=7.6 Hz, 2H; o-Ph-H), 6.90 (t, *J*=7.6 Hz, 2H; m-Ph-H), 7.01-7.20 (m, 6H; Ph-H, 5"-H, 7"-H), 7.30-7.47 (m, 6H; Ph-H, 4"-H), 7.57 (t, *J*=7.7 Hz, 1H; 6"-H), 7.63 ppm (s, 1H; N(9)H); ^{13}C NMR (75 MHz, DMSO-d_6): δ=25.2, 25.9 (N(3)Me, N(1)Me), 34.9 (N(1")CH_2), 41.3 (N(1")CH_2), 50.4 (C-4'), 57.5 (C-5'), 68.9 (C-6), 79.2, 79.7, 82.9 (C-3a, C-9a, C-2'), 109.5 (C-7"), 117.0, 122.5, 122.8, 126.5, 126.7, 127.1, 127.6, 127.7, 128.1, 128.8, 129.6, 130.7, 131.4, 133.8, 134.6, 138.0 (Ph, HC=CH_2, C-3a", C-4", C-5", C-6"), 144.3 (C-7a"), 146.4 (C-4a), 159.1 (C-2), 167.0 (C-7), 174.2 ppm (C-2"); IR (KBr): ν=3414, 3293 (NH); 3060, 3026 (Ar), 2967, 2941, 2908 (Alk), 1716 (br), 1640 (C=O), 1609 cm^{-1} (C=N); HRMS (ESI): *m/z* calcd for $C_{40}H_{37}N_7O_3S+H^+$: 696.2751 [M+H$^+$]; found: 696.2751.

5''-Bromo-1,1',1'',3-tetramethyl-3a,4',9a-triphenyl-3,3a,9,9a-tetrahydrodispiro[imidazo[4,5-e]thiazolo[3,2-b]-1,2,4-triazine-6,3'-pyrrolidine-2',3''-indole]-2,2'',7(1H,1''H)-trione (4o): white solid (52% yield); m.p. 214-216 °C (from acetonitrile); ^{1}H NMR (300 MHz, DMSO-d_6): δ=2.16 (s, 3H; N(1')Me), 2.41 (s, 3H; N(1)Me), 2.64 (s, 3H; N(3)Me), 3.22 (s, 3H; N(1")Me), 3.63 (t, *J*=8.3 Hz, 1H; 5′-H), 4.01 (t, *J*=9.2 Hz, 1H; 5′-H), 4.50 (t, *J*=8.6 Hz, 1H; 4'-H), 6.07 (d, *J*=7.3 Hz, 2H; o-Ph-H), 6.66 (d, *J*=7.4 Hz, 2H; o-Ph-H), 6.94-7.16 (m, 6H; Ph-H), 7.27-7.54 (m, 7H; Ph-H, 4"-H, 7"-H), 7.90 (d, *J*=8.1 Hz, 1H; 6"-H), 8.28 ppm (s, 1H; N(9)H); ^{13}C NMR (75 MHz, DMSO-d_6): δ=25.2, 25.9, 25.9 (N(3)Me, N(1)Me, N(1")Me), 34.9 (N(1')Me), 50.1 (C-4'), 57.7 (C-5'), 68.8 (C-6), 79.1, 79.8, 82.2 (C-3a, C-9a, C-2'), 110.9 (C-7"), 115.1, 124.5, 127.0, 127.1, 127.5, 127.6, 127.8, 128.0, 128.8, 129.7, 133.7, 133.9, 134.6, 137.9 (Ph, C-3a", C-4", C-5", C-6"), 144.54 (C-7a"), 146.01 (C-4a), 159.1 (C-2), 166.7 (C-7), 173.9 ppm (C-2"); IR (KBr): ν=3435, 3168 (NH), 3062, 3030 (Ar), 2943, 2873 (Alk), 1715 (br), 1642 (C=O), 1607 cm^{-1} (C=N); HRMS (ESI): *m/z* calcd for $C_{38}H_{34}BrN_7O_3S+Na^+$: 770.1519 [M+Na$^+$]; found: 770.1505.

1''-Allyl-1,1',3-trimethyl-4'-(4-nitrophenyl)-3,3a,9,9a-tetrahydrodispiro[imidazo[4,5-e]thiazolo[3,2-b]-1,2,4-triazine-6,3'-pyrrolidine-2',3''-indole]-2,2'',7(1H,1''H)-trione (4p): yellowish solid (65% yield); m.p. 238-240 °C (from acetonitrile); ^{1}H NMR (300 MHz, DMSO-d_6): δ=2.06 (s, 3H; N(1')Me), 2.53 (s, 3H; NMe), 2.63 (s, 3H; NMe), 3.54 (t, *J*=8.3 Hz, 1H; 5'-H), 3.95 (t, *J*=9.2 Hz, 1H; 5'-H), 4.22-4.43 (m, 2H; N(1")CH_2), 4.46-4.56 (m, 3H; 4'-H, 3a-H, 9a-H), 5.16-5.22 (m, 2H; CH=CH_2), 5.77-5.86 (m, 1H; N(1")CH_2C<u>H</u>), 6.67 (d, *J*=1.9 Hz, 1H; NH), 6.99 (d, *J*=7.8 Hz, 1H; 7"-H), 7.13 (t, *J*=7.6 Hz, 1H; 5"-H), 7.38-7.44 (m, 2H; 4"-H, 6"-H), 7.55 (d, *J*=8.5 Hz, 2H; o-Ar-H), 8.19 ppm (d, *J*=8.5 Hz, 2H; m-Ar-H); ^{13}C NMR (75 MHz, DMSO-d_6): δ=26.7, 27.5, 34.7 (NMe), 41.3 (N(1")CH_2), 52.1 (C-4'), 56.4 (C-5'), 65.1, 66.0, 66.8 (C-3a, C-9a, C-6), 78.8 (C-2'), 109.6 (C-7"), 117.2, 122.5, 123.3, 123.6, 127.0, 130.6, 130.8, 131.4 (Ar, HC=CH_2, C-3a", C-4", C-5", C-6"), 144.1 (C-7a"), 145.3, 146.9, 147.2 (C-4a, ipso-Ar, CNO_2), 158.5 (C-2), 168.5 (C-7), 174.1 ppm (C-2"); IR (KBr) ν=3464, 3289 (NH), 3080, 3059 (Ar), 2963, 2951 (Alk), 1714, 1699, 1644 (C=O), 1610 (C=N), 1511, 1344 cm^{-1} (NO_2); HRMS (ESI): *m/z* calcd for $C_{28}H_{28}N_8O_5S+H^+$: 589.1976 [M+H$^+$]; found: 589.1974.

5''-Bromo-1''-ethyl-1,1',3-trimethyl-4'-(4-nitrophenyl)-3,3a,9,9a-tetrahydrodispiro[imidazo[4,5-e]thiazolo[3,2-b]-1,2,4-triazine-6,3'-pyrrolidine-2',3''-indole]-2,2'',7(1H,1''H)-trione (4q): yellowish solid (62% yield); m.p. 250-252 °C (from acetonitrile); ^{1}H NMR (300 MHz, DMSO-d_6): δ=1.11 (t, *J*=7.0 Hz, 3H; Me), 2.06 (s, 3H; N(1')Me), 2.52 (s, 3H; NMe), 2.65 (s, 3H; NMe), 3.53 (t, *J*=8.2 Hz, 1H; 5'-H), 3.64-3.80 (m, 2H; N(1")CH_2), 3.93 (t, *J*=9.2 Hz, 1H; 5'-H), 4.52-4.59 (m, 3H; 4'-H, 3a-H, 9a-H), 6.84 (s, 1H; NH), 7.13 (d, *J*=8.3 Hz, 1H; 7"-H), 7.51 (d, *J*=8.4 Hz, 2H, o-Ar-H), 7.55 (s, 1H; 4"-H), 7.62 (d, *J*=8.3 Hz, 1H; 6"-H), 8.19 ppm (d, *J*=8.4 Hz, 2H; m-Ar-H); ^{13}C NMR (75 MHz, DMSO-d_6): δ=12.4 (Me), 26.7, 27.6 (NMe), 34.2 (N(1")CH_2), 34.7 (NMe), 52.6 (C-4'), 56.2 (C-5'), 65.2, 66.2, 67.0 (C-3a, C-9a, C-6), 78.3 (C-2'), 111.1 (C-7"), 115.0, 123.6, 125.2, 130.2, 130.7, 133.5 (Ar, C-3a", C-4", C-5", C-6"), 143.3, 144.9, 146.9, 148.1 (C-4a, C-7a", ipso-Ar, CNO_2), 158.6 (C-2), 168.4 (C-7), 173.6 ppm (C-2"); IR (KBr) ν=3462, 3185 (NH), 3075 (Ar), 2978, 2949 (Alk), 1728, 1686, 1642 (C=O), 1606 (C=N), 1518, 1345 cm^{-1} (NO_2); HRMS (ESI): *m/z* calcd for $C_{27}H_{27}BrN_8O_5S+Na^+$: 677.0901 [M+Na$^+$]; found: 677.0899.

1''-Allyl-1,3-diethyl-1'-methyl-4'-(4-nitrophenyl)-3,3a,9,9a-tetrahydrodispiro[imidazo[4,5-e]thiazolo[3,2-b]-1,2,4-triazine-6,3'-pyrrolidine-2',3''-indole]-2,2'',7(1H,1''H)-trione (4r): yellowish solid (71% yield); m.p. 166-168 °C (from acetonitrile); ^{1}H NMR (300 MHz, DMSO-d_6): δ=0.93 (t, *J*=7.2 Hz, 3H; Me), 0.99 (t, *J*=7.0 Hz, 3H; Me), 2.05 (s, 3H; N(1')Me), 2.80-2.87 (m, 1H; NCH_2), 3.07-3.19 (m, 3H; NCH_2), 3.55 (t, *J*=8.2 Hz, 1H; 5'-H), 3.93 (t, *J*=9.4 Hz, 1H; 5'-H), 4.22-4.43 (m, 2H; N(1")CH_2), 4.50 (t, *J*=8.7 Hz, 1H; 4'-H), 4.58-4.63 (m, 2H; 3a-H, 9a-H), 5.16-5.22 (m, 2H; CH=C<u>H</u>$_2$), 5.77-5.88 (m, 1H; N(1")CH_2C<u>H</u>), 6.72 (s, 1H; NH), 7.00 (d, *J*=7.8 Hz, 1H; 7"-H), 7.13 (t, *J*=7.5 Hz, 1H; 5"-H), 7.38-7.43 (m, 2H; 4"-H, 6"-H), 7.56 (d, *J*=8.3 Hz, 2H; o-Ar-H), 8.20 ppm (d, *J*=8.3 Hz, 2H; m-Ar-H); ^{13}C NMR (75 MHz, DMSO-d_6): δ=13.2, 13.4 (Me), 34.2 (NCH_2), 34.7 (NMe), 35.2 (NCH_2), 41.3 (N(1")CH_2), 52.2 (C-4'), 56.7 (C-5'), 63.1, 64.6 (C-3a, C-9a), 66.7 (C-6), 78.9 (C-2'), 109.7 (C-7"), 117.2, 122.5, 123.3, 123.6, 127.0, 130.7, 131.0, 131.4 (Ar, HC=CH2, C-3a", C-4", C-5", C-6"), 144.1, 145.3, 146.9, 148.0 (C-4a, C-

7a", ipso-Ar, CNO_2), 157.7 (C-2), 168.6 (C-7), 174.1 ppm (C-2"); IR (KBr) ν=3448, 3237 (NH), 3045 (Ar), 2977, 2942 (Alk), 1697, 1680, 1634 (C=O), 1608 (C=N), 1524, 1350 cm^{-1} (NO_2); HRMS (ESI): *m/z* calcd for $C_{30}H_{32}N_8O_5S+H^+$: 617.2289 [M+H$^+$]; found: 617.2283.

5''-Bromo-1,1'',3-triethyl-1'-methyl-4'-(4-nitrophenyl)-3,3a,9,9a-tetrahydrodispiro[imidazo[4,5-e]thiazolo[3,2-b]-1,2,4-triazine-6,3'-pyrrolidine-2',3''-indole]-2,2'',7(1H,1''H)-trione (4s): beige solid (55% yield); m.p. 130-132 °C (from acetonitrile); ^{1}H NMR (300 MHz, DMSO-d_6): δ=0.93 (t, *J*=6.8 Hz, 3H; Me), 1.00 (t, *J*=6.6 Hz, 3H; Me), 1.12 (t, *J*=6.5 Hz, 3H; Me), 2.05 (s, 3H; N(1')Me), 2.82-2.87 (m, 1H; NCH_2), 3.10-3.20 (m, 3H; NCH_2), 3.54 (t, *J*=8.8 Hz, 1H; 5'-H), 3.63-3.81 (m, 2H; N(1")CH_2), 3.91 (t, *J*=9.2 Hz, 1H; 5'-H), 4.60 (t, *J*=8.4 Hz, 1H; 4'-H), 4.64 (br s, 2H; 3a-H, 9a-H), 6.87 (s, 1H; NH), 7.13 (d, *J*=8.3 Hz, 1H; 7"-H), 7.52 (d, *J*=8.1 Hz, 2H, o-Ar-H), 7.54 (s, 1H; 4"-H), 7.62 (d, *J*=8.3 Hz, 1H; 6"-H), 8.20 ppm (d, *J*=8.1 Hz, 2H; m-Ar-H); ^{13}C NMR (75 MHz, DMSO-d_6): δ=12.7, 13.4, 13.6 (Me), 34.5, 34.6 (NCH_2), 34.9 (NMe), 35.5 (NCH_2), 52.9 (C-4'), 56.7 (C-5'), 63.5, 65.0, 67.2 (C-3a, C-9a, C-6), 78.6 (C-2'), 111.4 (C-7"), 115.3, 123.9, 125.4, 130.4, 131.1, 133.9 (Ar, C-3a", C-4", C-5", C-6"), 143.6, 145.2, 147.2, 148.3 (C-4a, C-7a", ipso-Ar, CNO_2), 158.1 (C-2), 168.7 (C-7), 173.9 ppm (C-2"); IR (KBr) ν=3441, 3282 (NH), 3076 (Ar), 2976, 2936 (Alk), 1698 (broad), 1649 (C=O), 1605 (C=N), 1514, 1345 cm^{-1} (NO_2); HRMS (ESI): *m/z* calcd for $C_{29}H_{31}BrN_8O_5S+H^+$: 683.1394 [M+H$^+$]; found: 683.1391.

4'-(2,4-Dichlorophenyl)-1,3-diethyl-1'-methyl-3,3a,9,9a-tetrahydrodispiro[imidazo[4,5-e]thiazolo[3,2-b]-1,2,4-triazine-6,3'-pyrrolidine-2',3''-indole]-2,2'',7(1H,1''H)-trione (4t): white solid (74% yield); m.p. 214-216 °C (from acetonitrile); ^{1}H NMR (300 MHz, DMSO-d_6): δ=0.95 (t, *J*=7.0 Hz, 3H; Me), 0.99 (t, *J*=7.1 Hz, 3H; Me), 2.07 (s, 3H; N(1')Me), 2.87-2.96 (m, 1H; NCH_2), 2.98-3.13 (m, 2H; NCH_2), 3.16-3.28 (m, 1H; NCH_2), 3.49 (t, *J*=8.5 Hz, 1H; 5'-H), 3.94 (t, *J*=9.1 Hz, 1H; 5'-H), 4.53 (dd, *J*= 5.9 Hz, *J*=2.3 Hz, 1H; 9a-H), 4.61 (d, *J*=5.9 Hz, 1H; 3a-H), 4.70 (t, *J*=8.6 Hz, 1H; 4'-H), 6.47 (d, *J*=2.3 Hz, 1H; N(9)H), 6.83 (d, *J*=7.7 Hz, 1H; 7"-H), 7.03 (t, *J*=7.5 Hz, 1H, 5"-H), 7.19 (d, *J*=7.5 Hz, 1H, 4"-H), 7.31 (t, *J*=7.6 Hz, 1H, 6"-H), 7.51 (dd, ^3J=8.5 Hz, ^4J=1.9 Hz, 1H; Ar-H), 7.58 (d, *J*=1.9 Hz, 1H; Ar-H), 7.86 (d, *J*=8.5 Hz, 1H; Ar-H), 10.74 ppm (s, 1H; N(1'')H); ^{13}C NMR (75 MHz, DMSO-d_6): δ=12.5, 13.2 (Me), 34.0, 34.9 (NCH_2), 35.0 (NMe), 45.4 (C-4'), 55.2 (C-5'), 62.7, 63.8, 65.1 (C-3a, C-9a, C-6), 79.6 (C-2'), 110.2 (C-7"), 122.7, 122.8, 126.0, 127.4, 128.6, 130.7, 131.9, 132.9, 134.8, 136.7 (Ar, C-3a", C-4", C-5", C-6"), 143.8, 148.6 (C-4a, C-7a"), 157.6 (C-2), 168.6 (C-7), 176.0 ppm (C-2"); IR (KBr) ν=3412, 3271 (NH), 3096 (Ar), 2974, 2934 (Alk), 1718, 1689, 1640 (C=O), 1611 cm^{-1} (C=N); HRMS (ESI): *m/z* calcd for $C_{27}H_{27}Cl_2N_7O_3S+Na^+$: 622.1165 [M+Na$^+$]; found: 622.1151.

^{1}H NMR spectrum of **1d**

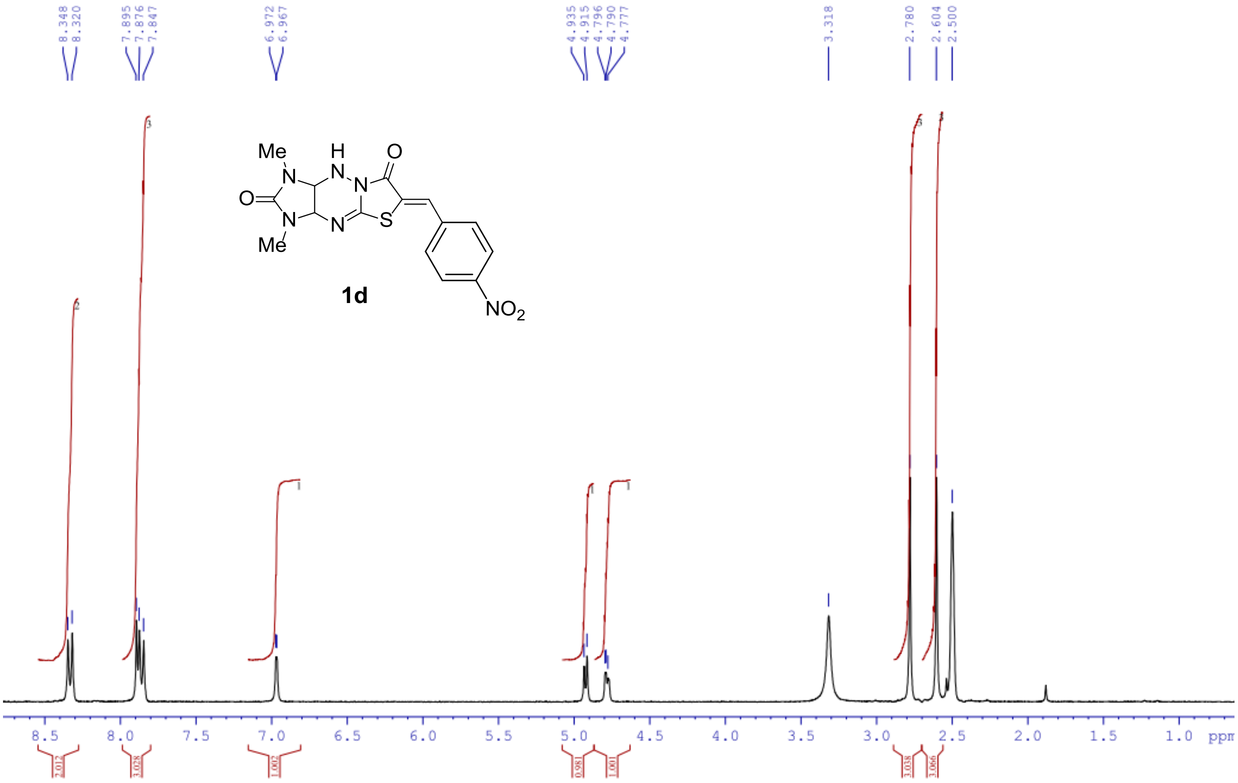

^{13}C NMR spectrum of **1d**

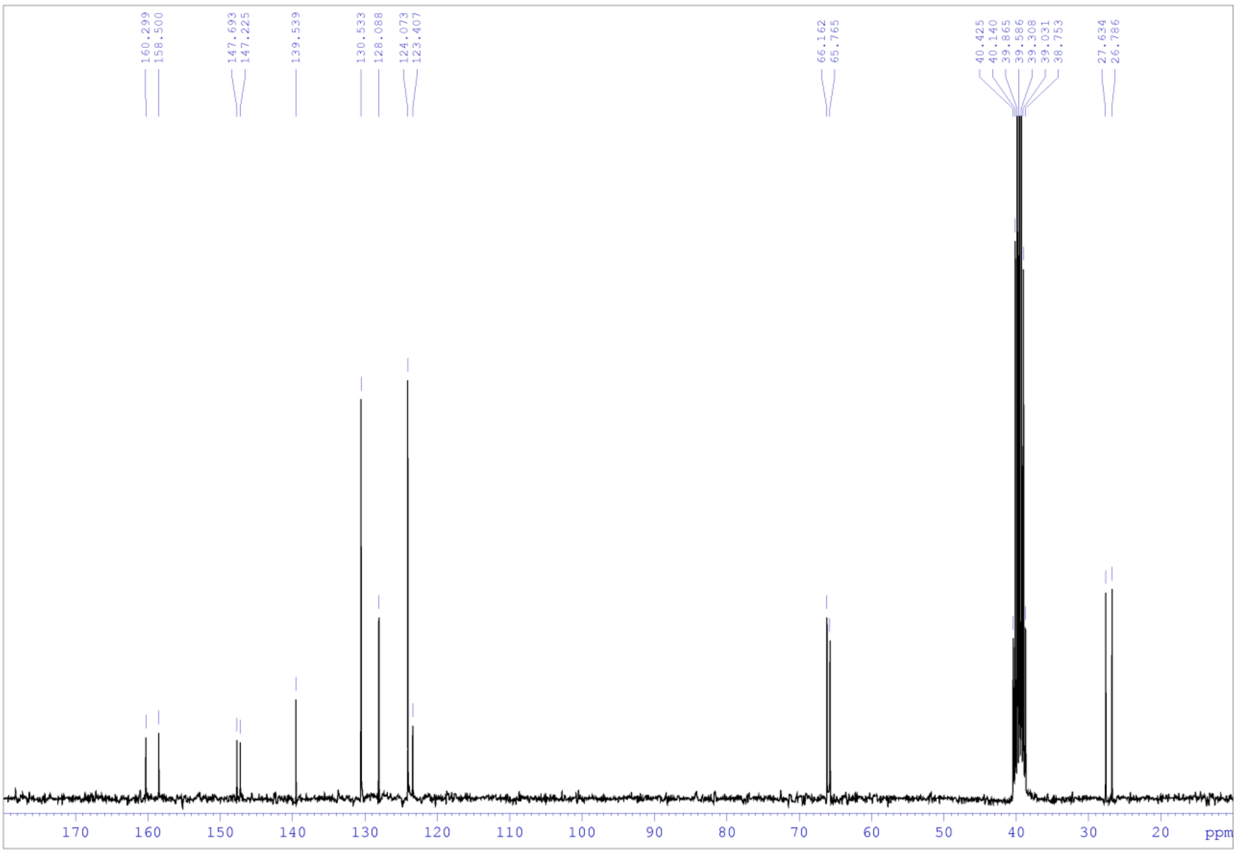

^{1}H NMR spectrum of **1e**

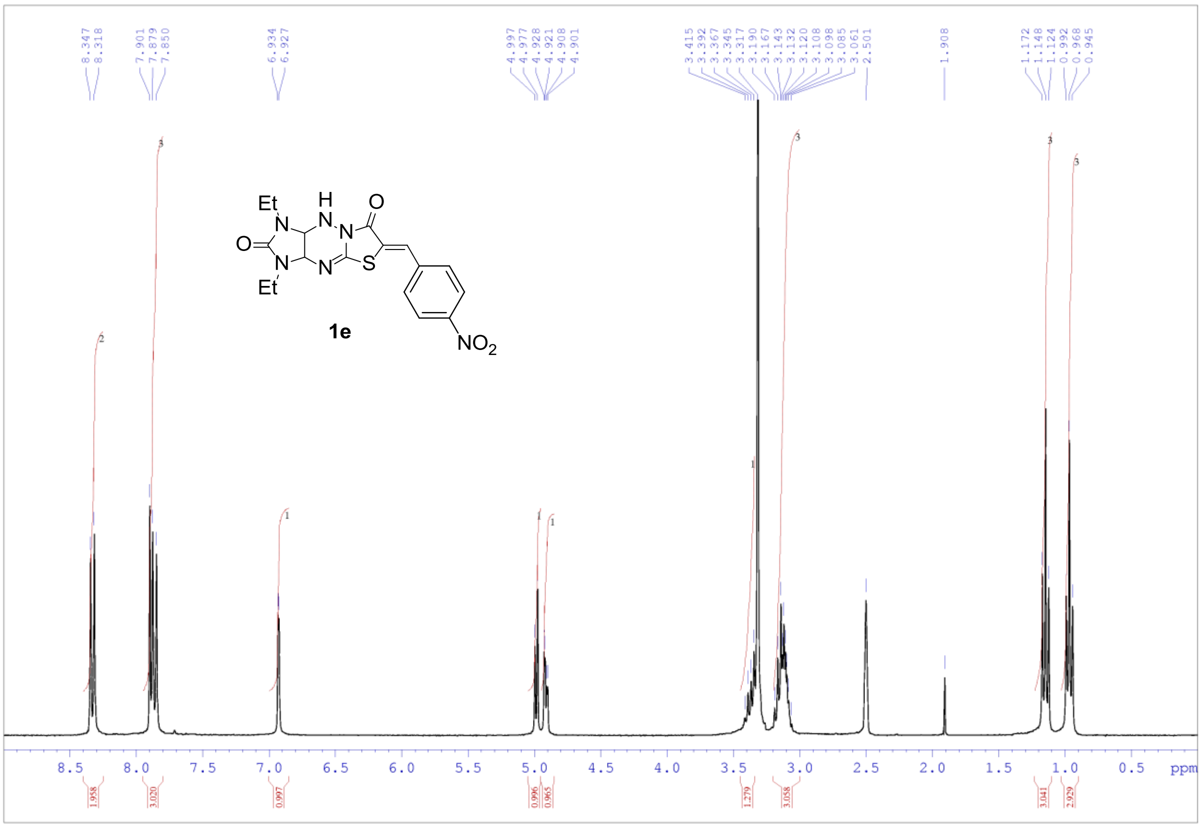

^{13}C NMR spectrum of **1e**

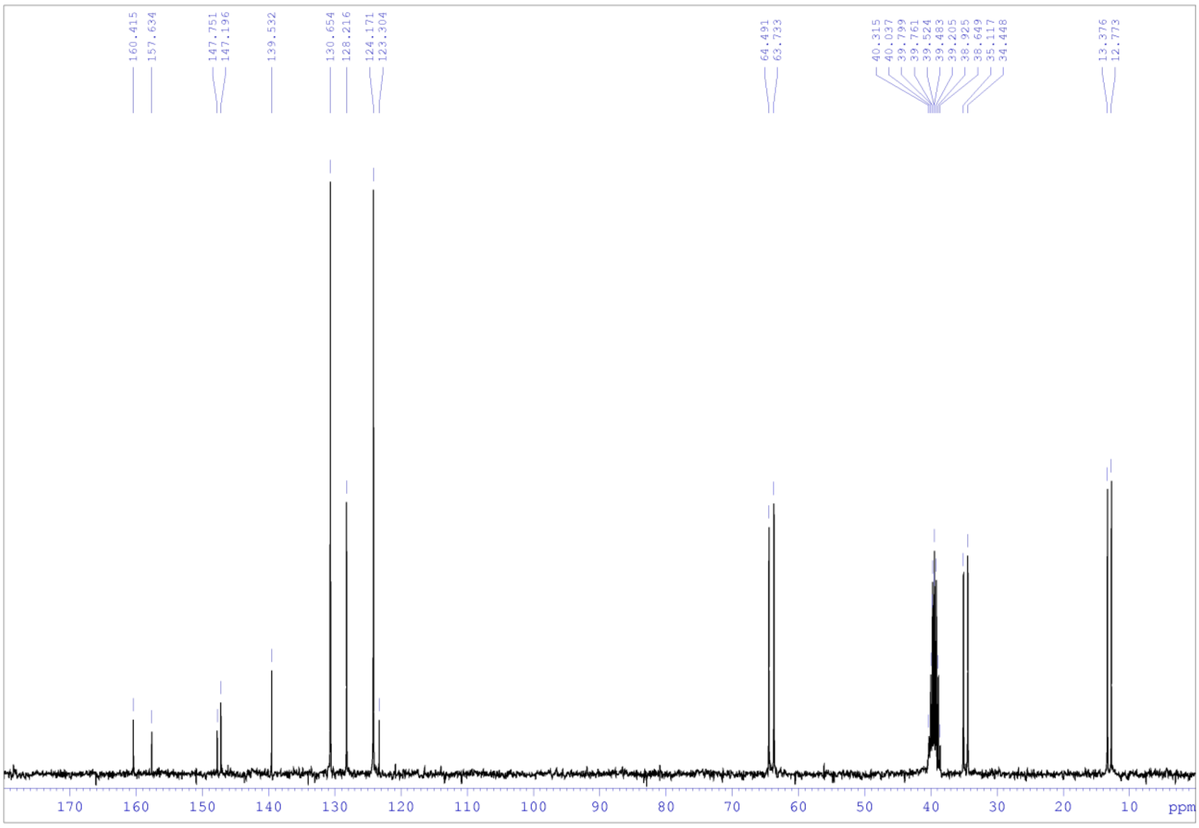

^{1}H NMR spectrum of **1f**

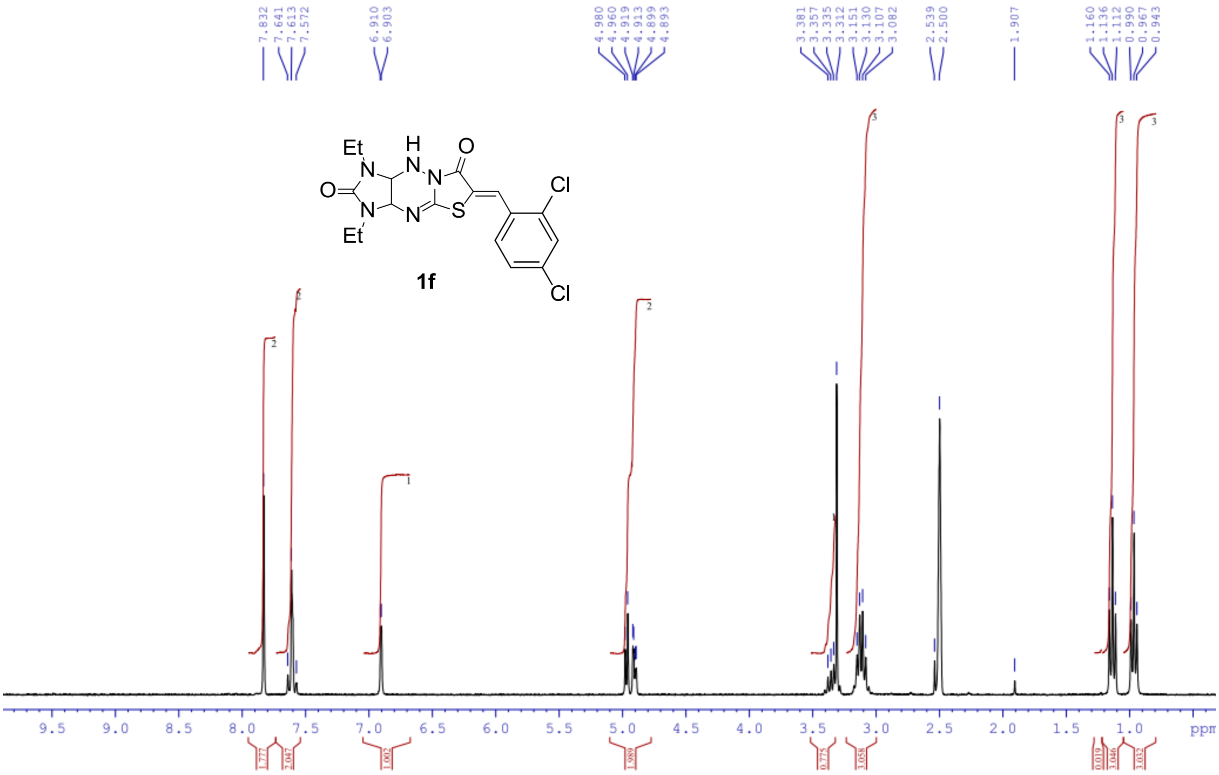

^{13}C NMR spectrum of **1f**

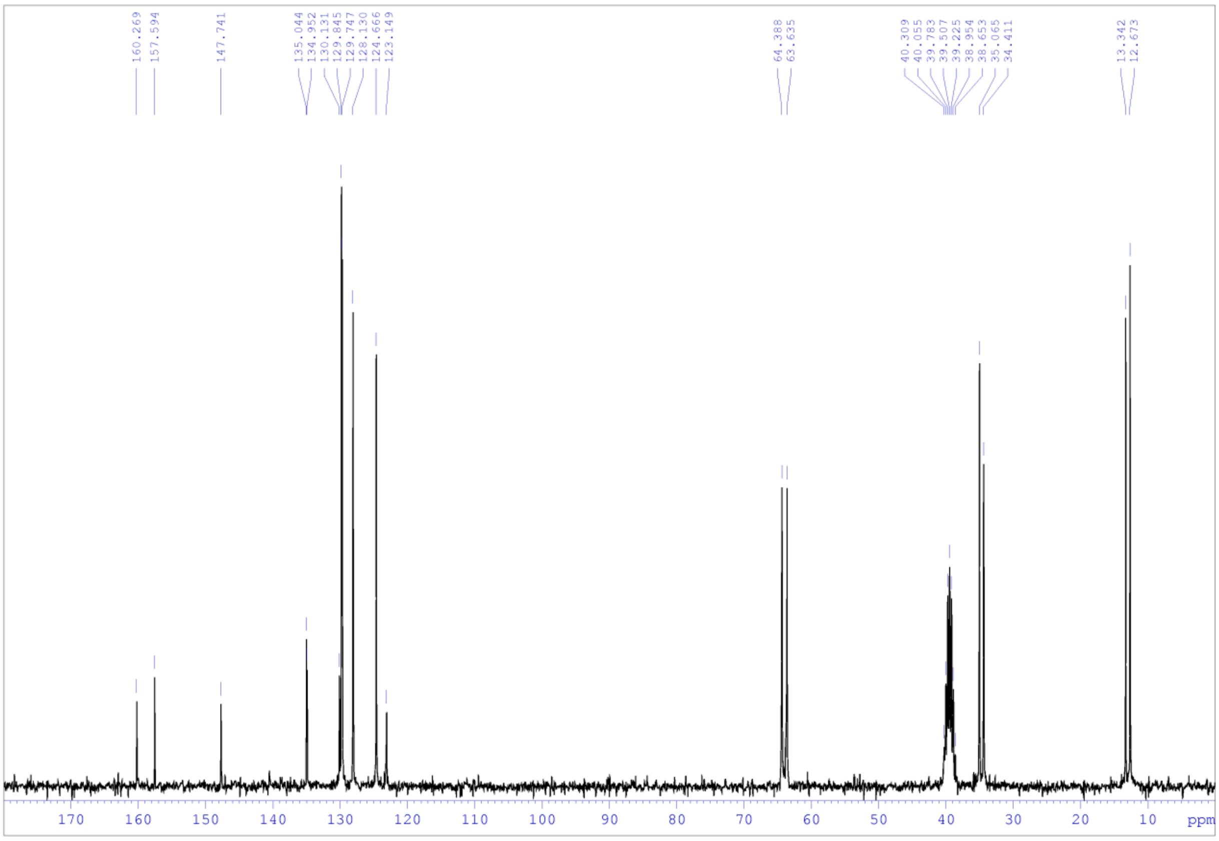

^{1}H NMR spectrum of **4a**

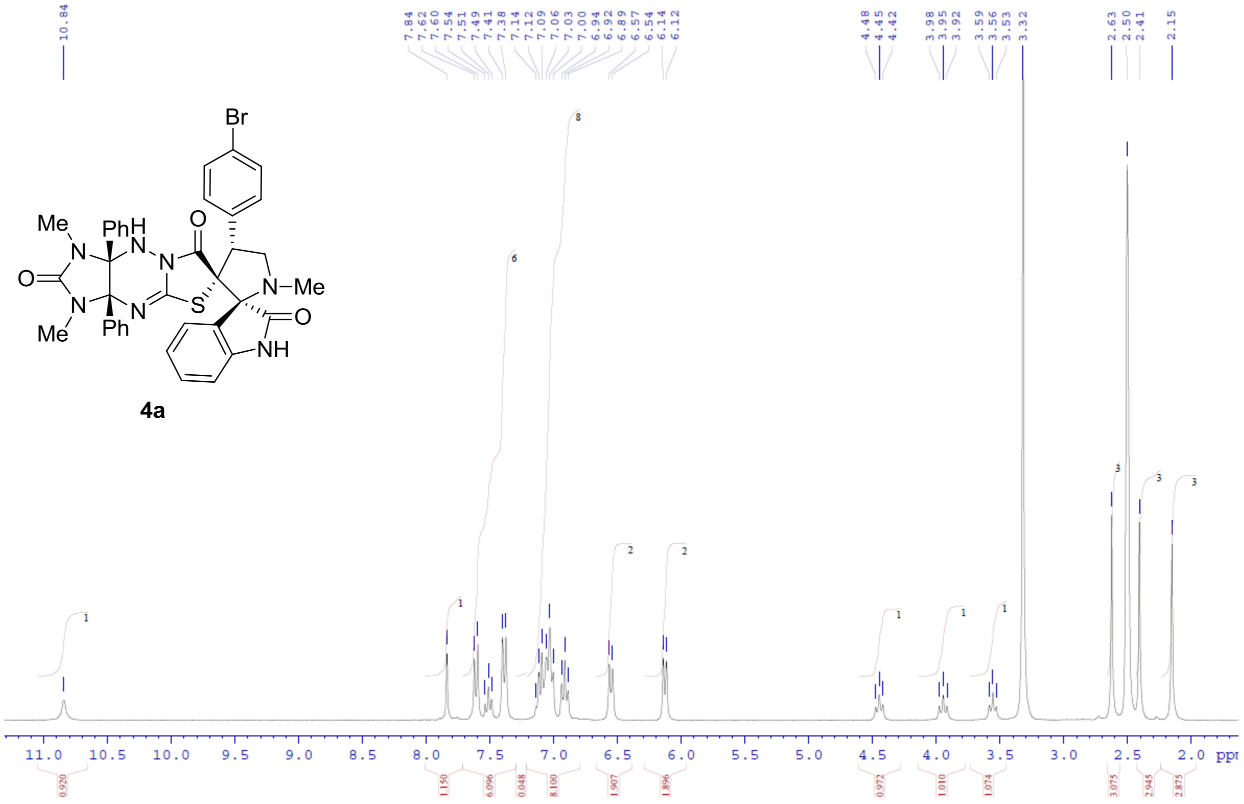

^{13}C NMR spectrum of **4a**

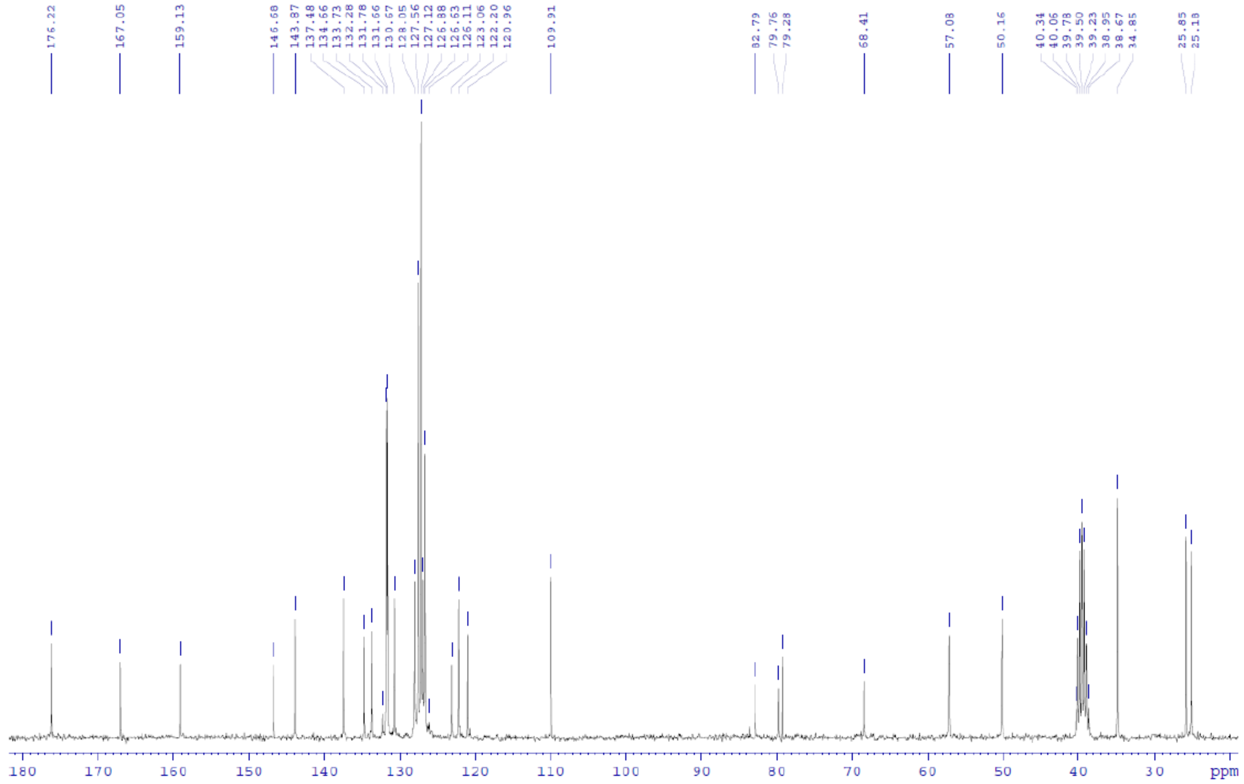

^{1}H NMR spectrum of **4b**

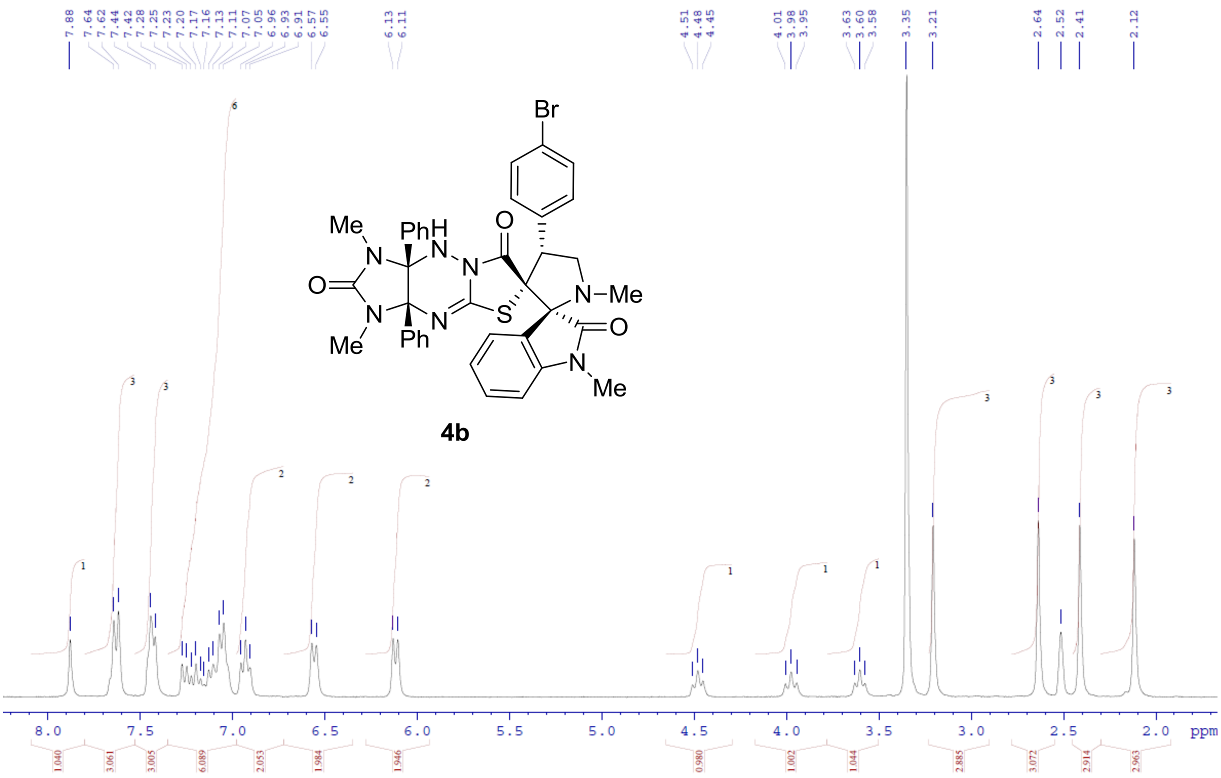

^{13}C NMR spectrum of **4b**

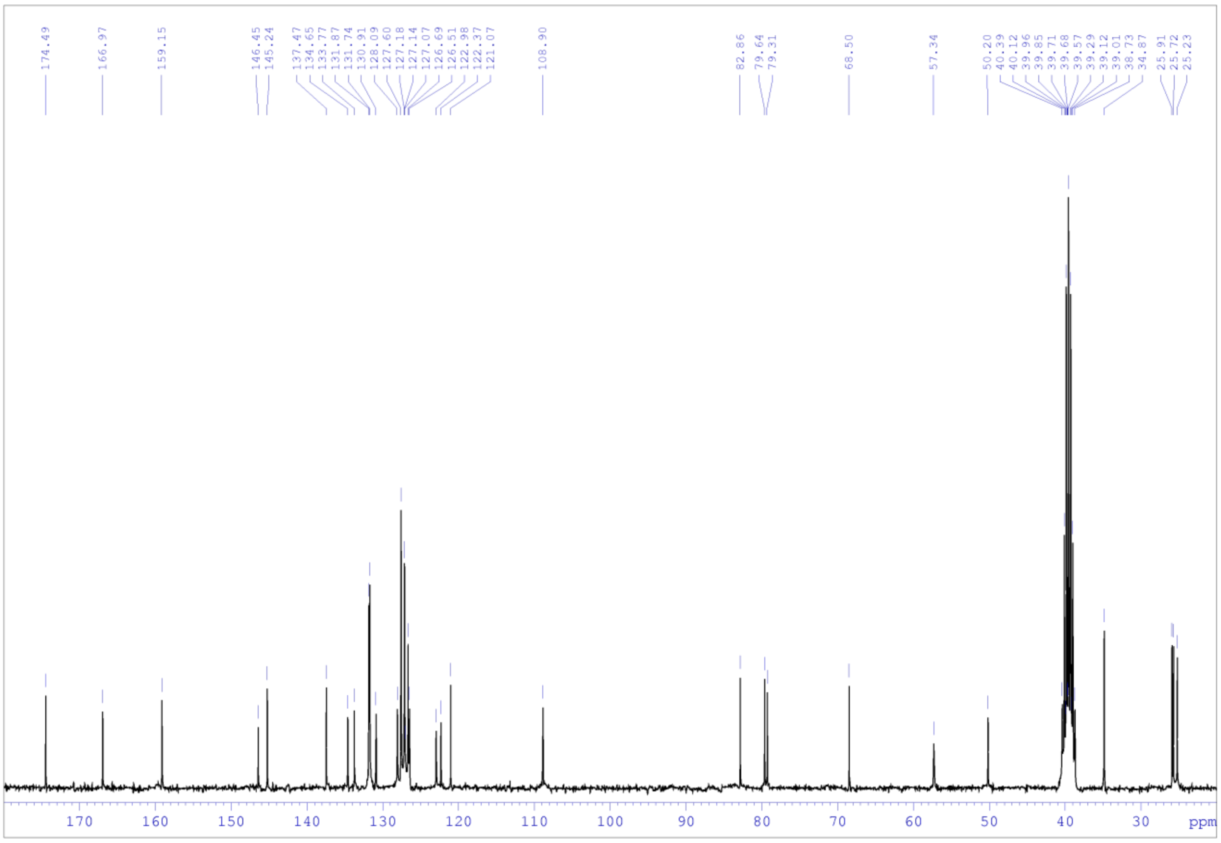

^{1}H NMR spectrum of **4c**

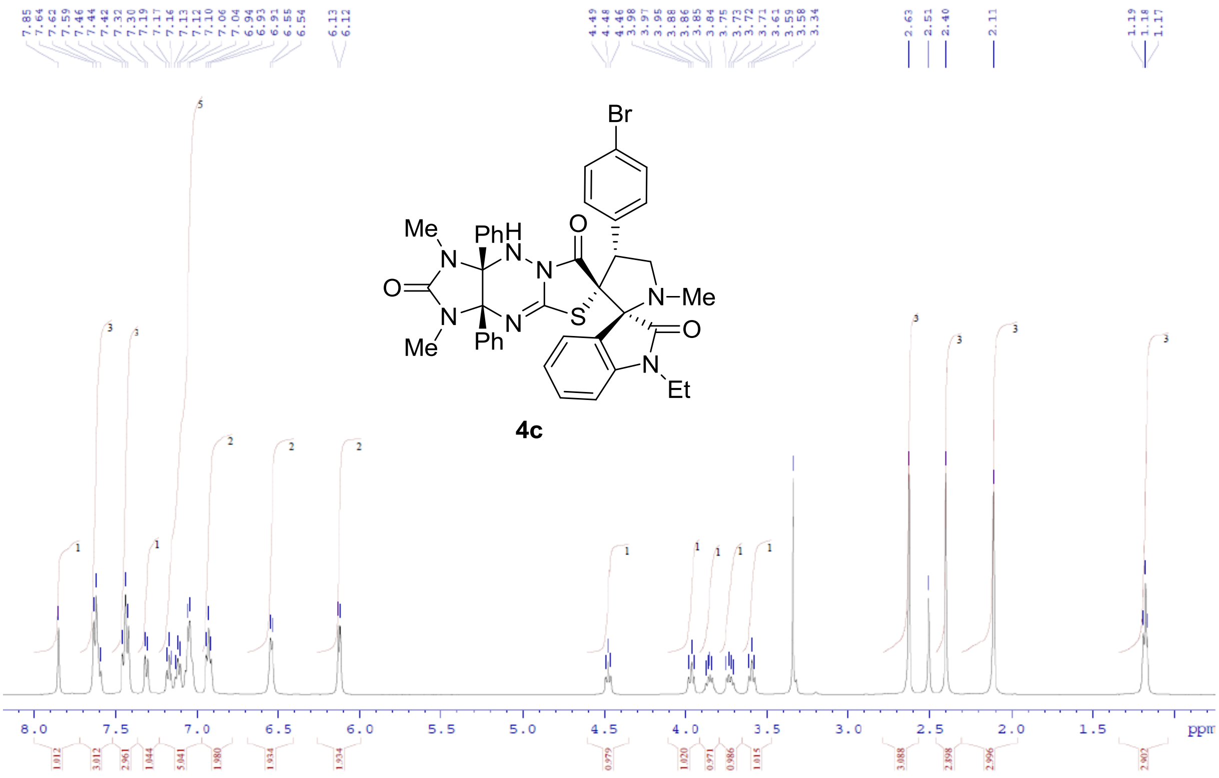

^{13}C NMR spectrum of **4c**

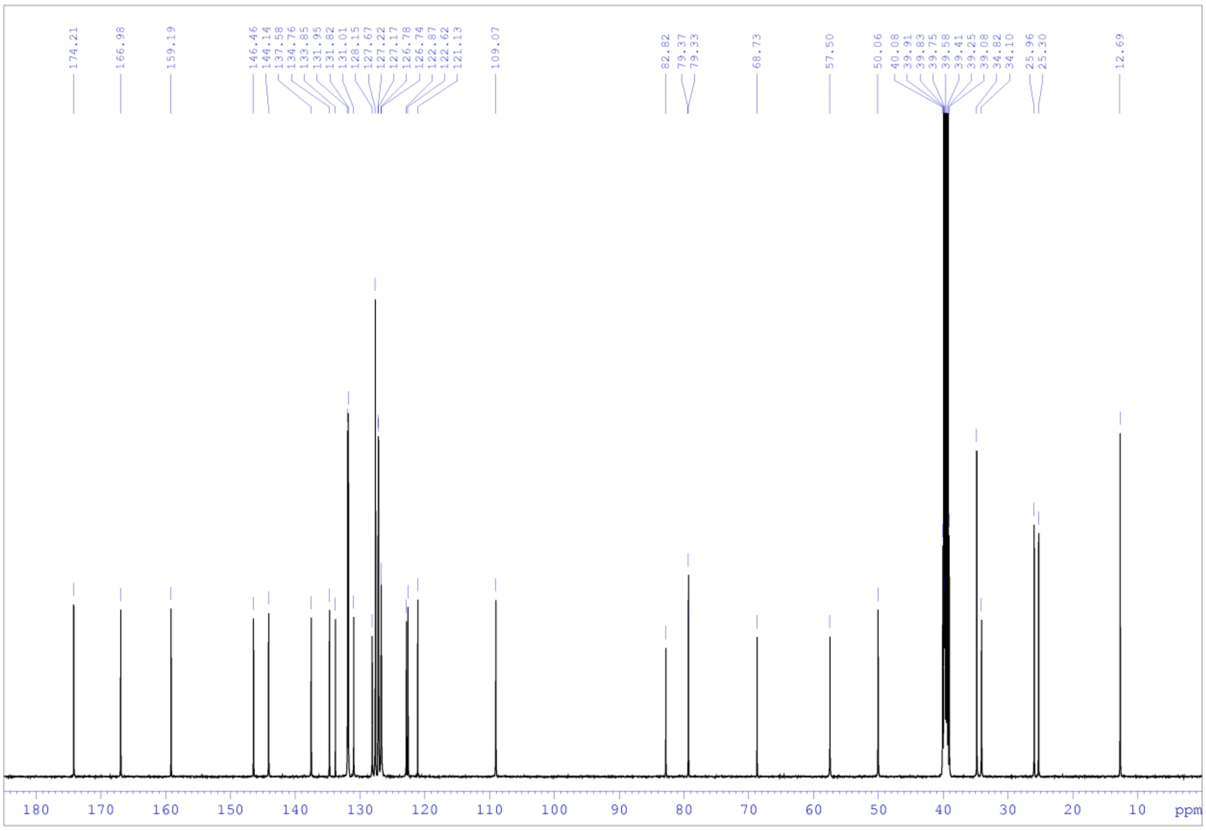

^{1}H NMR spectrum of **4d**

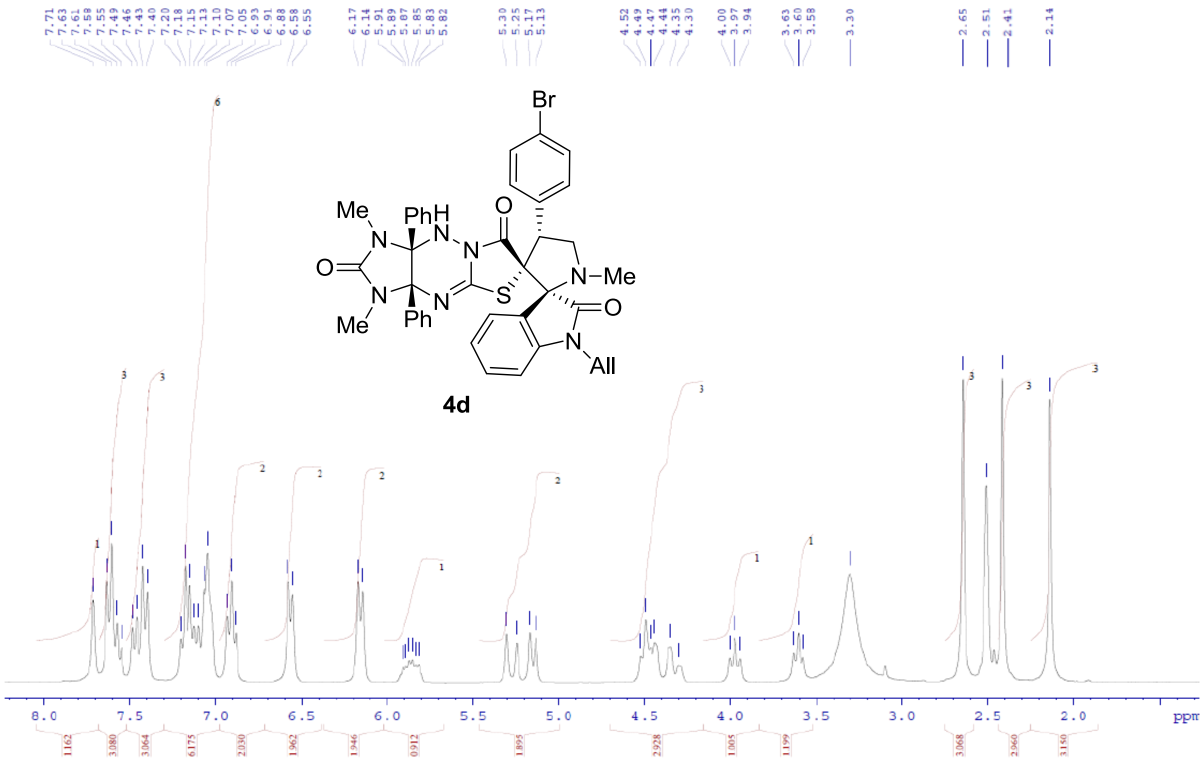

^{13}C NMR spectrum of **4d**

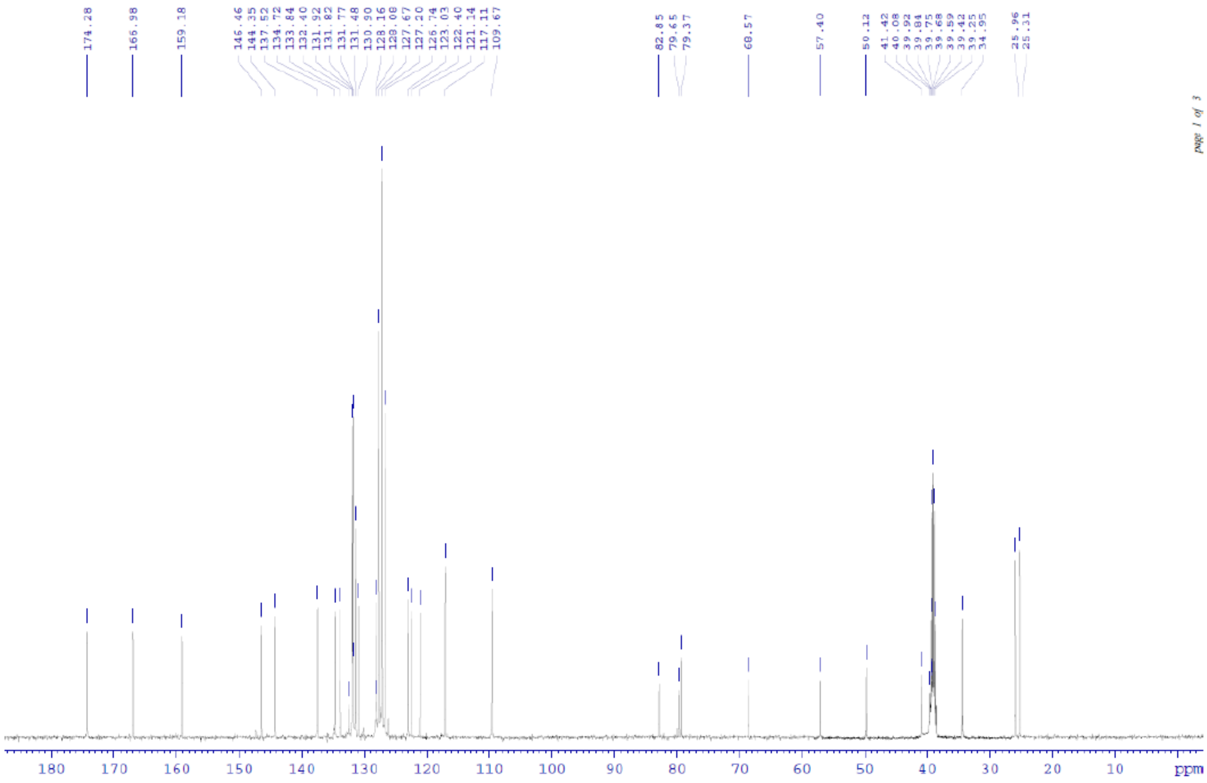

^{1}H NMR spectrum of **4e**

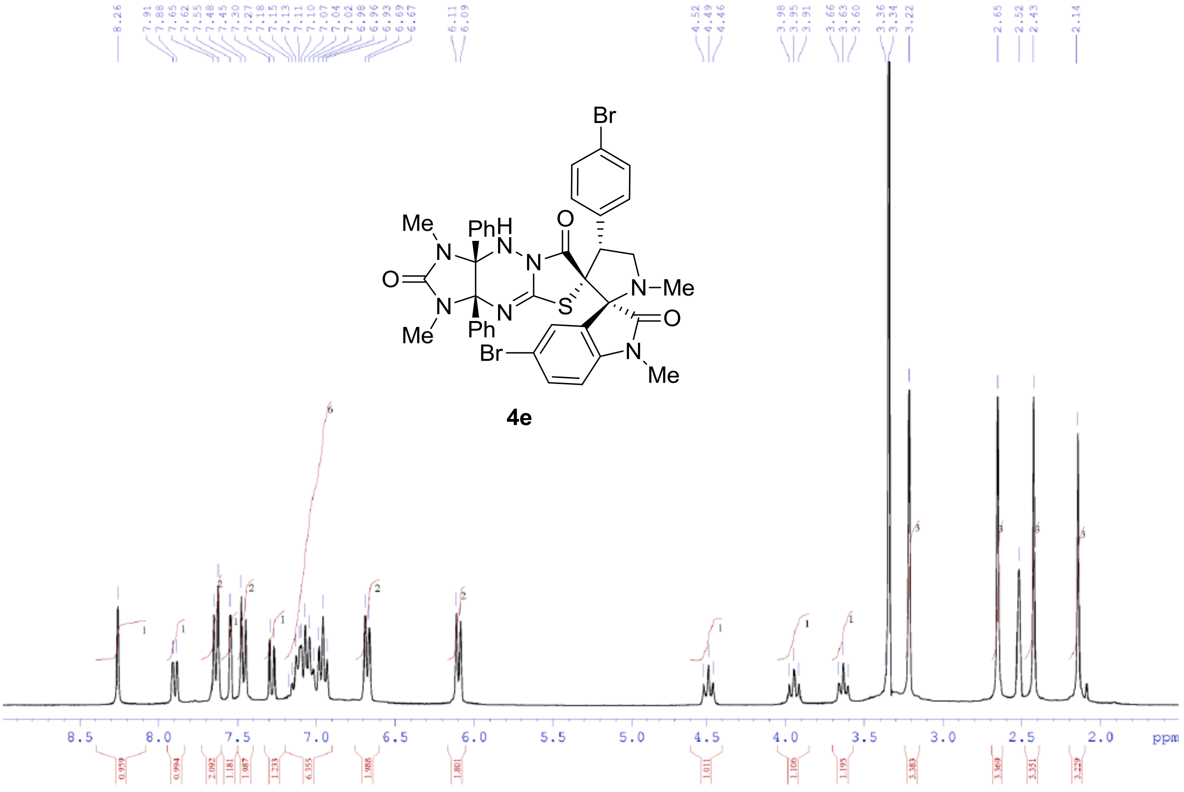

^{13}C NMR spectrum of **4e**

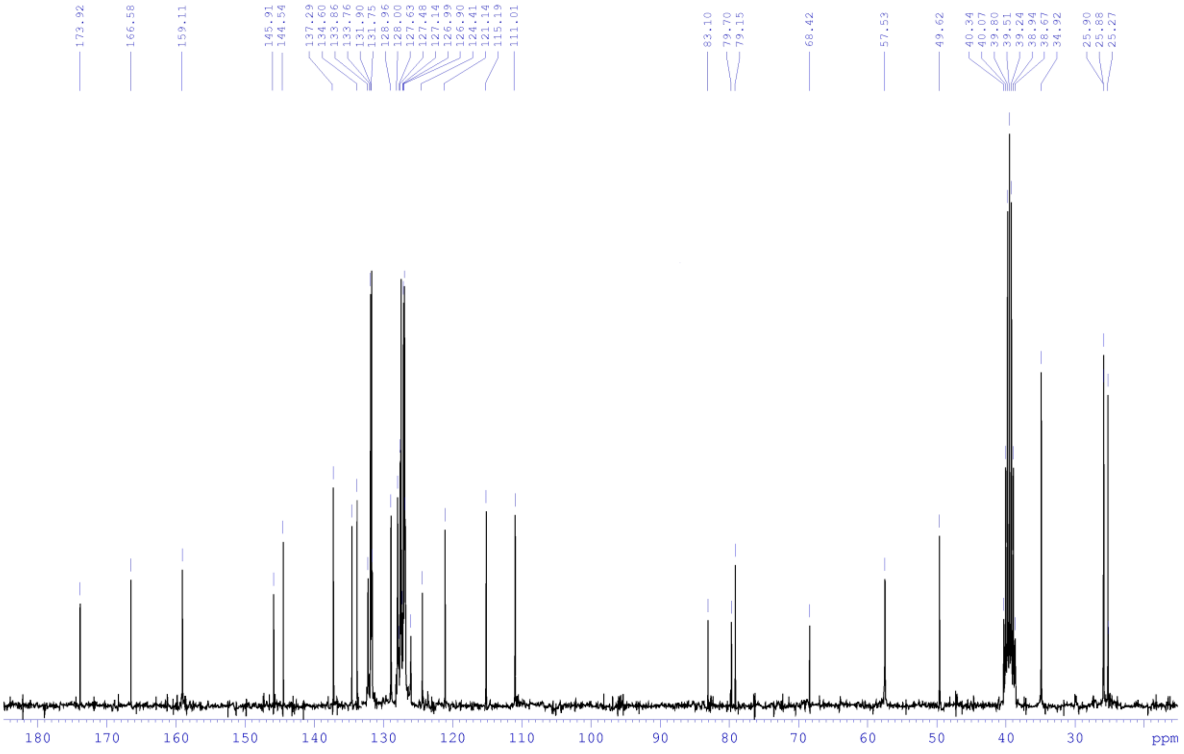

^{1}H NMR spectrum of **4f**

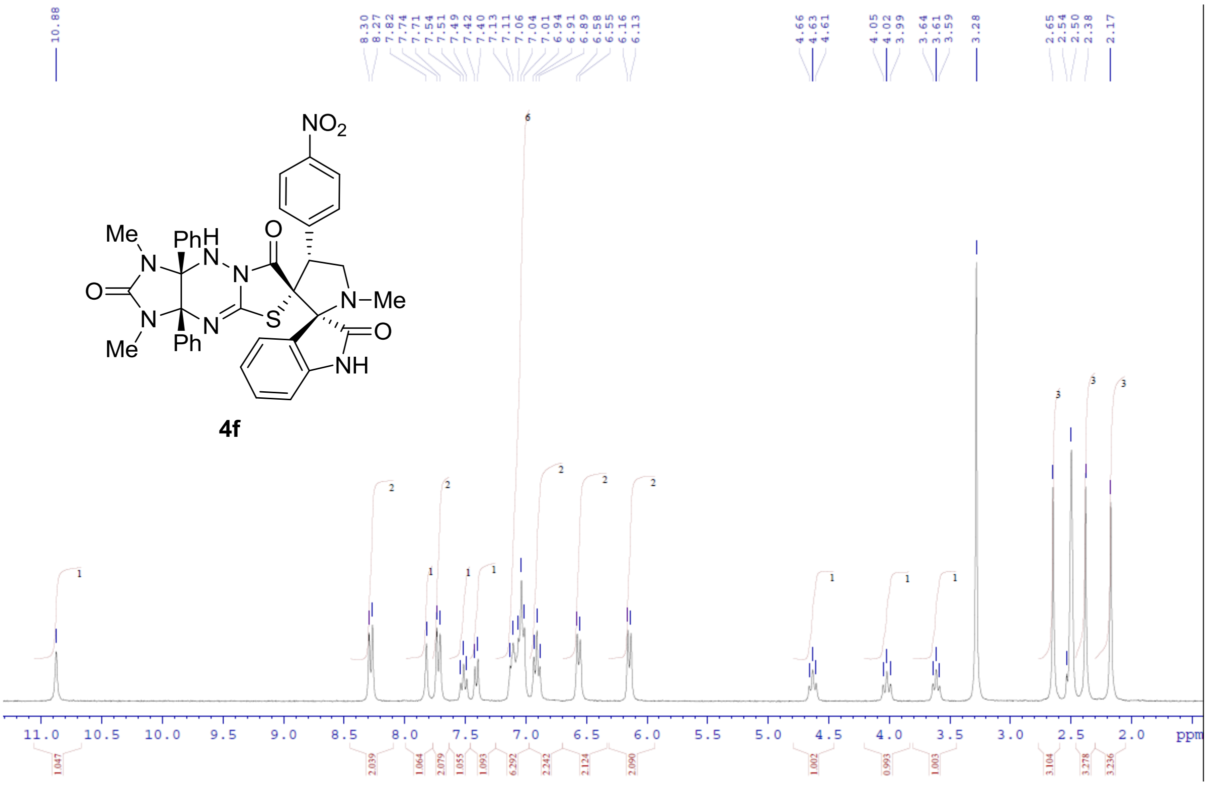

^{13}C NMR spectrum of **4f**

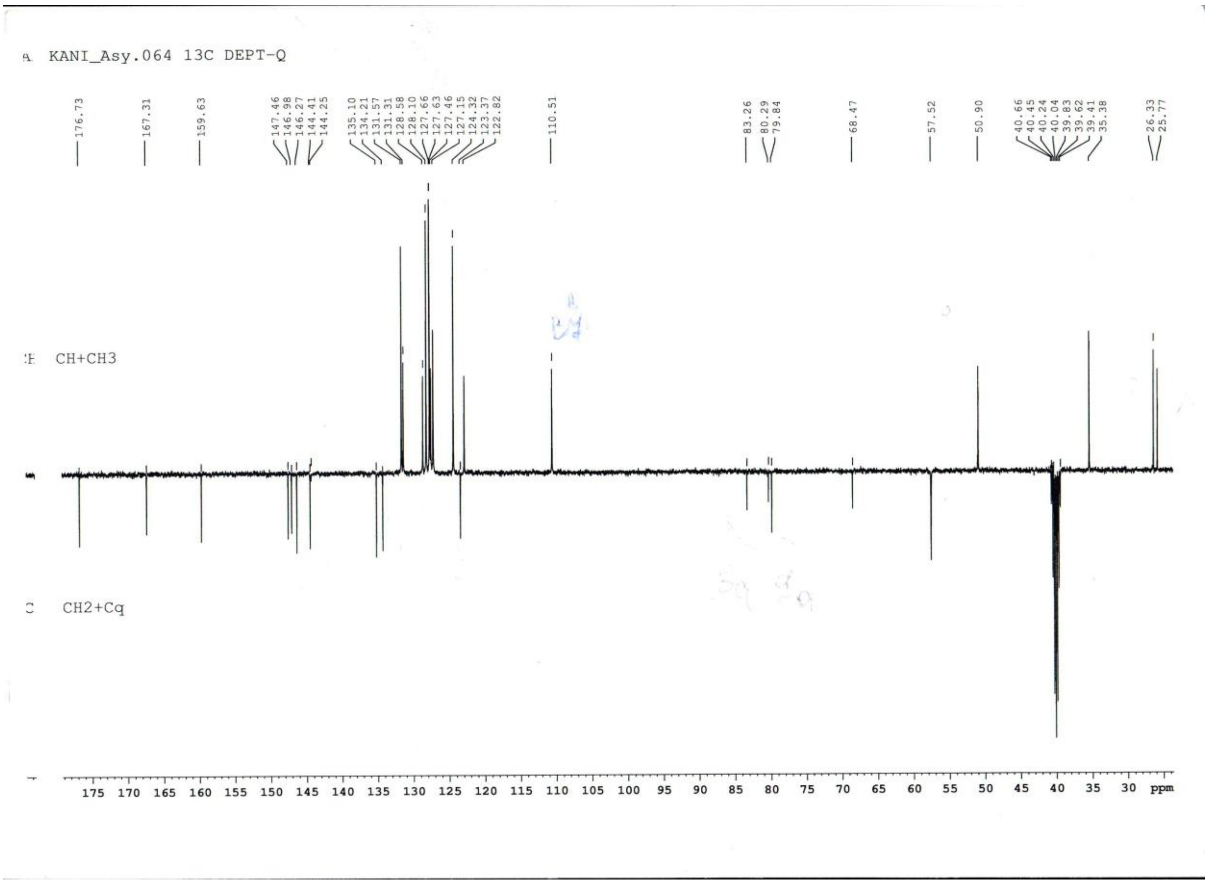

COSY spectrum of **4f**

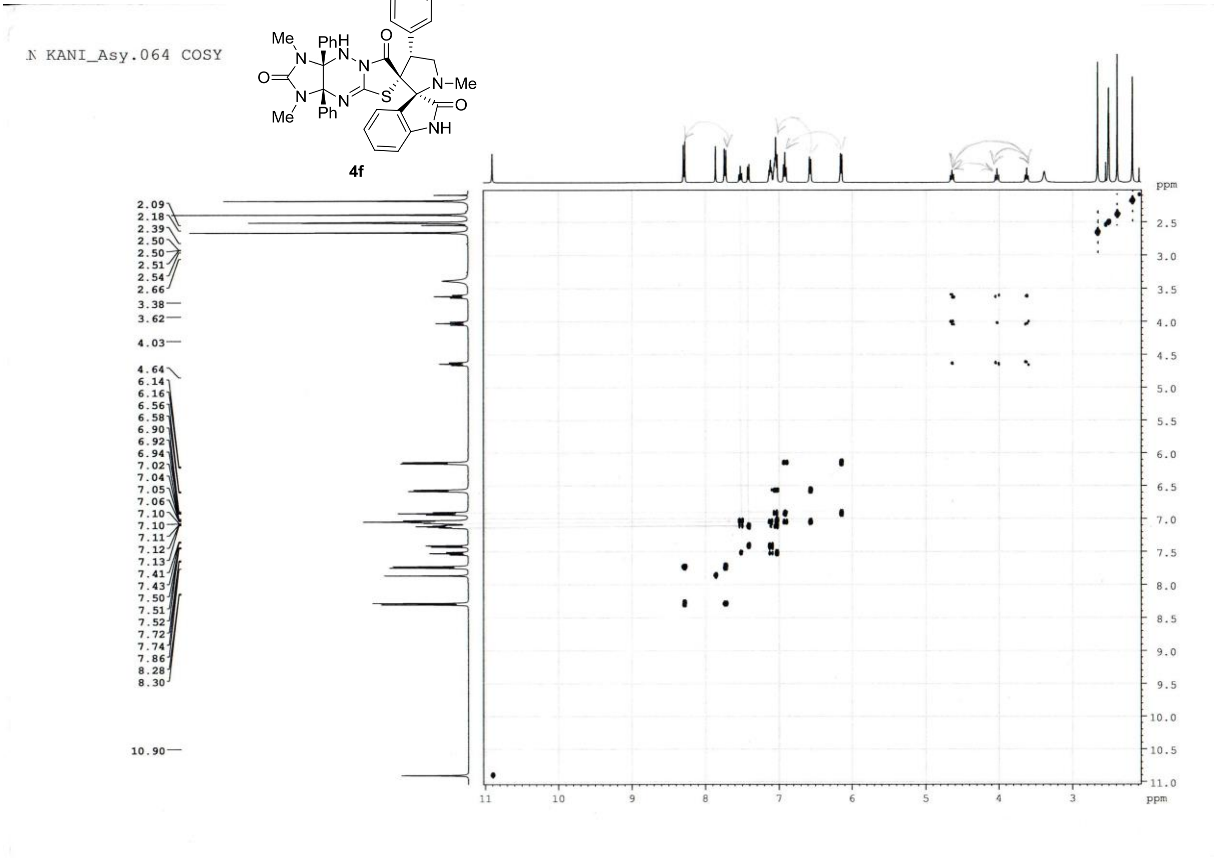

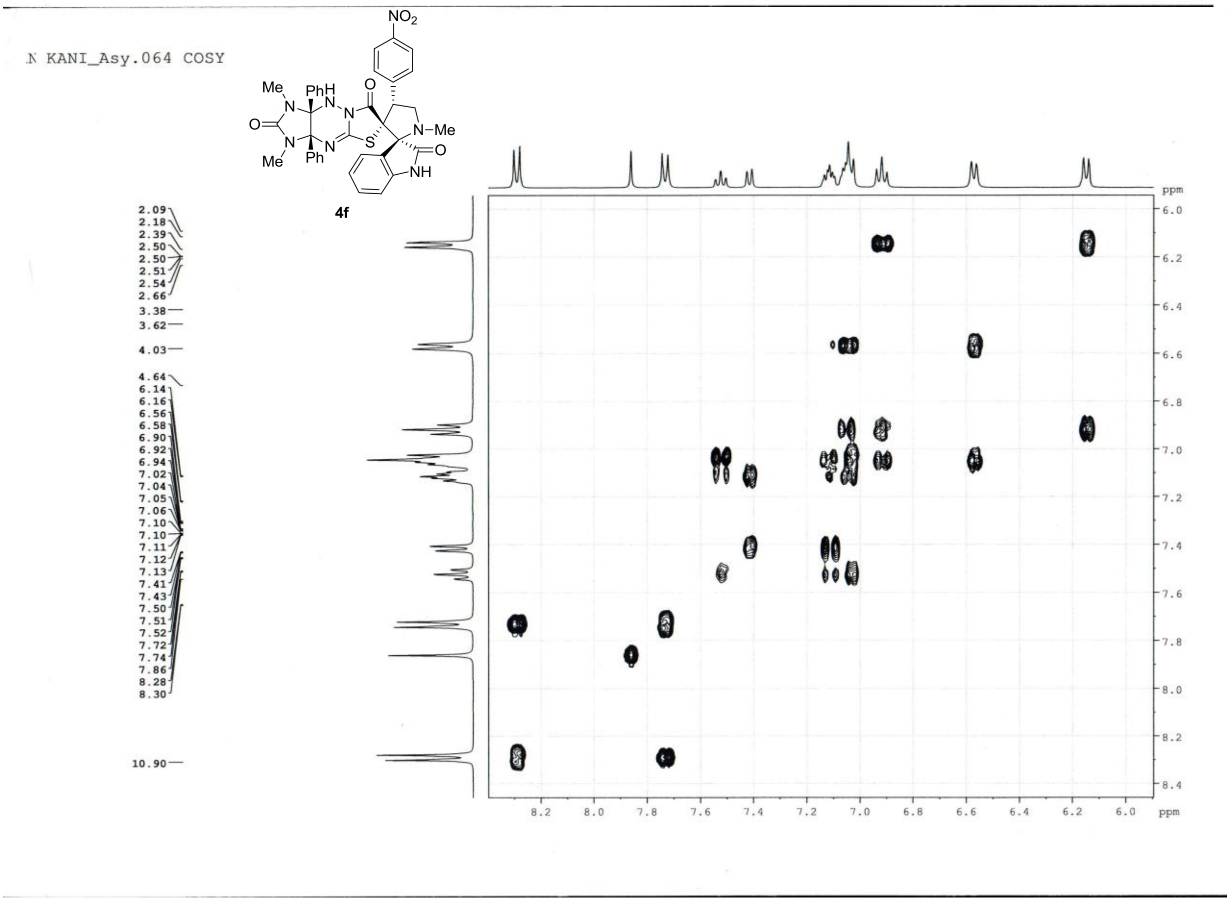

N KANI_Asy.064 COSY

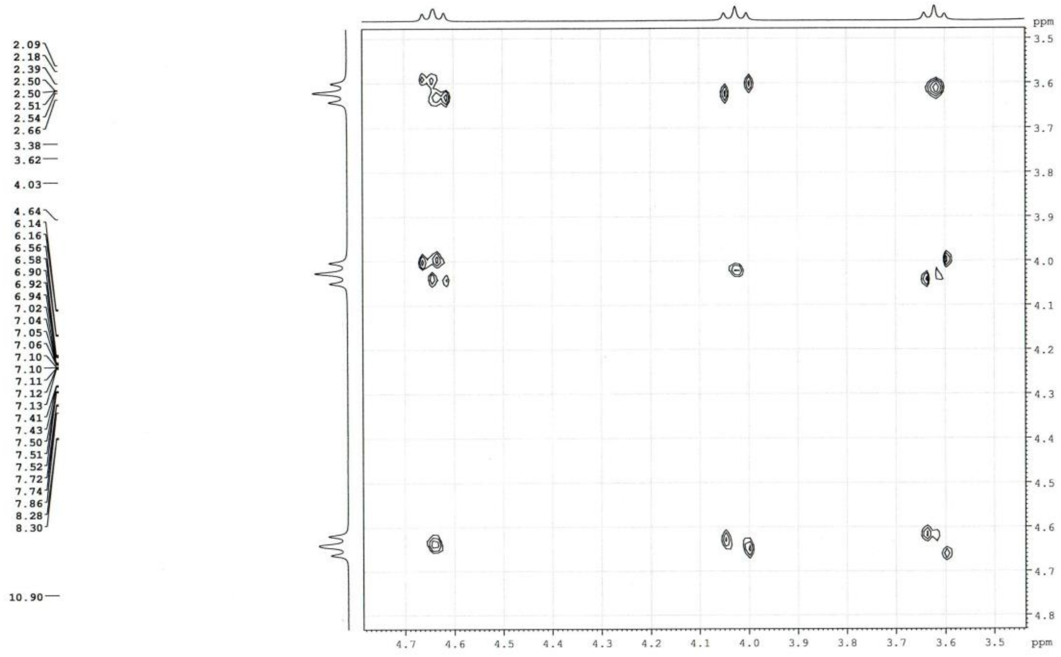

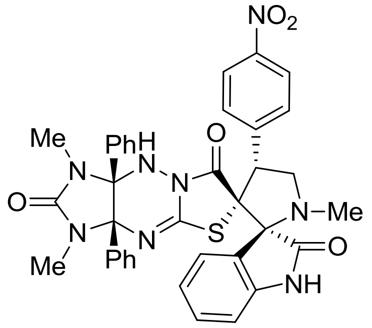

4f

{^{1}H-^{13}C} HSQC spectrum of **4f**

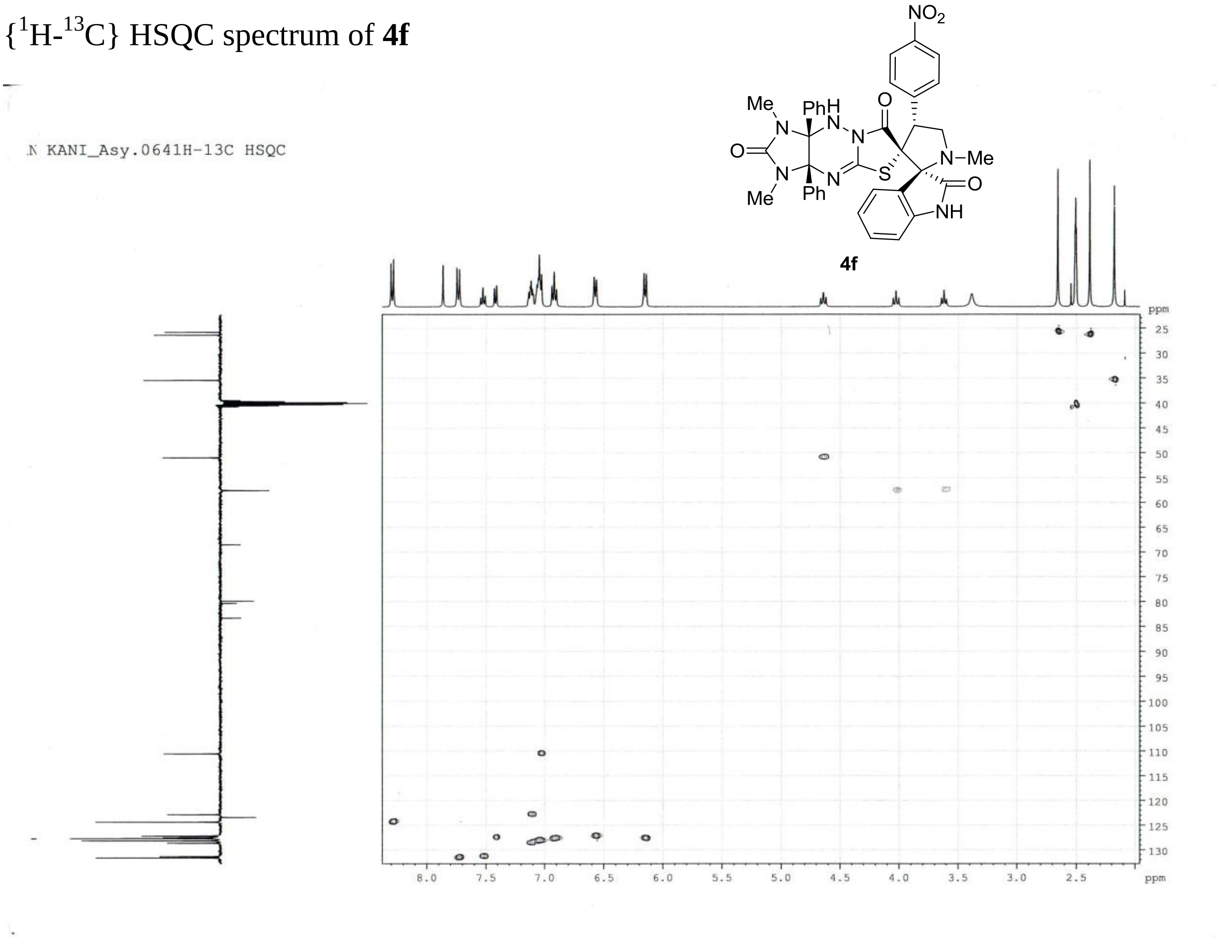

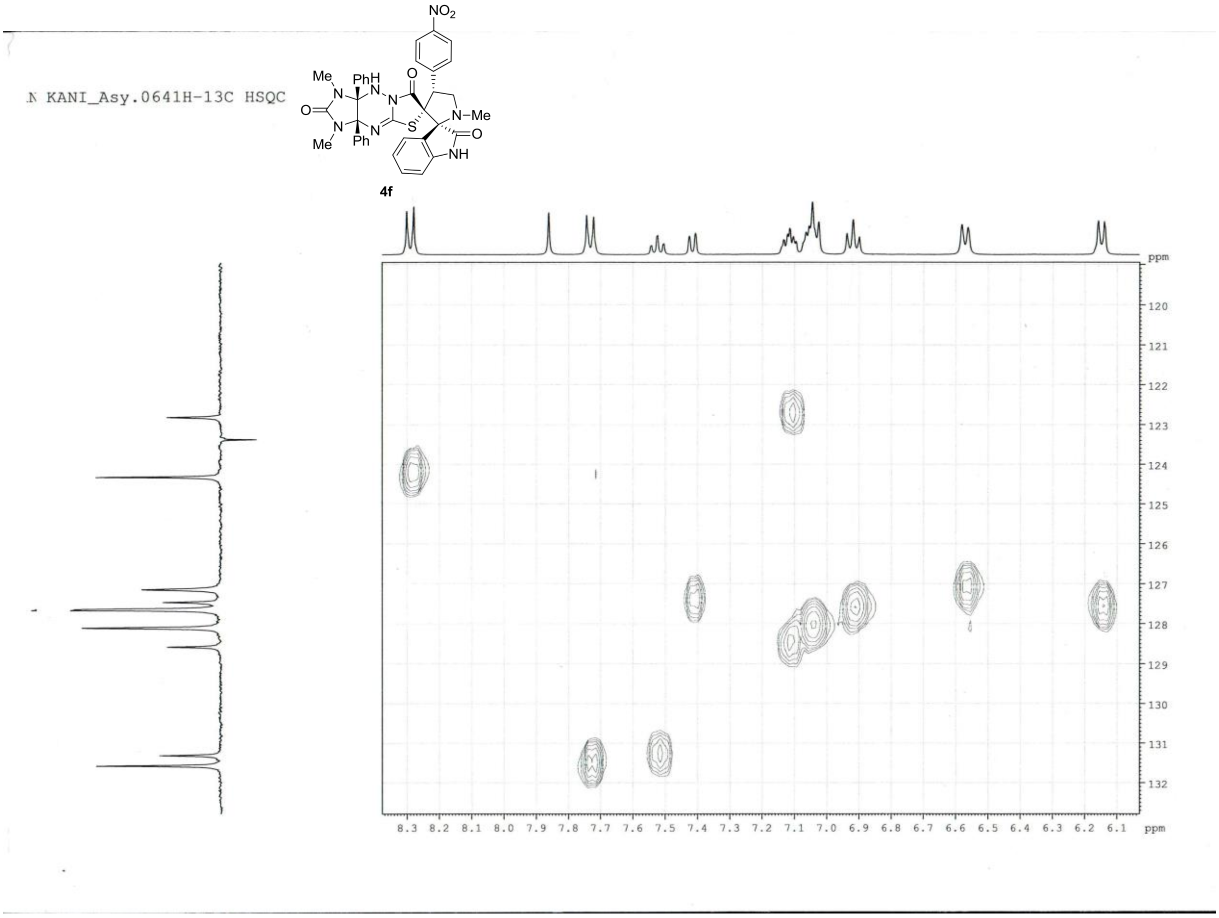

{^{1}H-^{13}C} and {^{1}H-^{15}N} HMBC spectra of **4f**

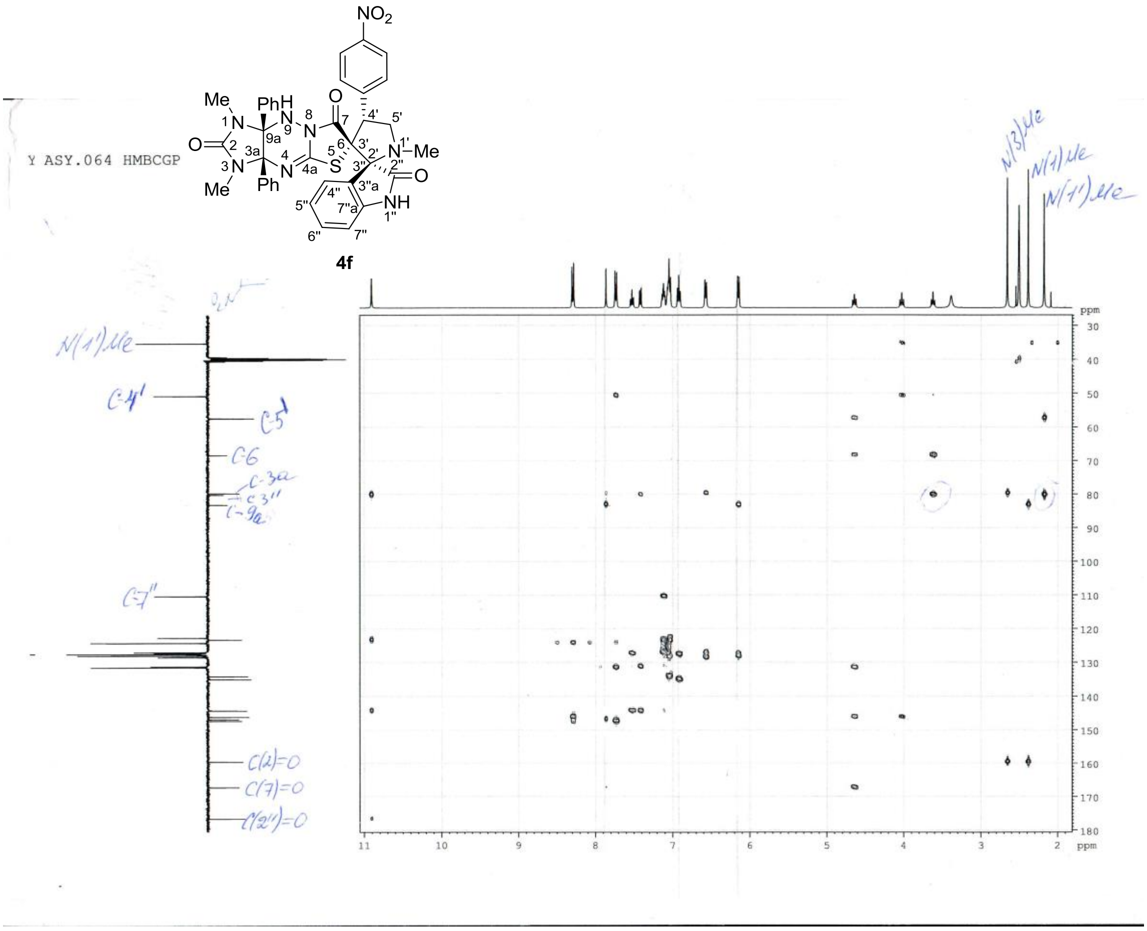

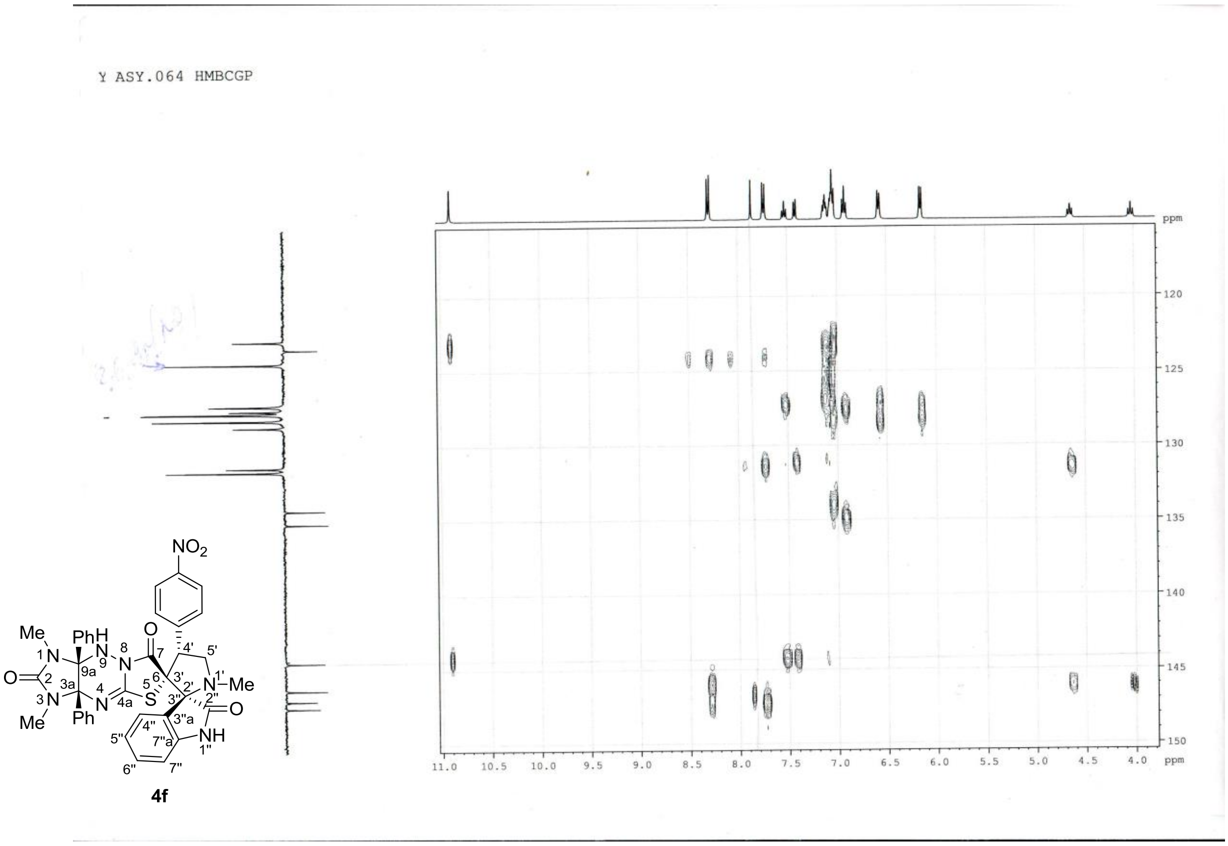

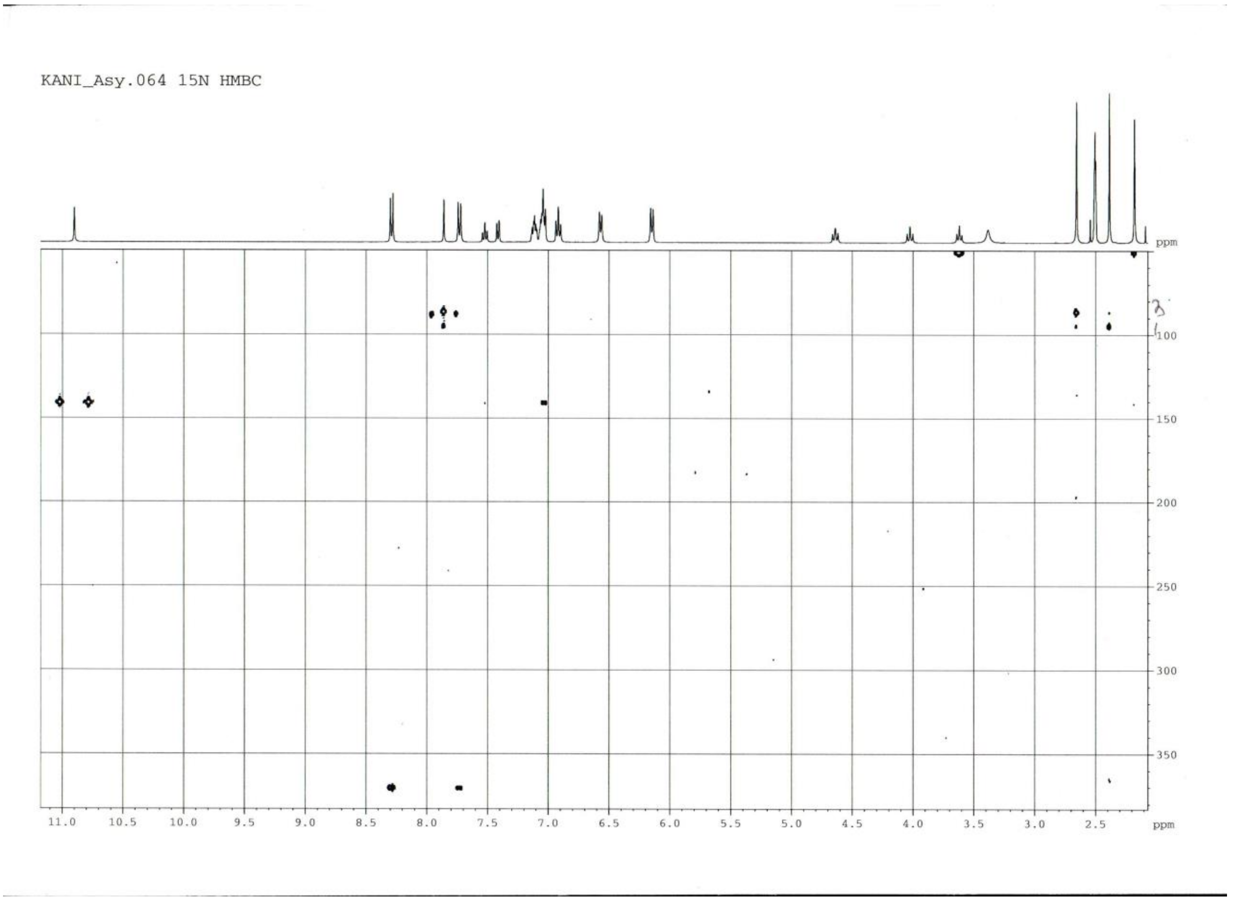
KANI_Asy.064 15N HMBC
ppm
100
150
200
250
300
350
11.0
10.5
10.0
9.5
9.0
8.5
8.0
7.5
7.0
6.5
6.0
5.5
5.0
4.5
4.0
3.5
3.0
2.5
ppm

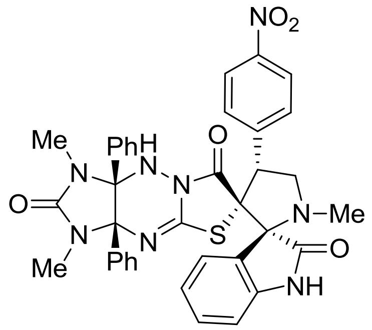
NO2
Me
Ph
H
N
O
N
N
N-Me
O
N
S
O
N
N
Me
Ph
NH

4f

^{1}H NMR spectrum of **4g**

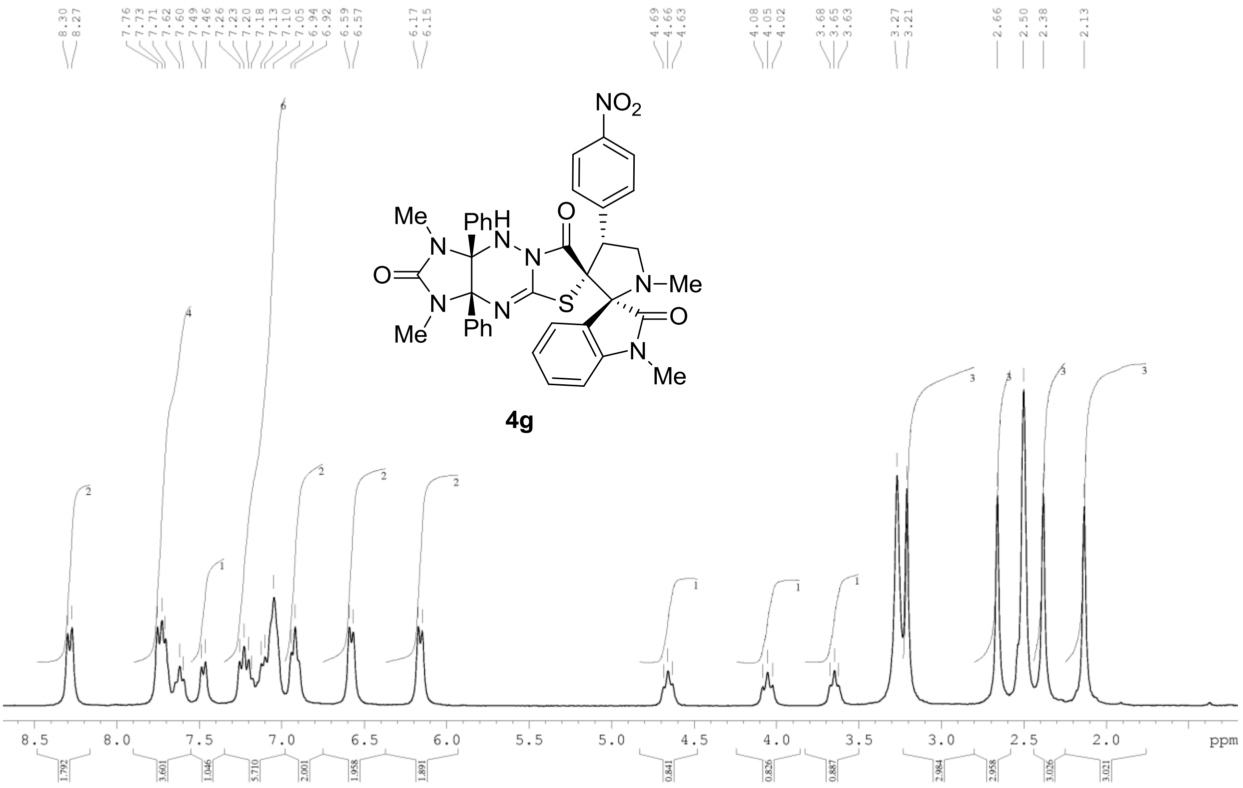

^{13}C NMR spectrum of **4g**

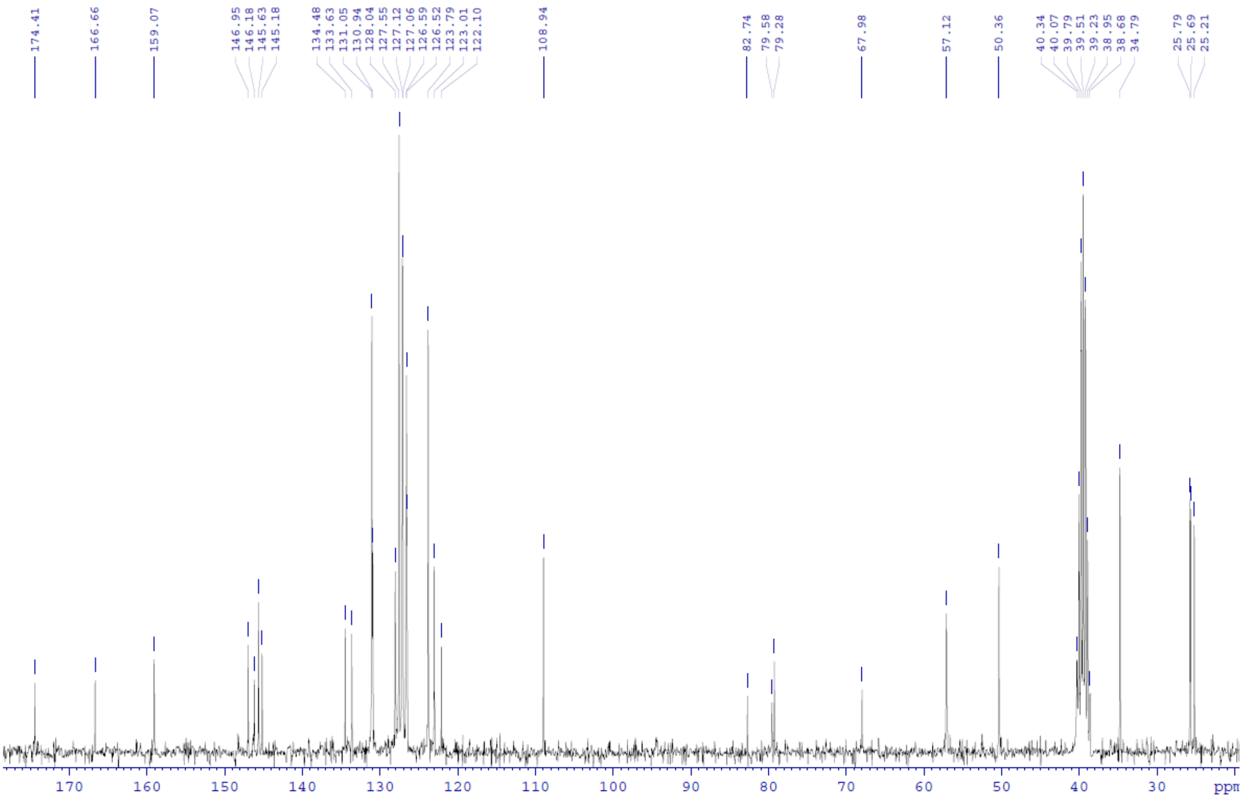

^{1}H NMR spectrum of **4h**

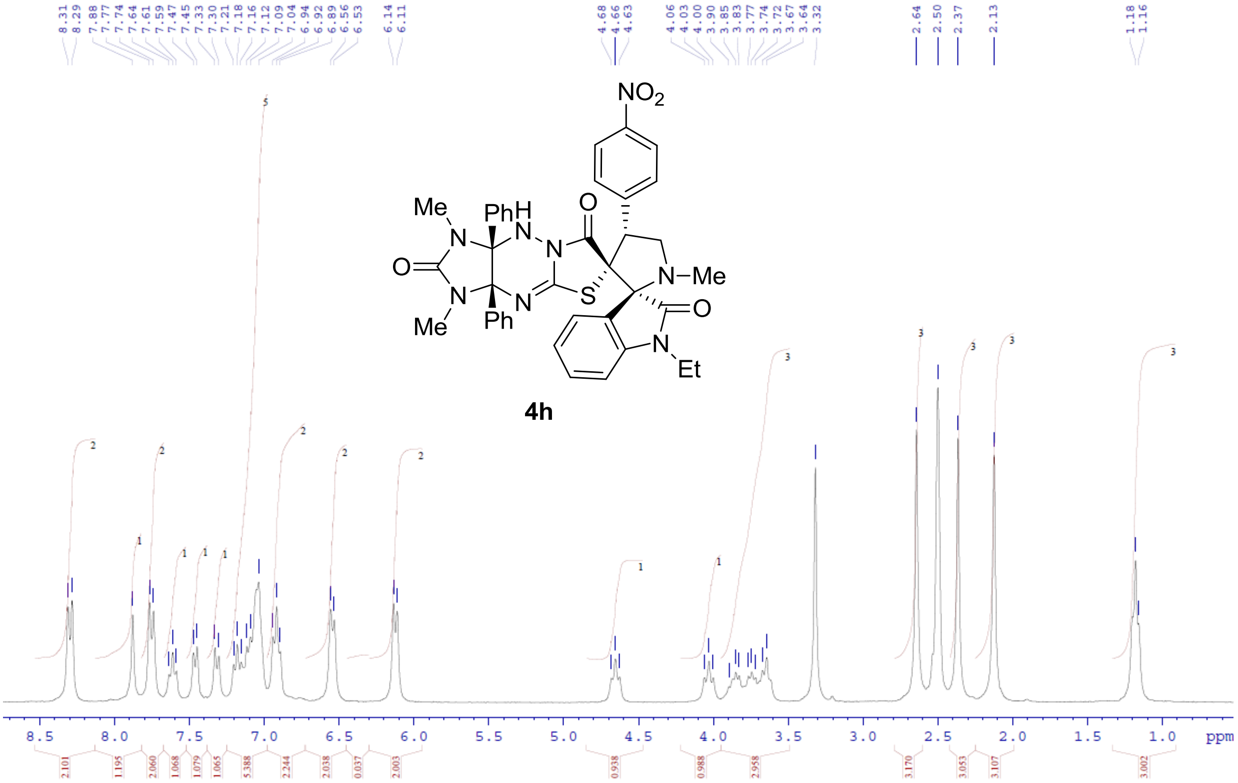

^{13}C NMR spectrum of **4h**

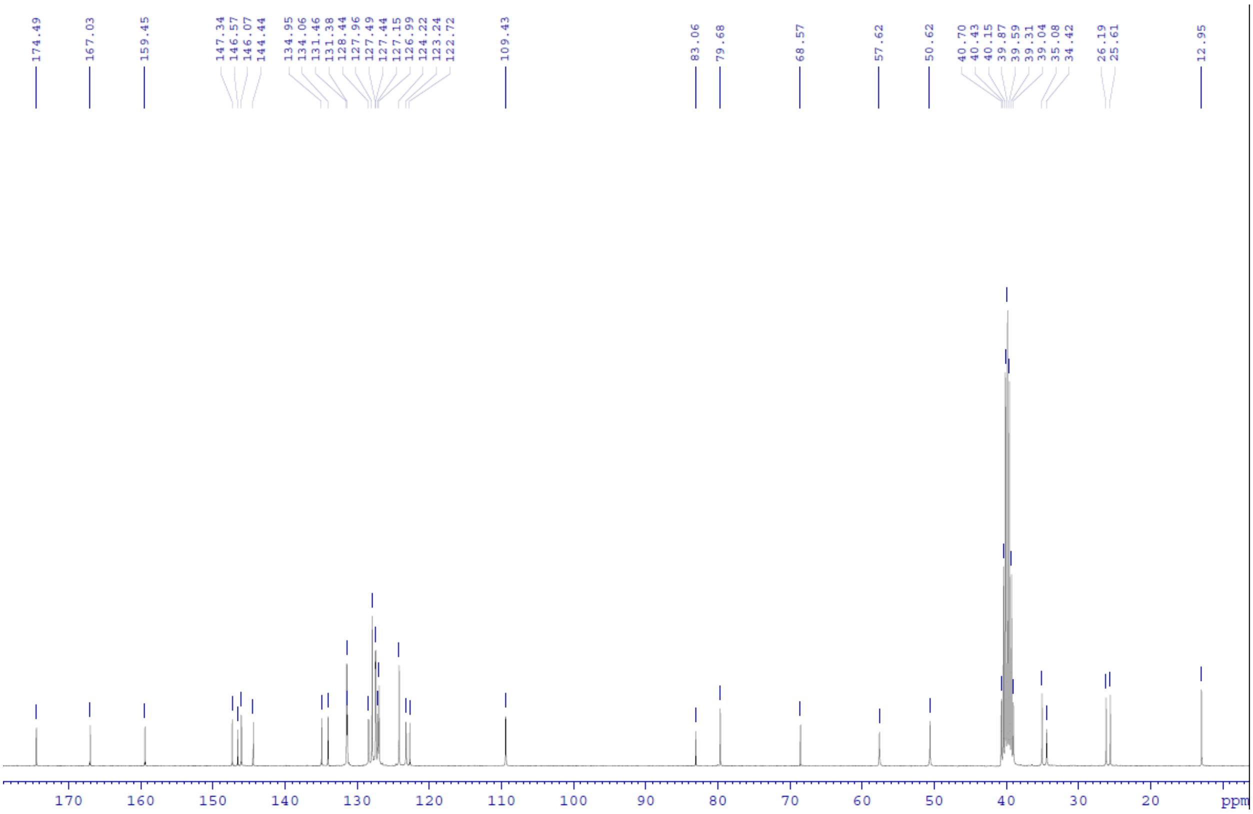

^{1}H NMR spectrum of **4i**

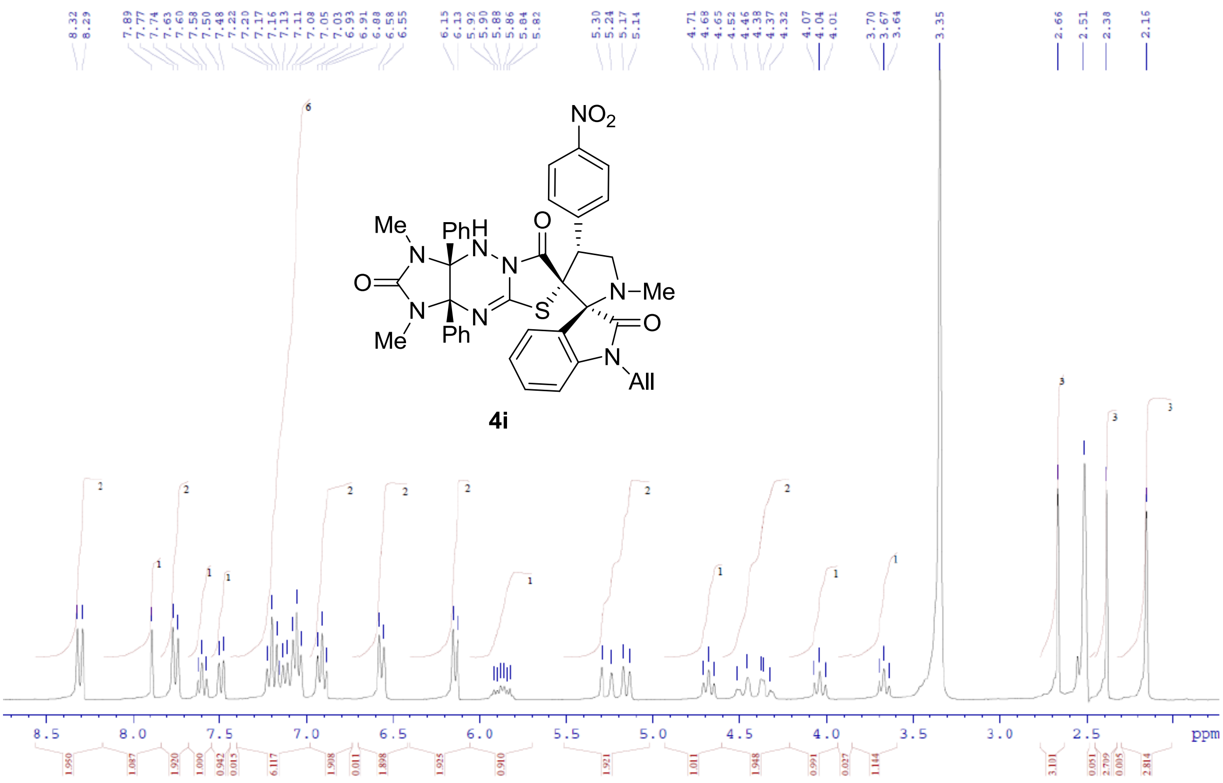

^{13}C NMR spectrum of **4i**

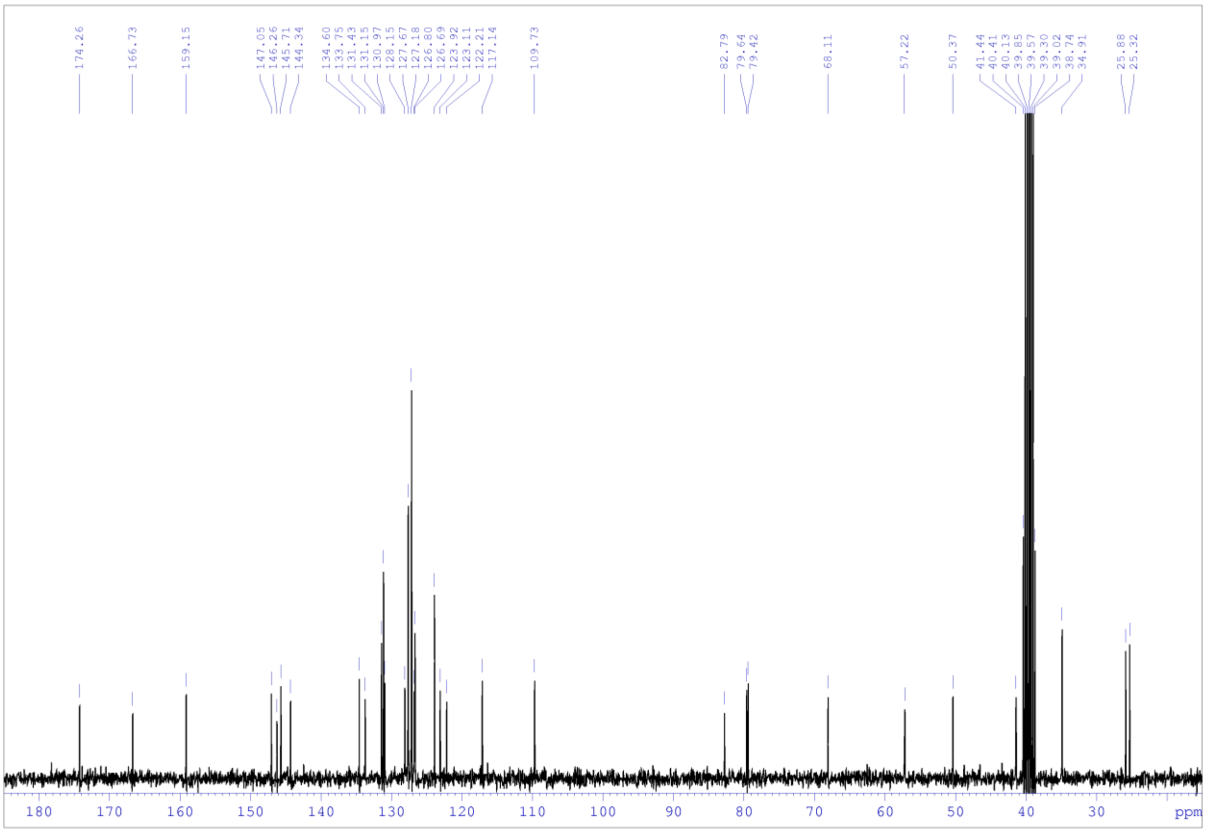

^{1}H NMR spectrum of **4j**

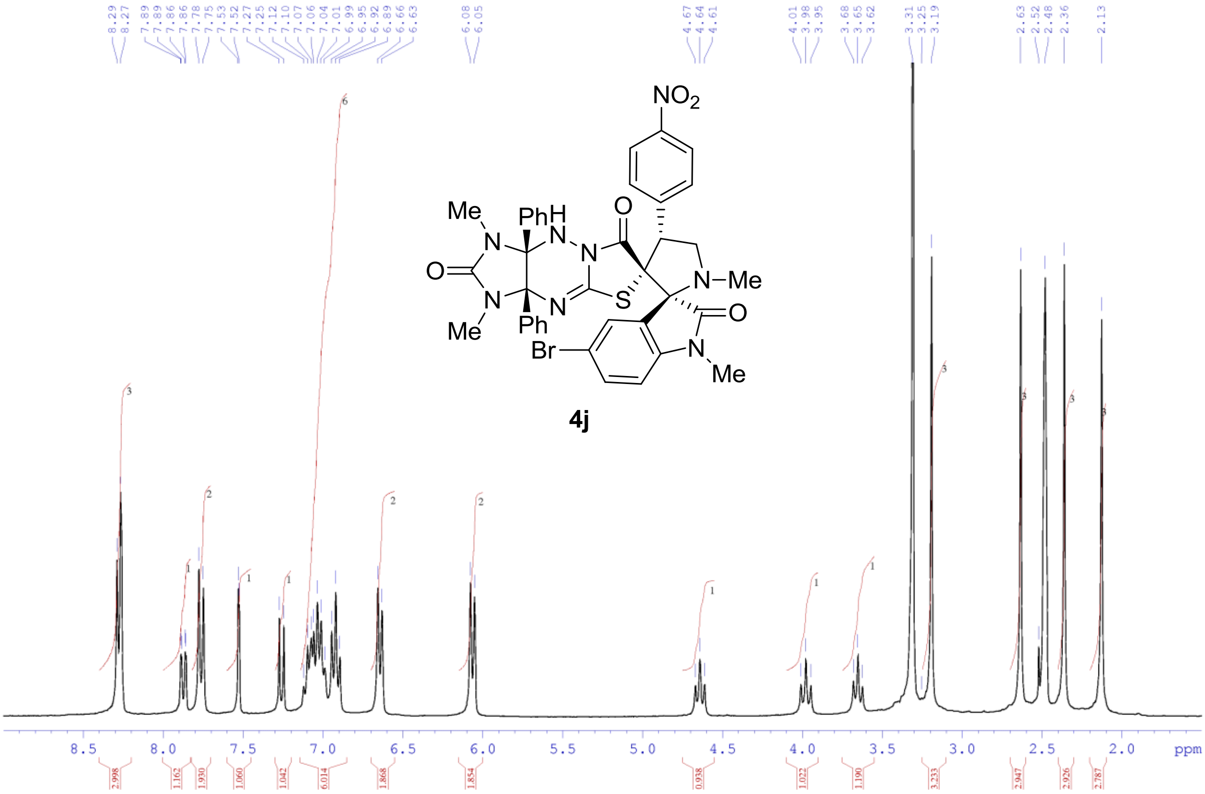

^{13}C NMR spectrum of **4j**

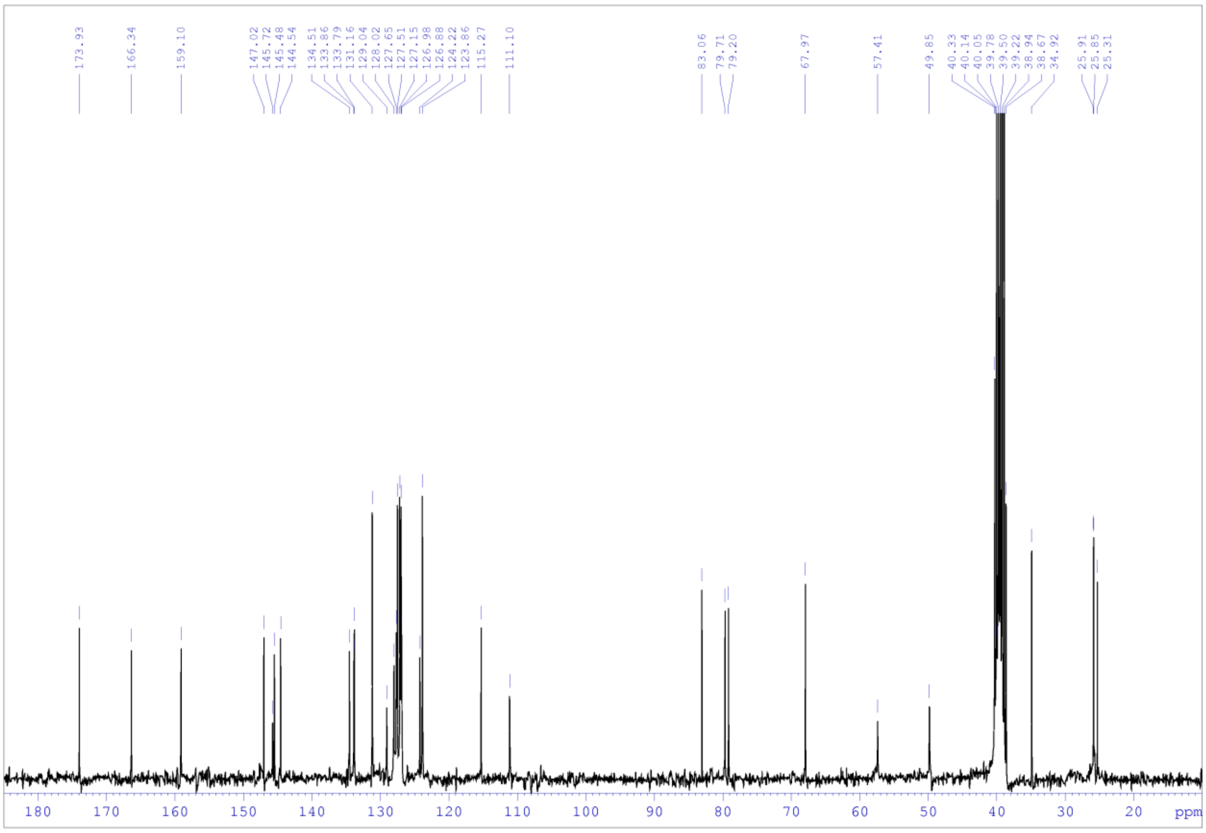

^{1}H NMR spectrum of **4k**

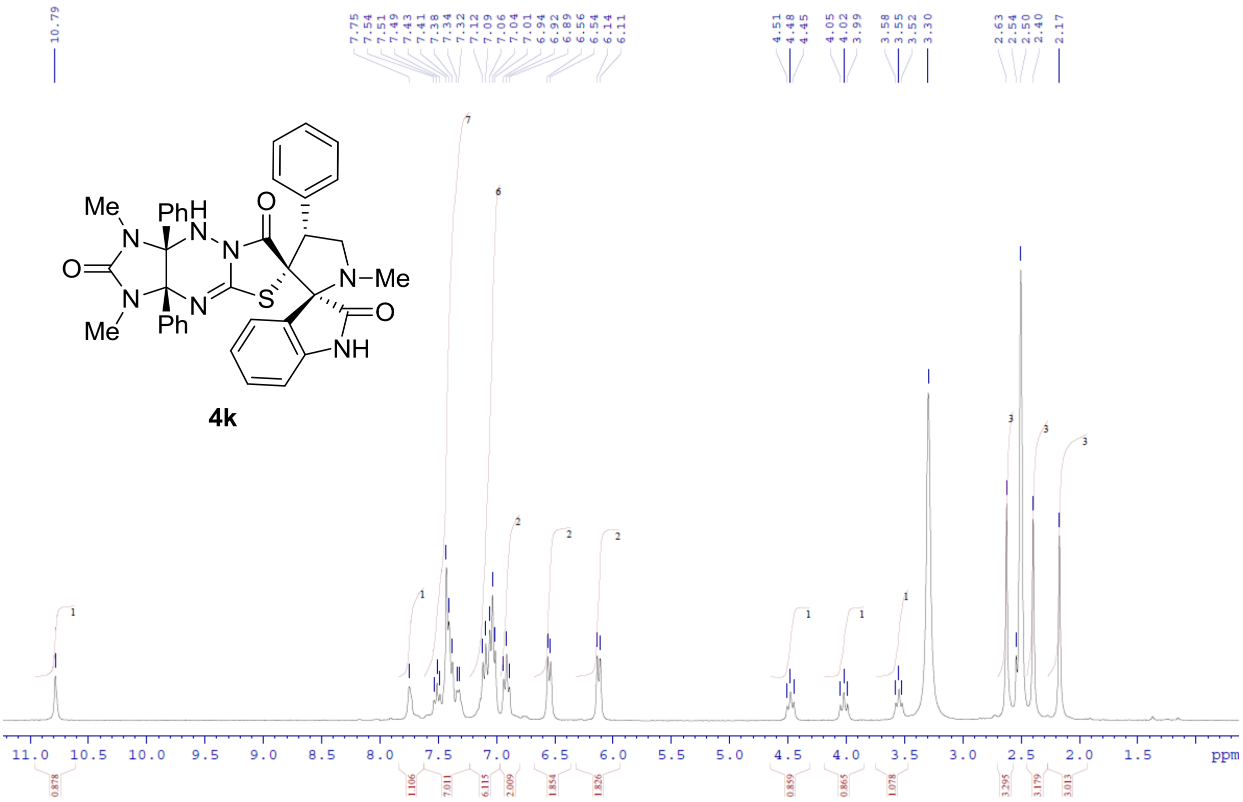

^{13}C NMR spectrum of **4k**

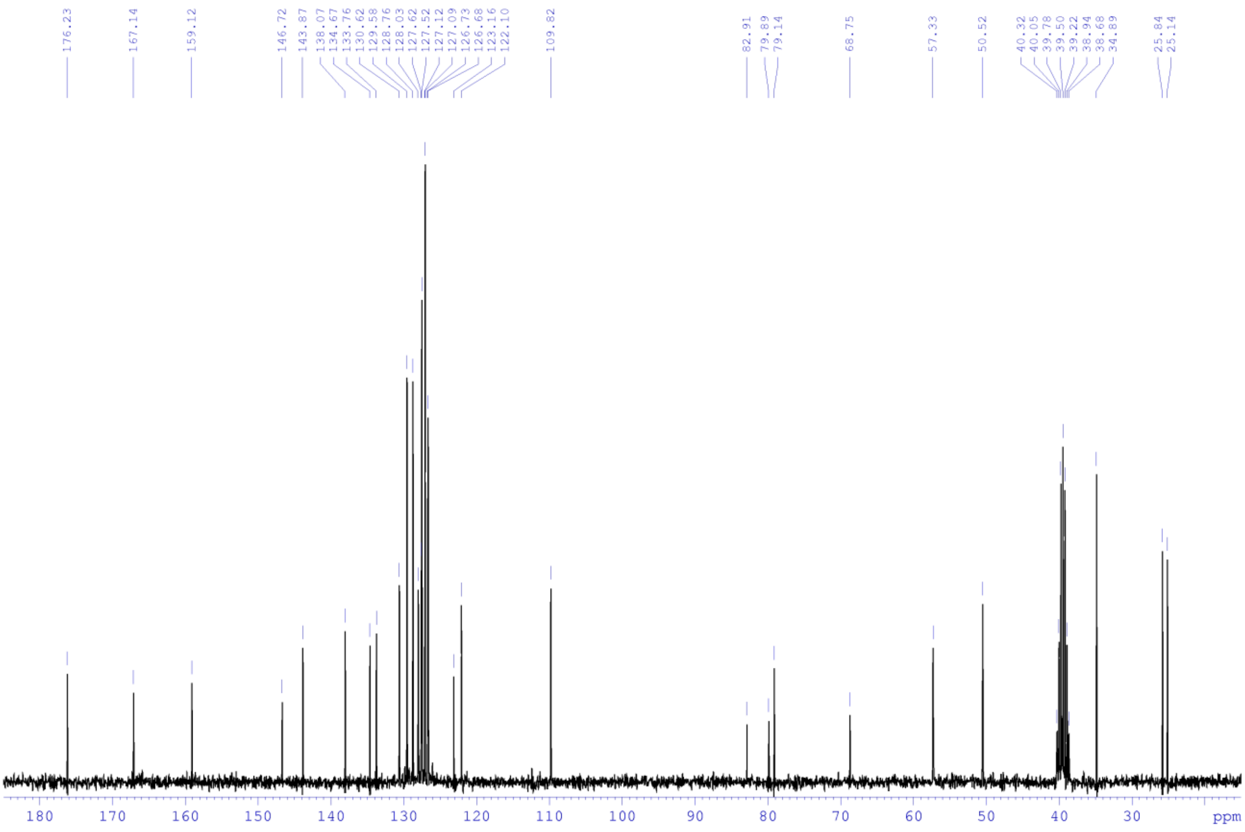

^{1}H NMR spectrum of **4l**

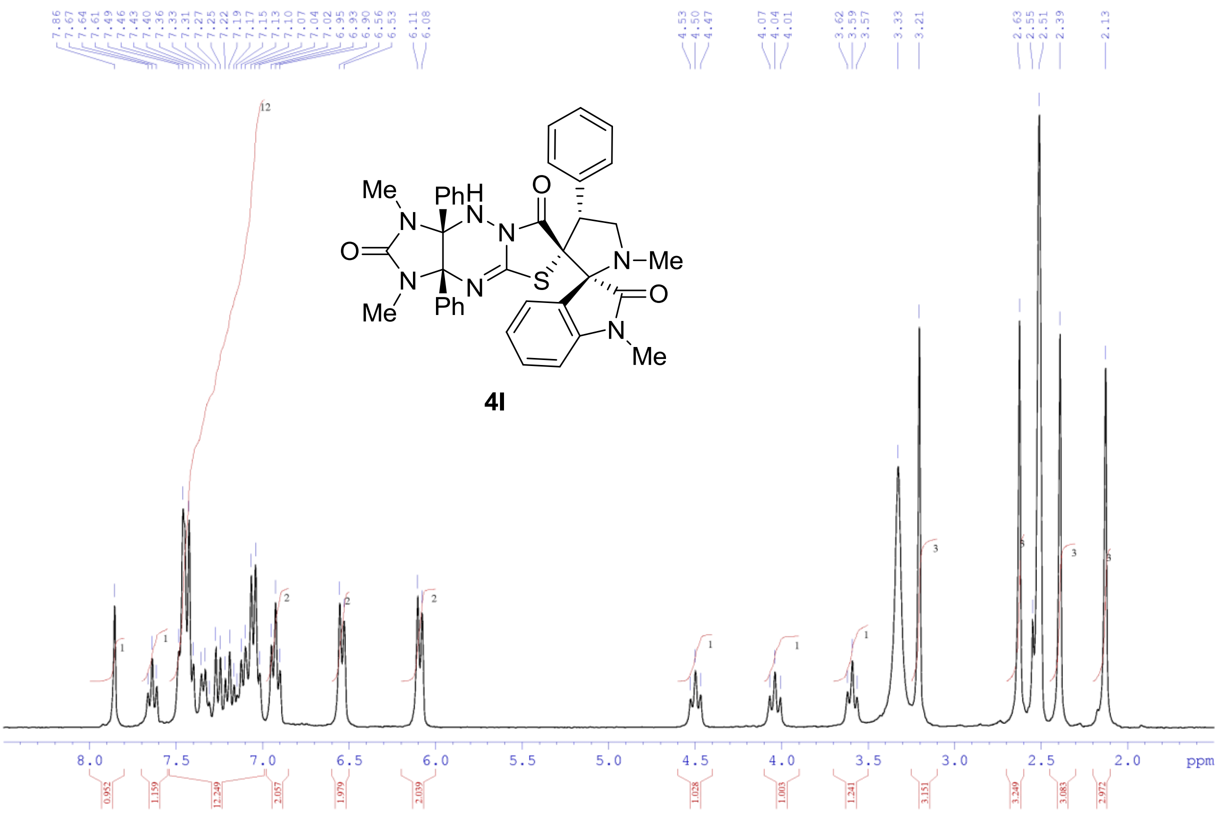

^{13}C NMR spectrum of **4l**

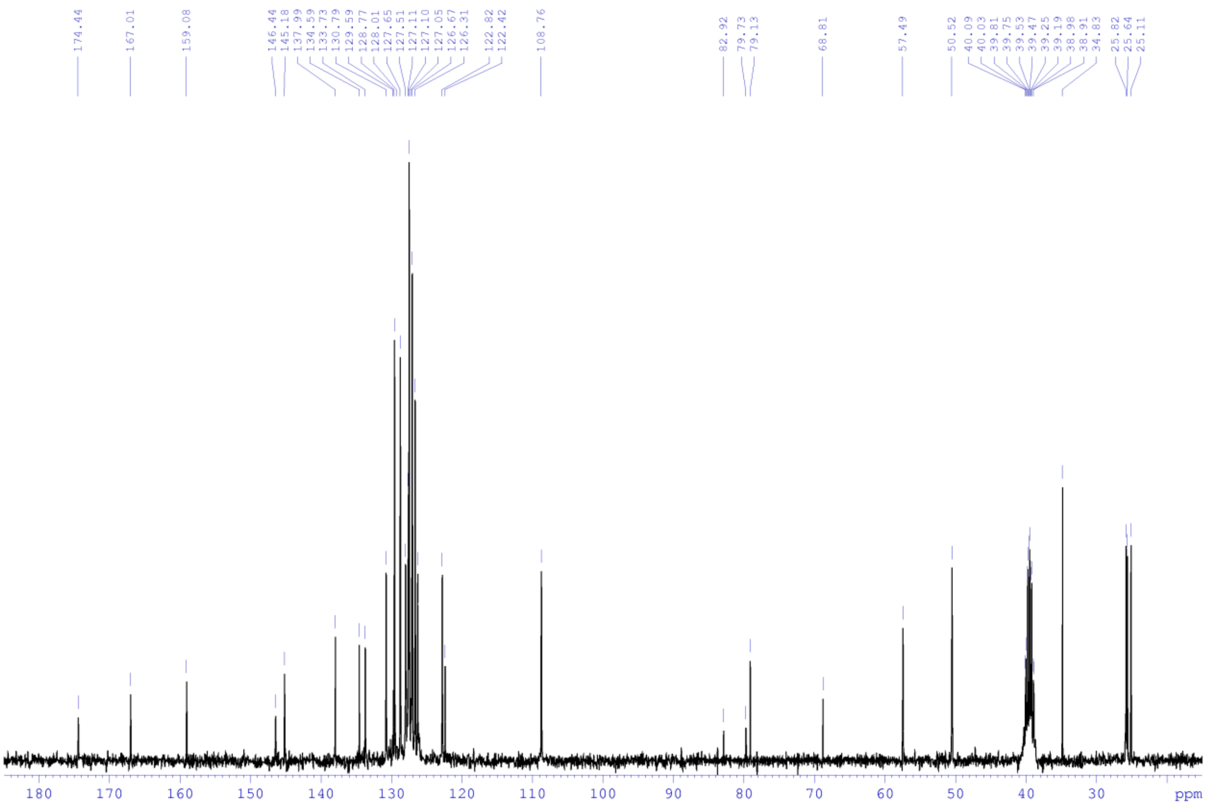

^{1}H NMR spectrum of **4m**

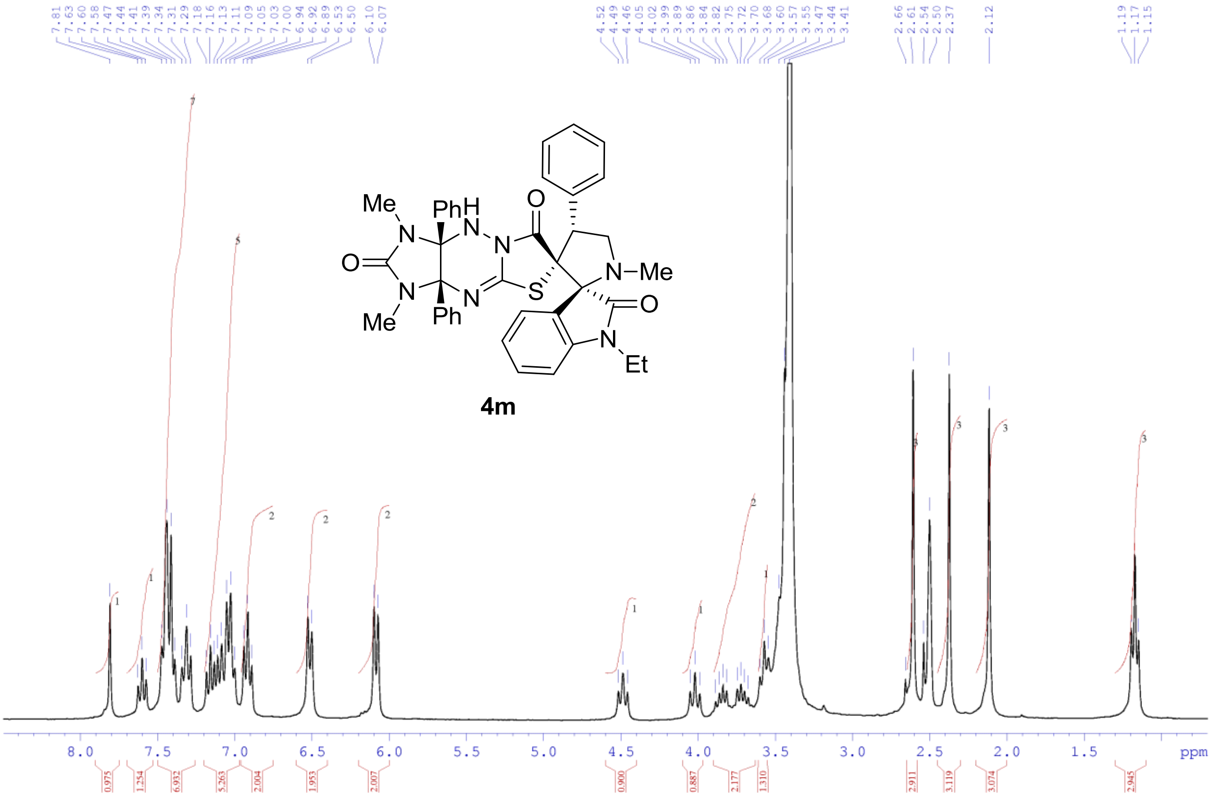

^{13}C NMR spectrum of **4m**

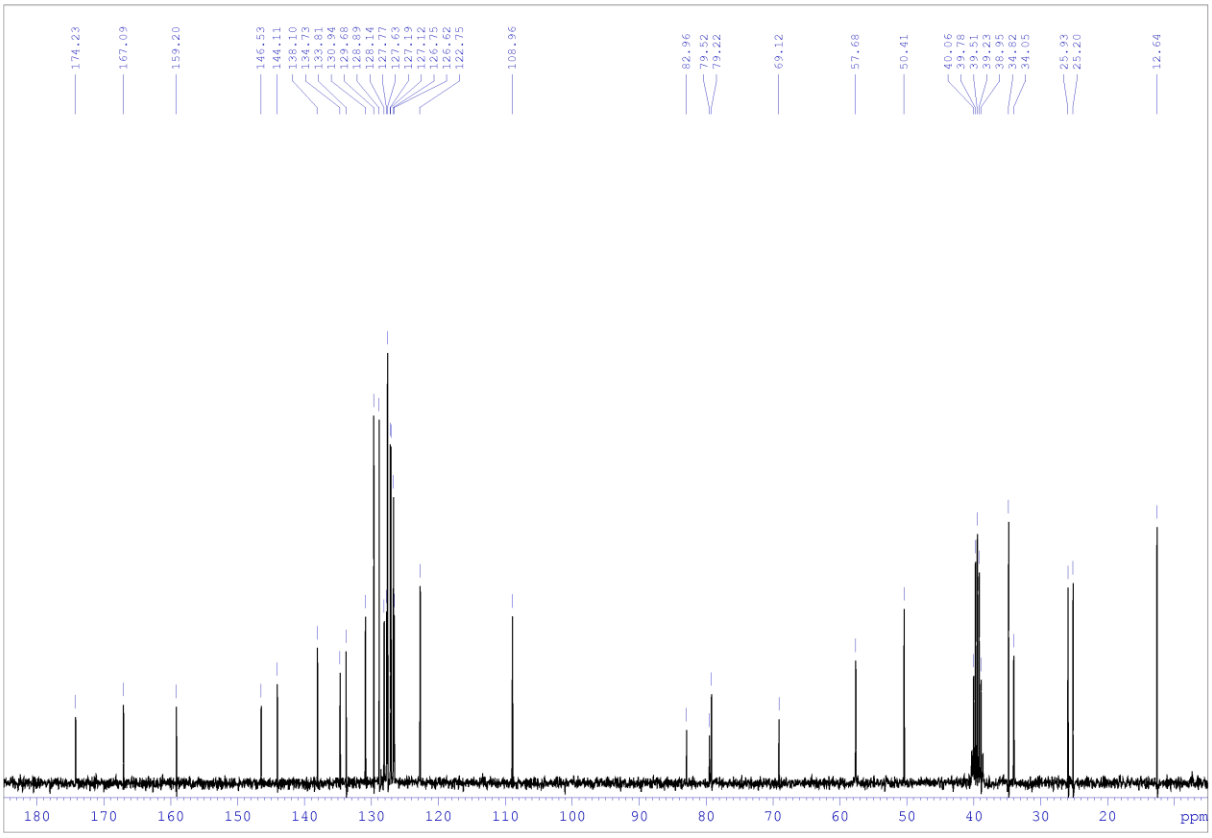

^{1}H NMR spectrum of **4n**

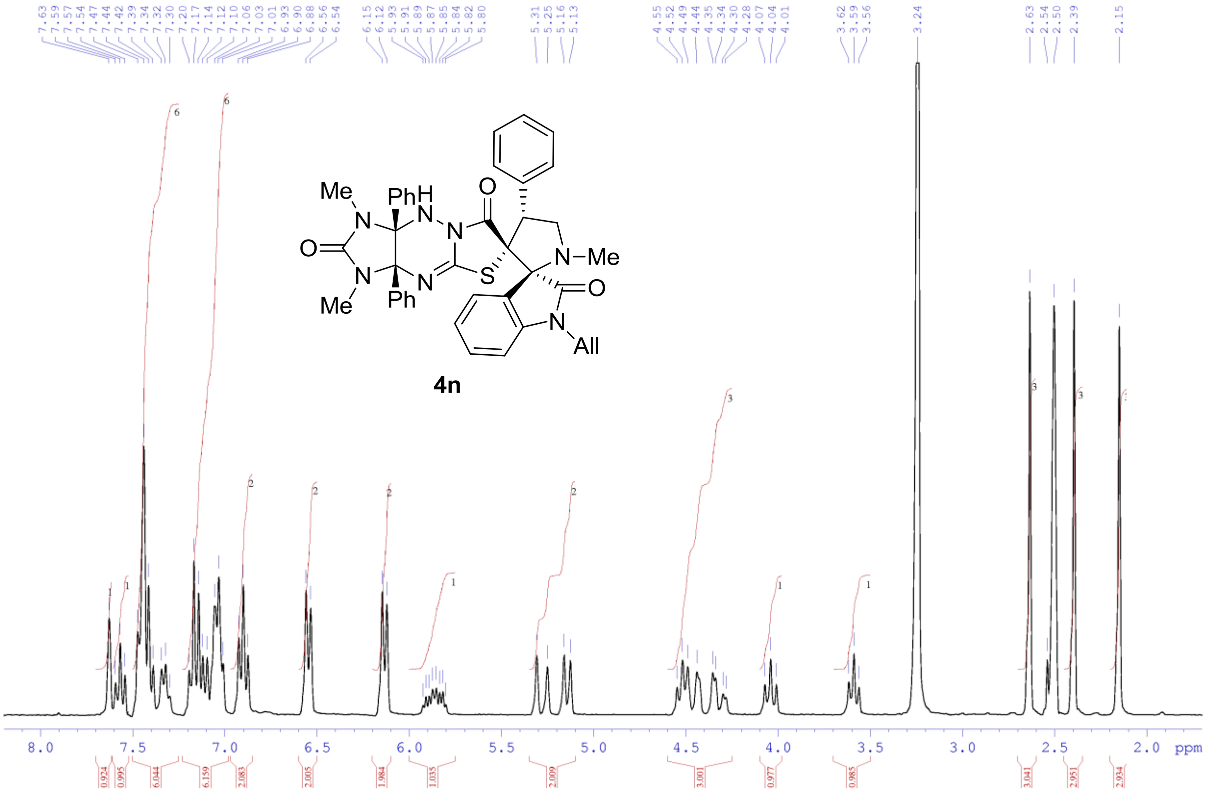

^{13}C NMR spectrum of **4n**

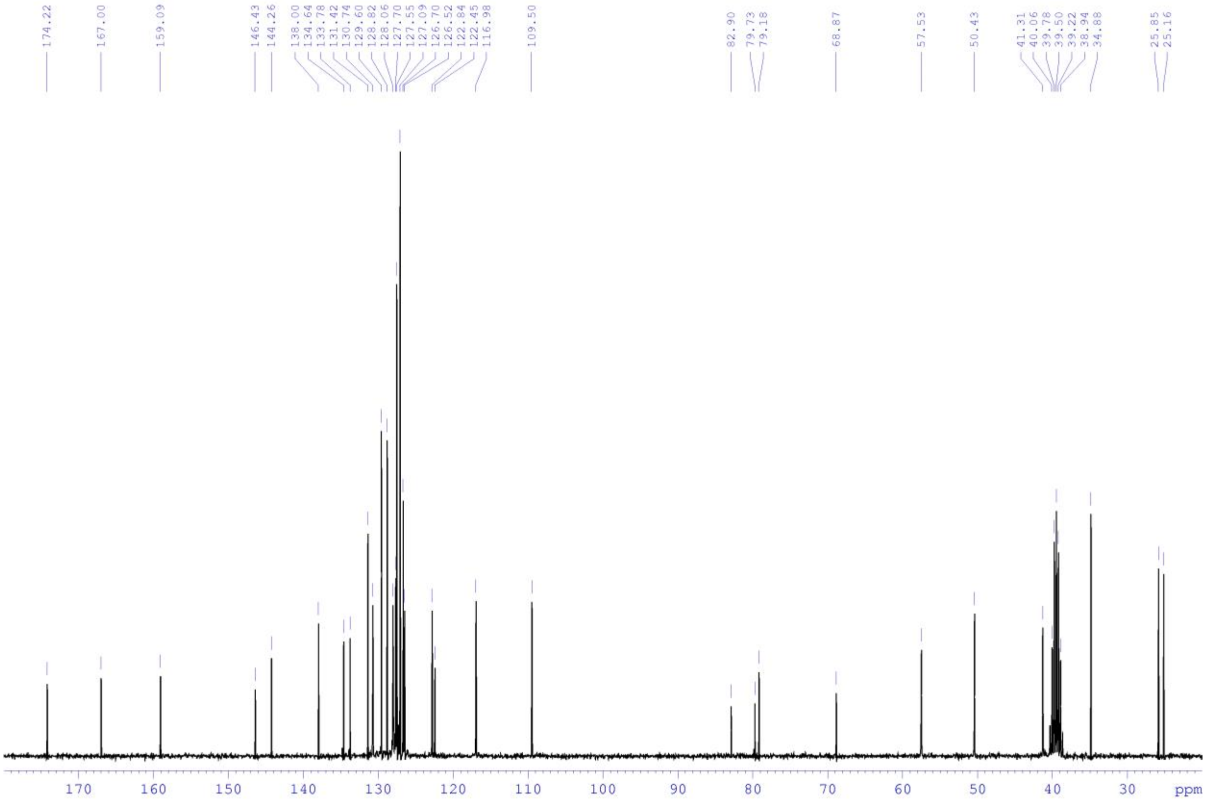

^{1}H NMR spectrum of **4o**

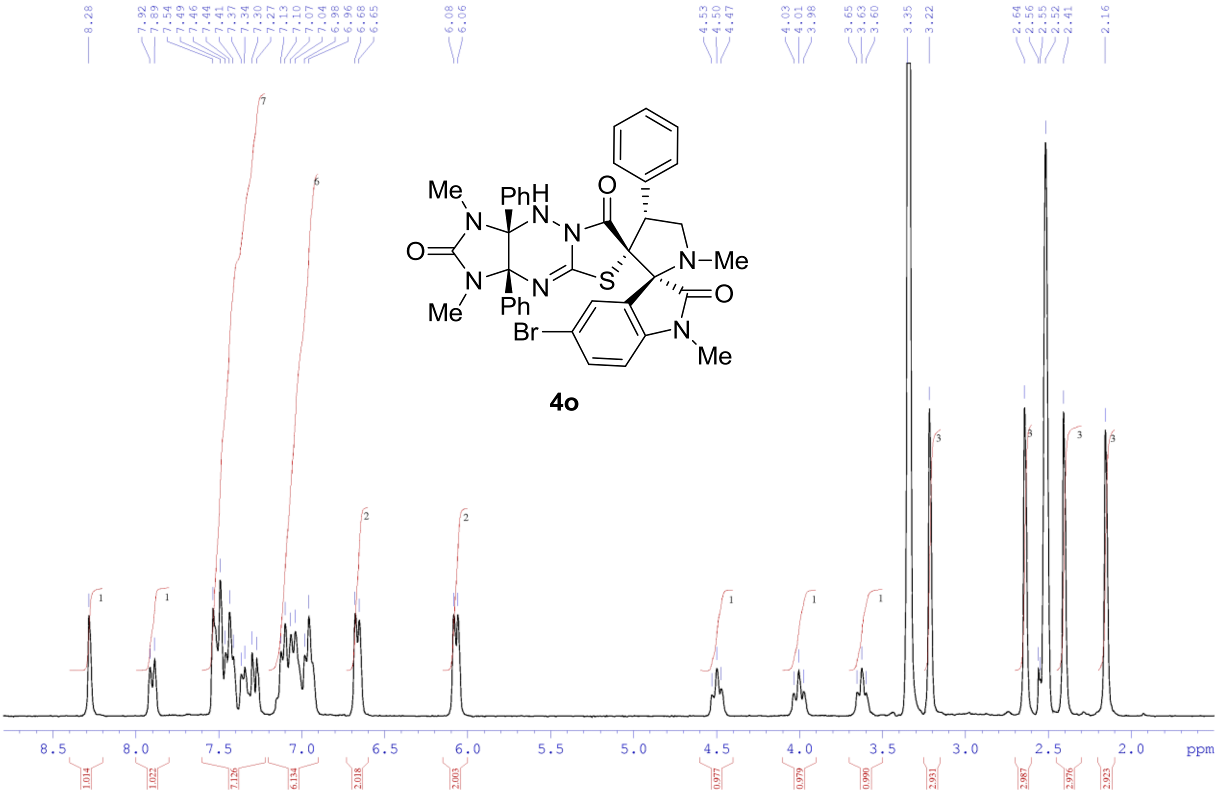

^{13}C NMR spectrum of **4o**

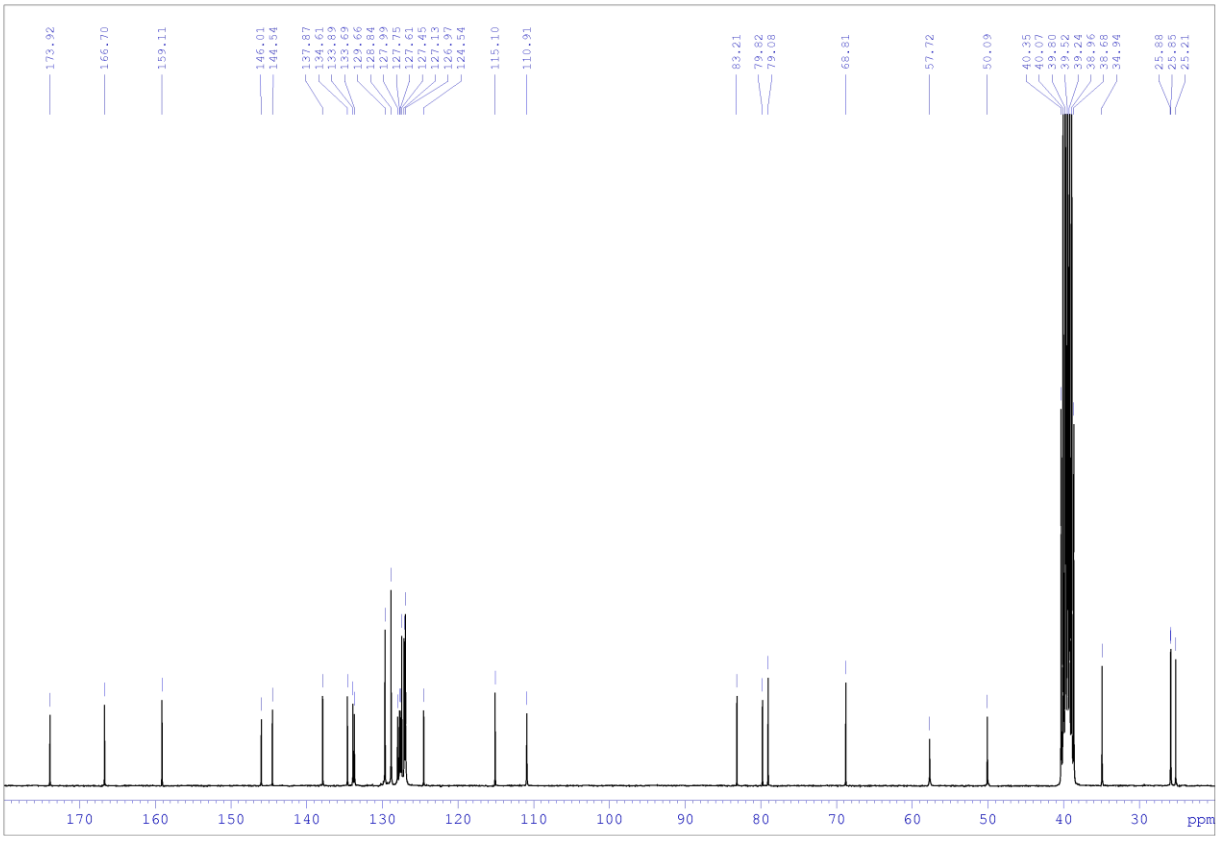

^{1}H NMR spectrum of **4p**

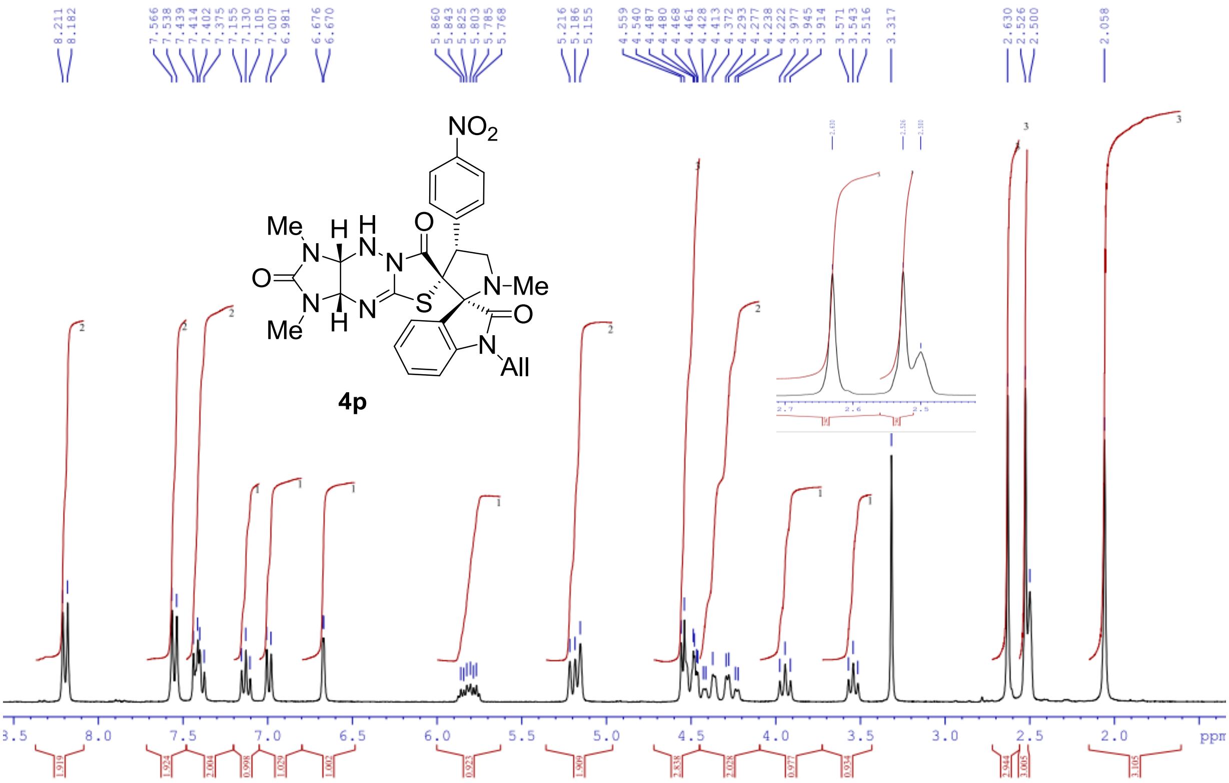

^{13}C NMR spectrum of **4p**

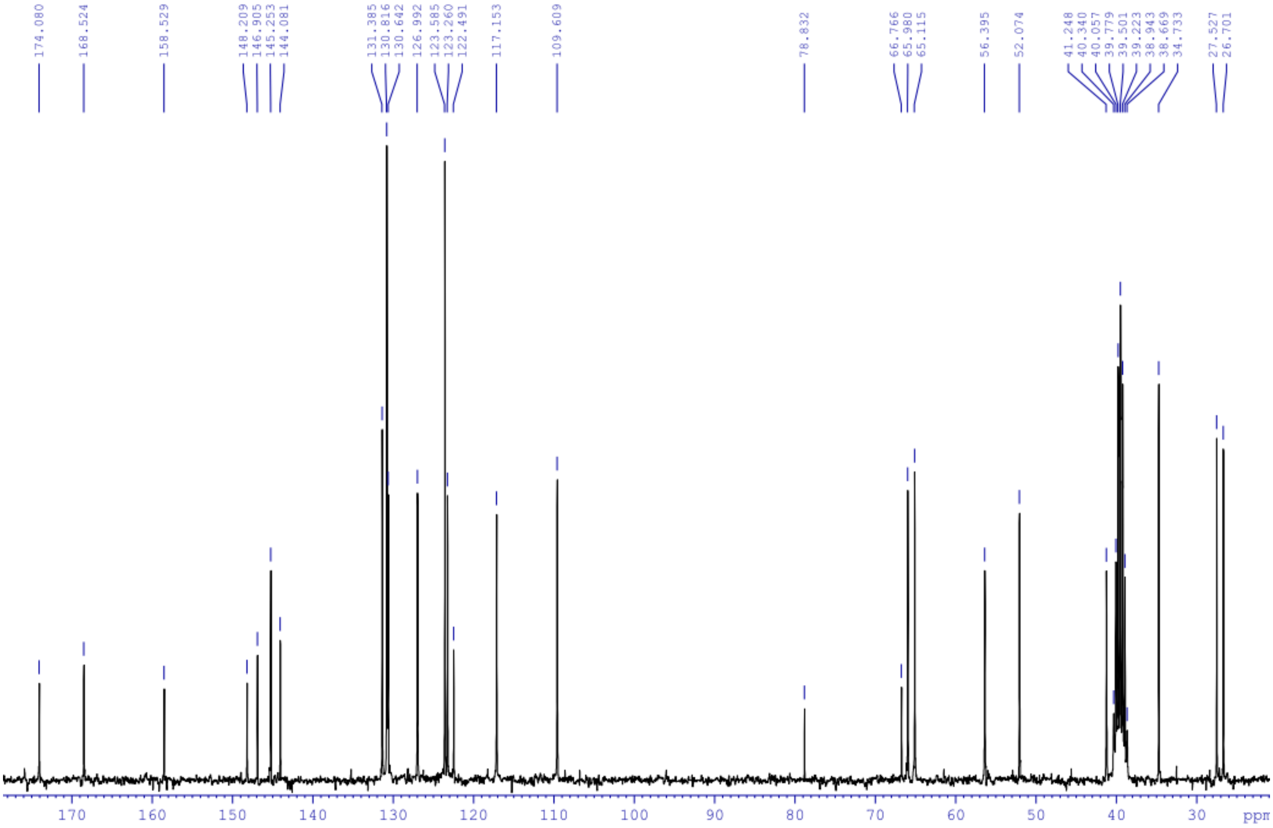

^{1}H NMR spectrum of **4q**

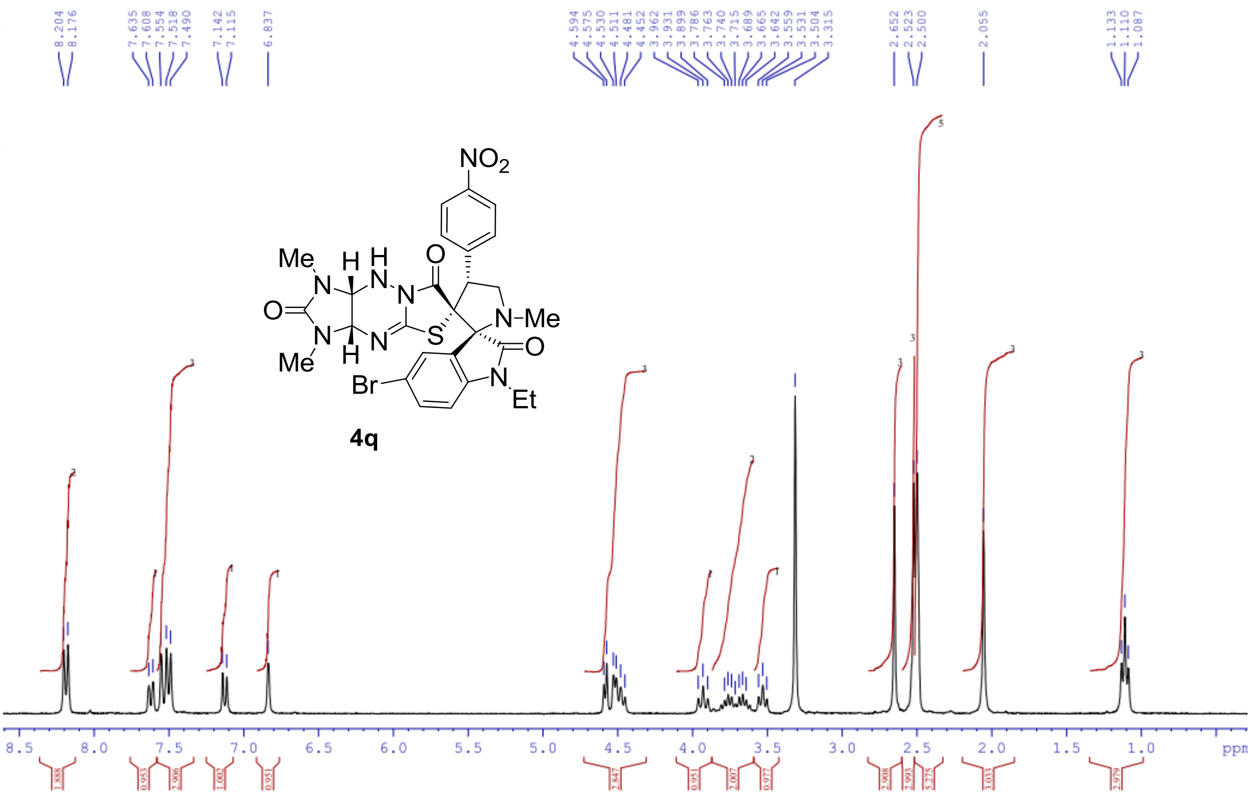

^{13}C NMR spectrum of **4q**

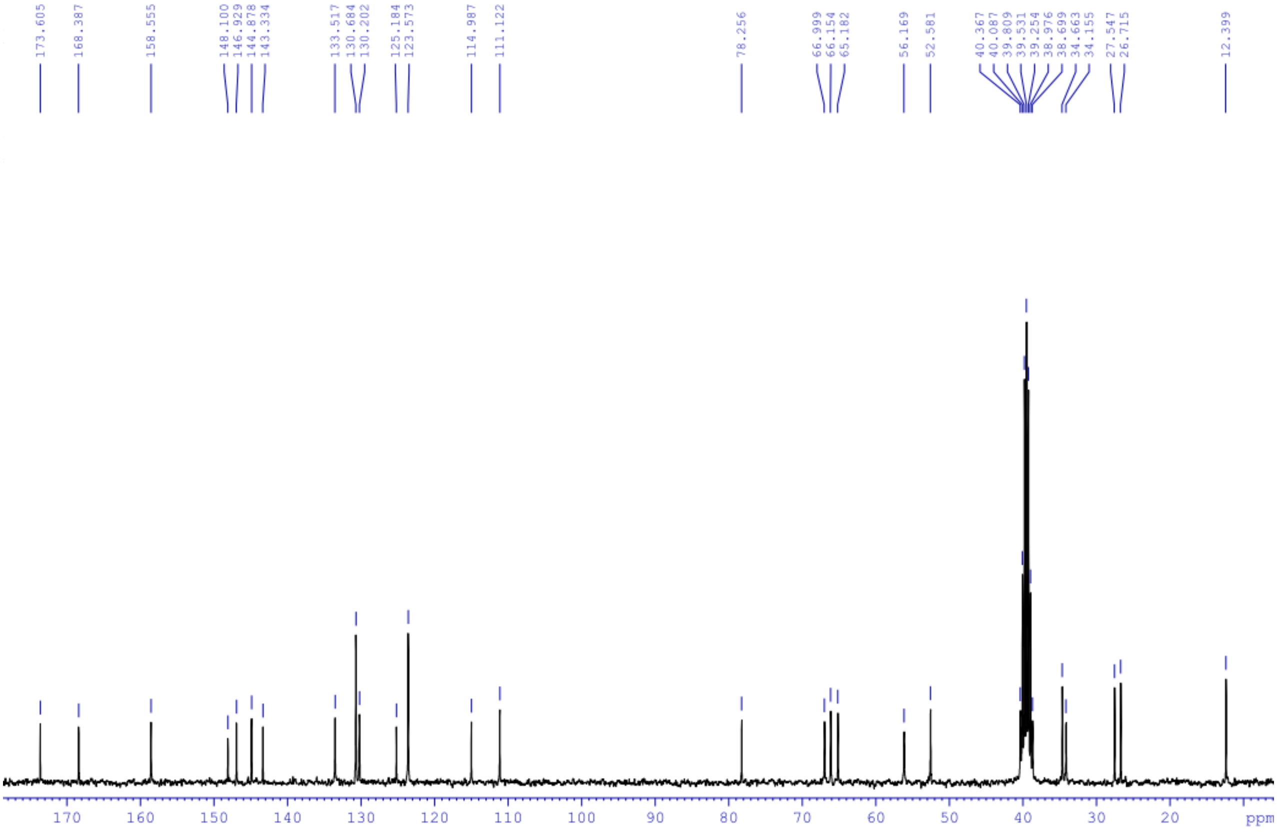

^{1}H NMR spectrum of **4r**

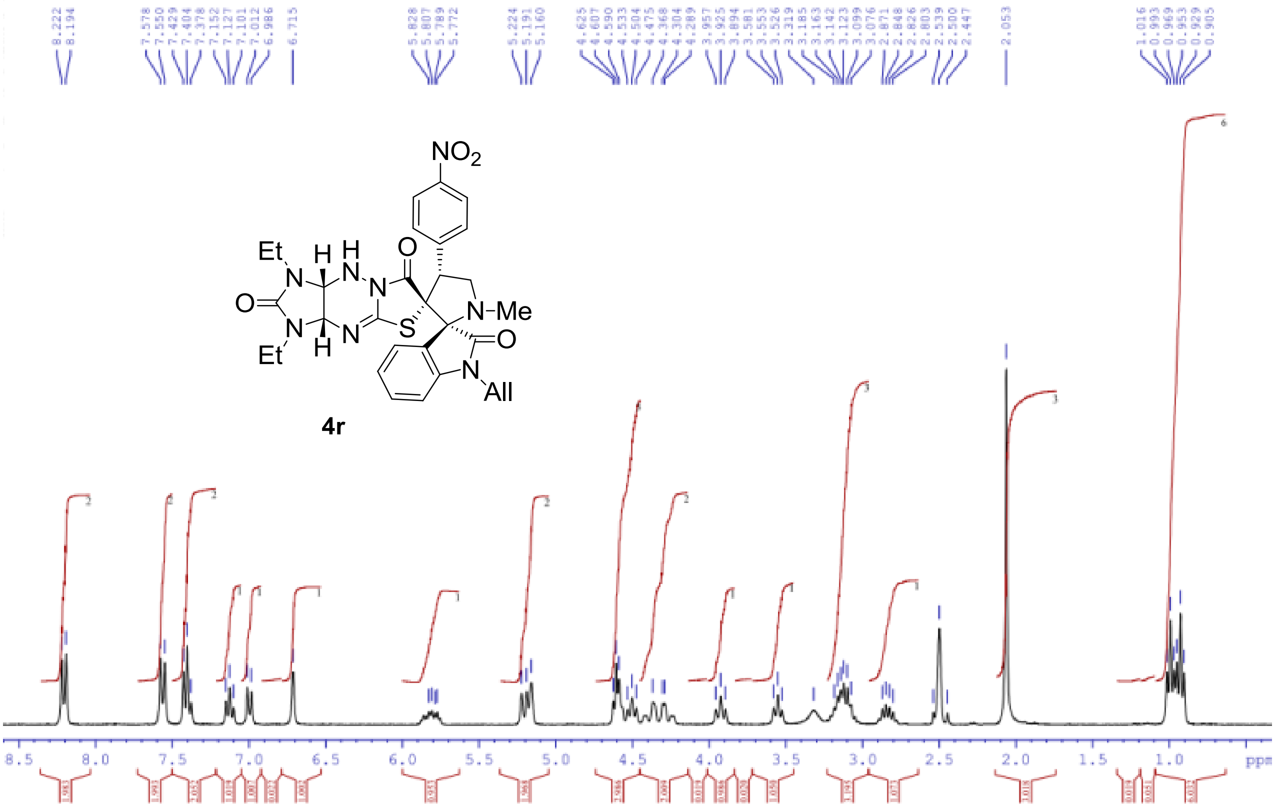

^{1}H NMR spectrum of **4r**

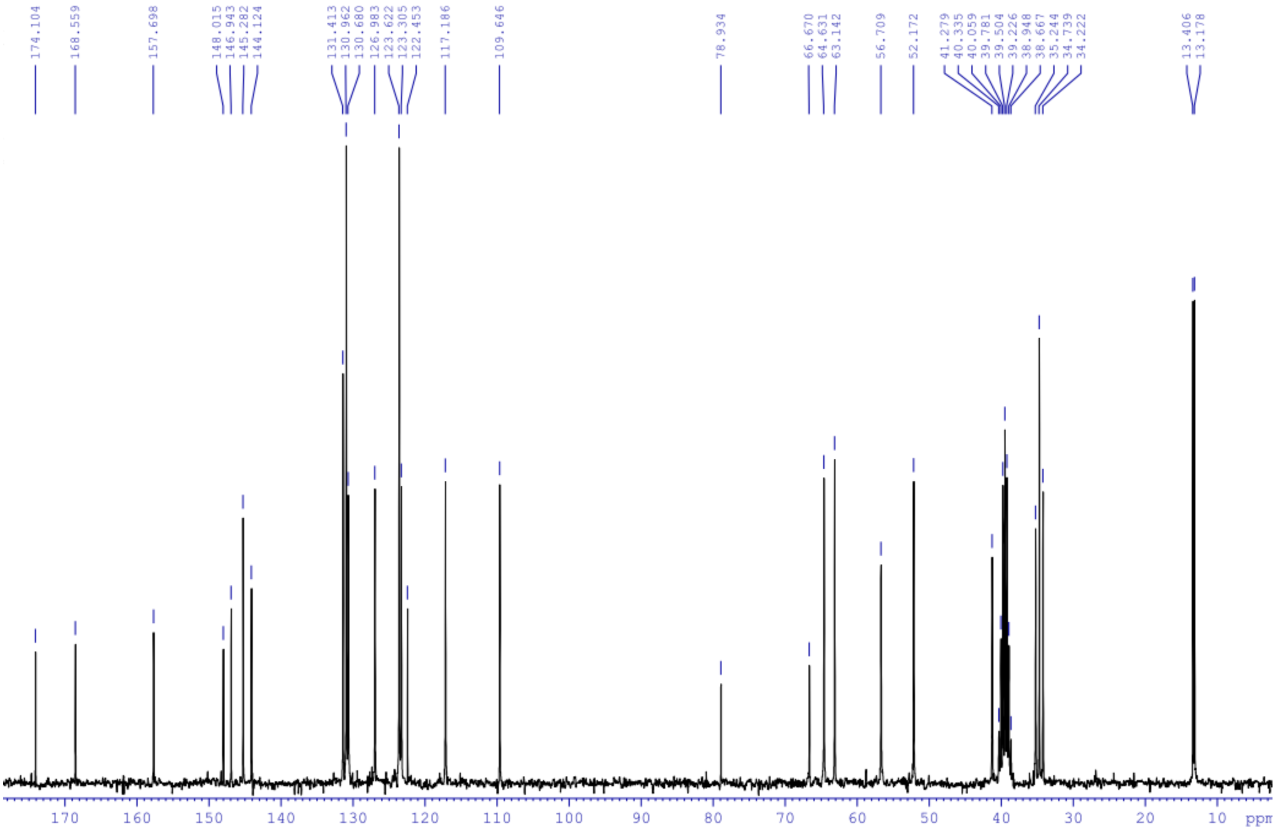

^{1}H NMR spectrum of **4s**

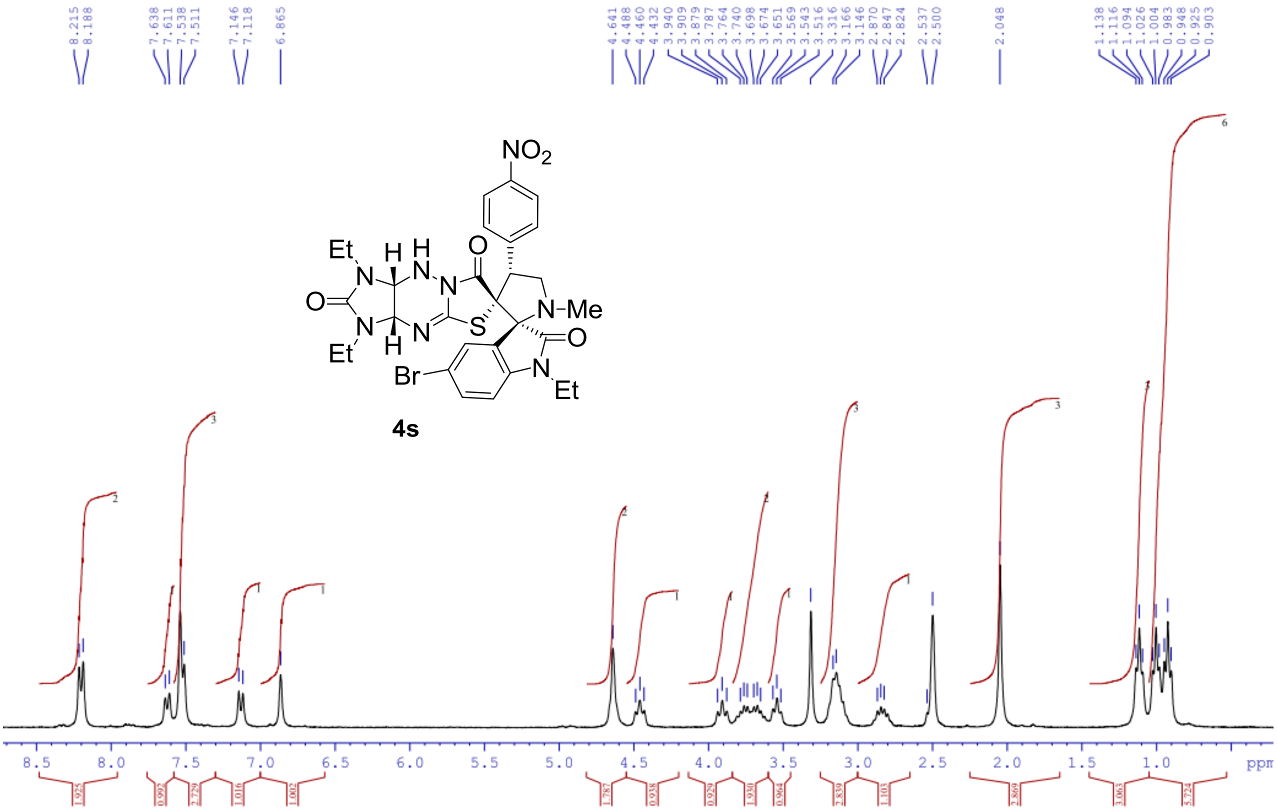

^{13}C NMR spectrum of **4s**

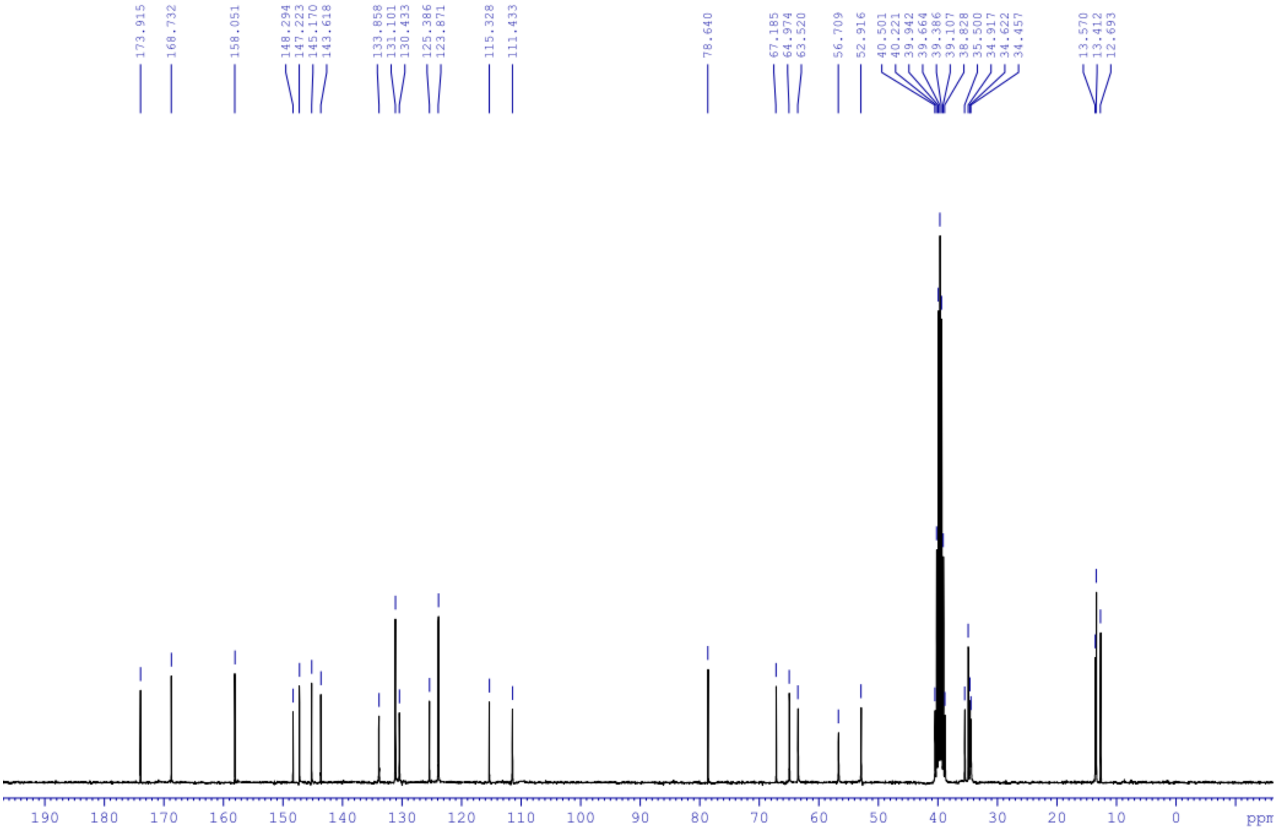

^{1}H NMR spectrum of **4t**

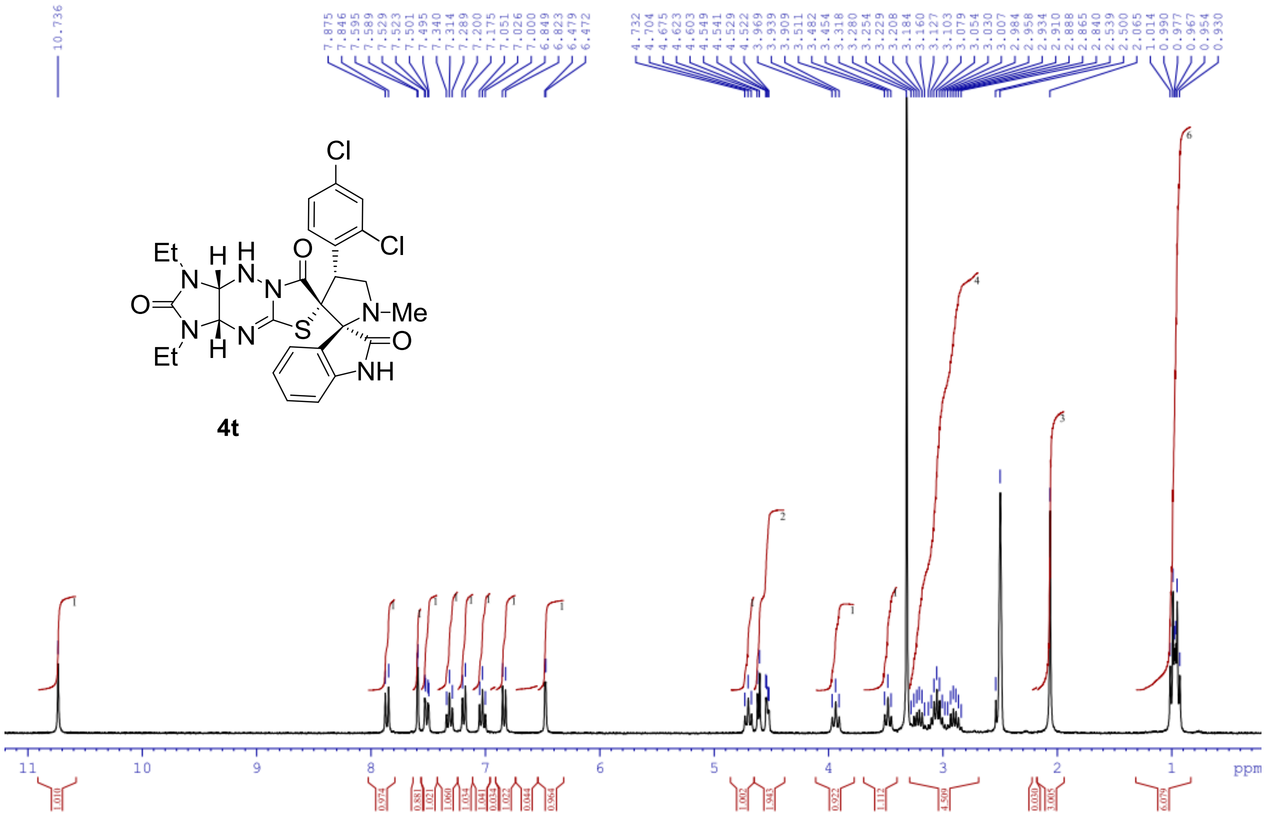

^{13}C NMR spectrum of **4t**

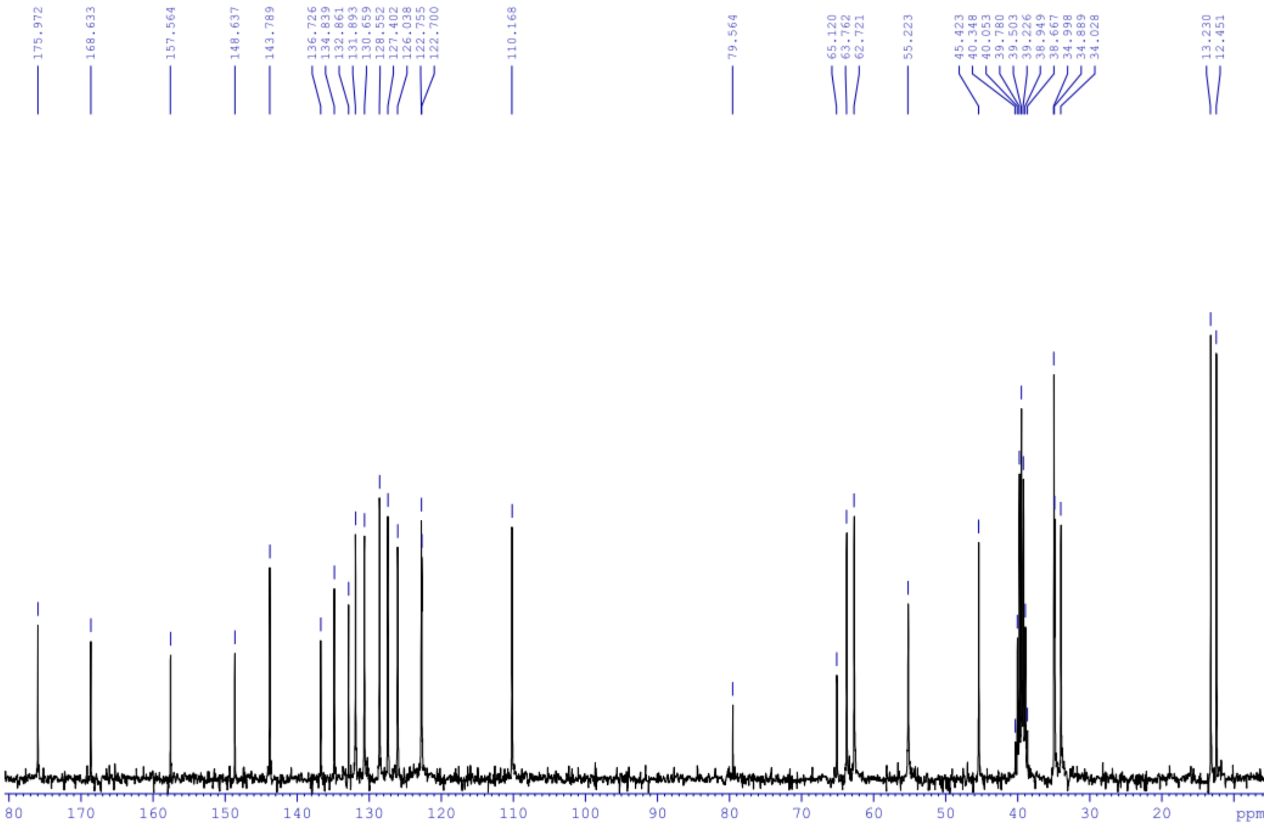

HRMS spectra of compounds **4e**,**g**,**h**,**n**

Display Report

Analysis Info

Analysis Name	D:\Data\Kolotyrkina\2016\Izmest'ev\0126008.d	Acquisition Date	26.01.2016 17:08:05
Method	tune_wide.m	Operator	BDAL@DE
Sample Name	/KANI IA-190	Instrument / Ser#	micrOTOF 10248
Comment	C38H33Br2N7O3S mw 825/827 calibrant added		

Acquisition Parameter

Source Type	ESI	Ion Polarity	Positive	Set Nebulizer	0.4 Bar
Focus	Not active			Set Dry Heater	180 °C
Scan Begin	50 m/z	Set Capillary	4500 V	Set Dry Gas	4.0 l/min
Scan End	3000 m/z	Set End Plate Offset	-500 V	Set Divert Valve	Waste

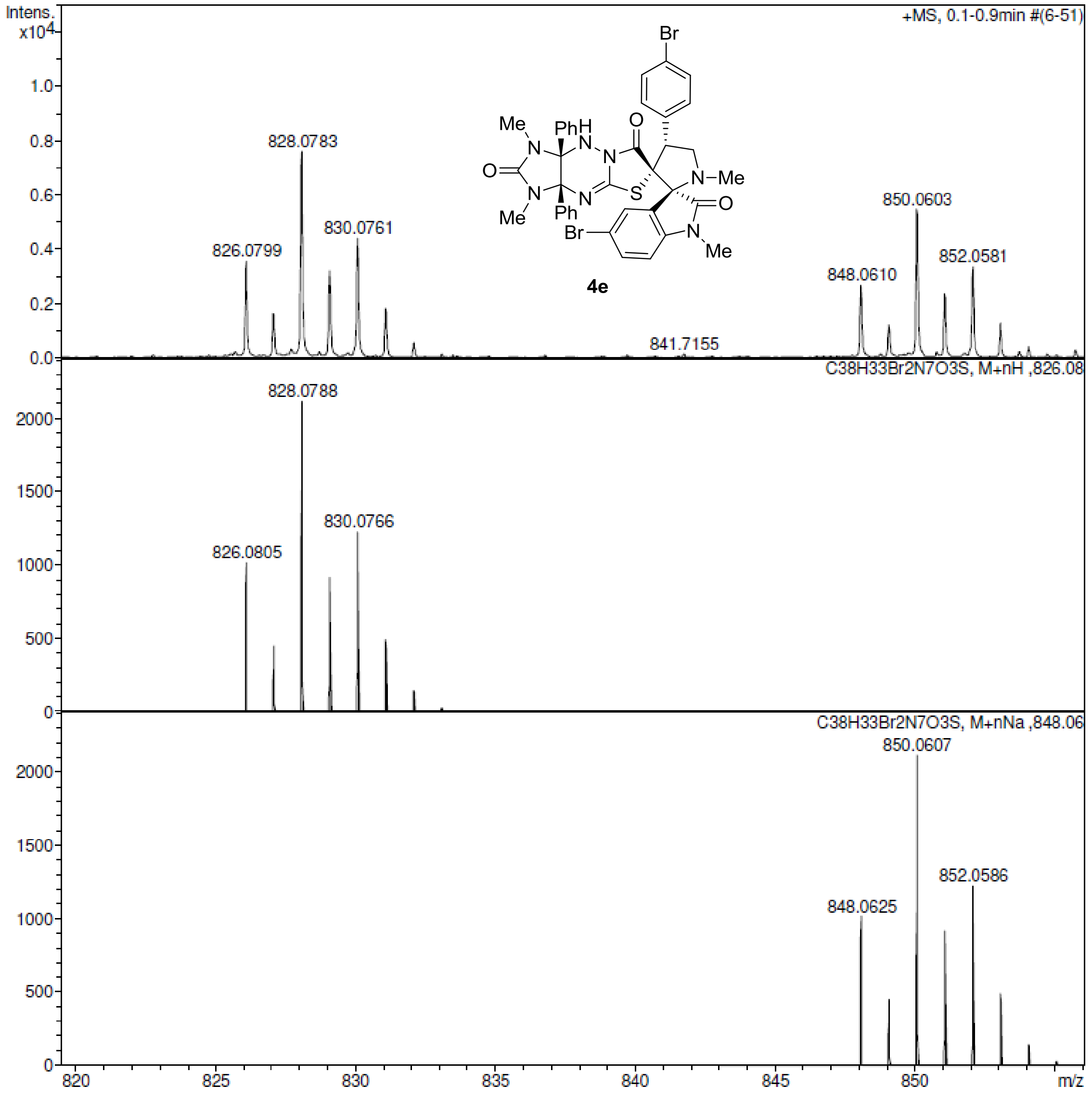

Bruker Compass DataAnalysis 4.0 printed: 26.01.2016 17:11:08 Page 1 of 1

HRMS spectrum of compounds **4e**

Display Report

Analysis Info

Analysis Name	D:\Data\Kolotyrkina\2013\Gazieva\0417013.d	Acquisition Date	17.04.2013 13:54:09
Method	tune_wide.m	Operator	BDAL@DE
Sample Name	/KANI ASY.067	Instrument / Ser#	micrOTOF 10248
Comment	C38H34N8O5S mw 714 calibrant added		

Acquisition Parameter

Source Type	ESI	Ion Polarity	Positive	Set Nebulizer	0.4 Bar
Focus	Not active			Set Dry Heater	180 °C
Scan Begin	50 m/z	Set Capillary	4500 V	Set Dry Gas	4.0 l/min
Scan End	3000 m/z	Set End Plate Offset	-500 V	Set Divert Valve	Waste

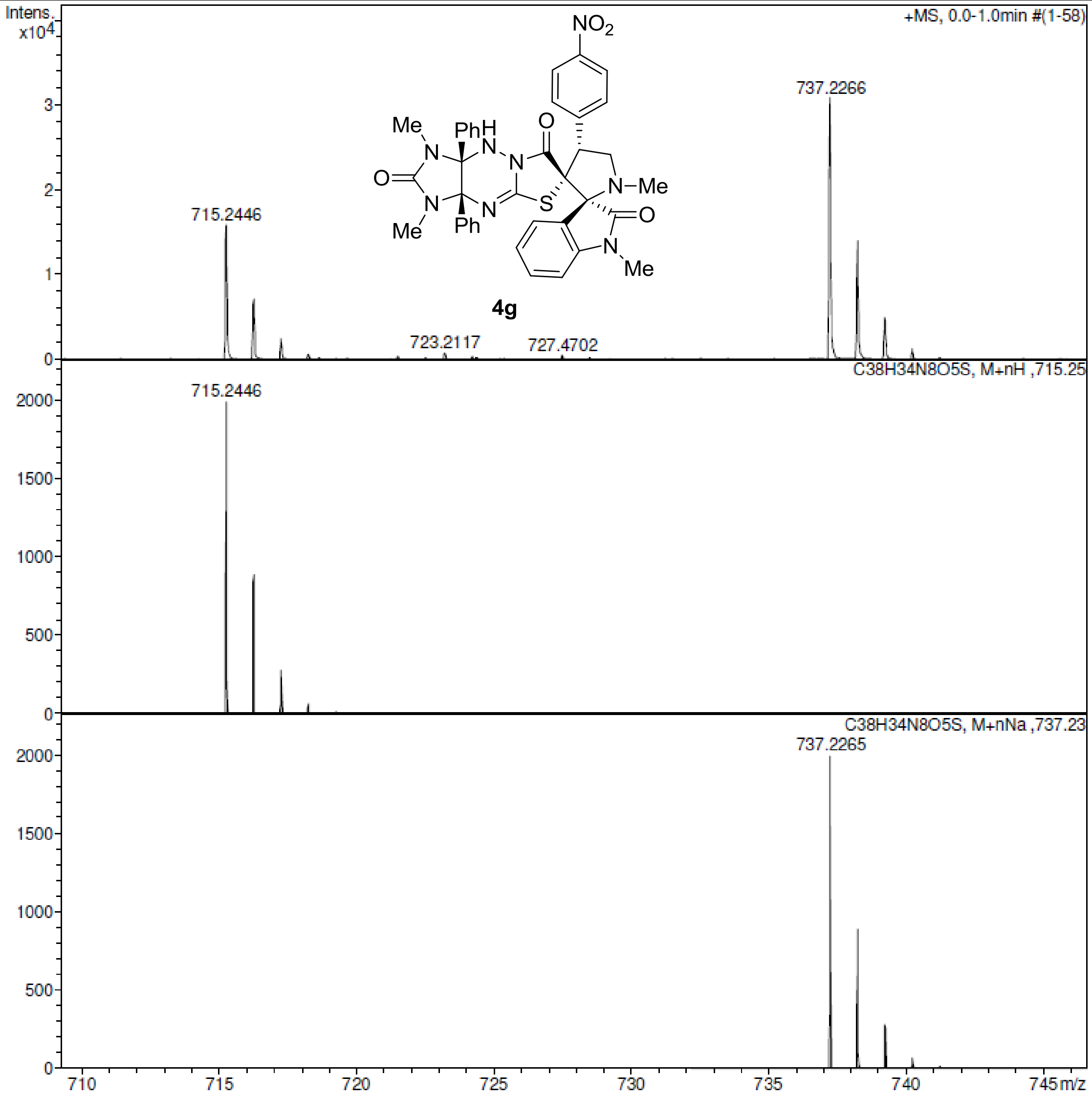

Bruker Compass DataAnalysis 4.0 printed: 17.04.2013 14:57:49 Page 1 of 1

HRMS spectrum of compounds **4g**

Display Report

Analysis Info

Analysis Name	D:\Data\Kolotyrkina\2014\Gazieva\0219001.d	Acquisition Date	19.02.2014 12:07:31
Method	tune_wide.m	Operator	BDAL@DE
Sample Name	/KANI Asy81-1	Instrument / Ser#	micrOTOF 10248
Comment	C39H36N8O5S mw 728 in CH3CN clb added		

Acquisition Parameter

Source Type	ESI	Ion Polarity	Positive	Set Nebulizer	0.4 Bar
Focus	Not active			Set Dry Heater	180 °C
Scan Begin	50 m/z	Set Capillary	4500 V	Set Dry Gas	4.0 l/min
Scan End	3000 m/z	Set End Plate Offset	-500 V	Set Divert Valve	Waste

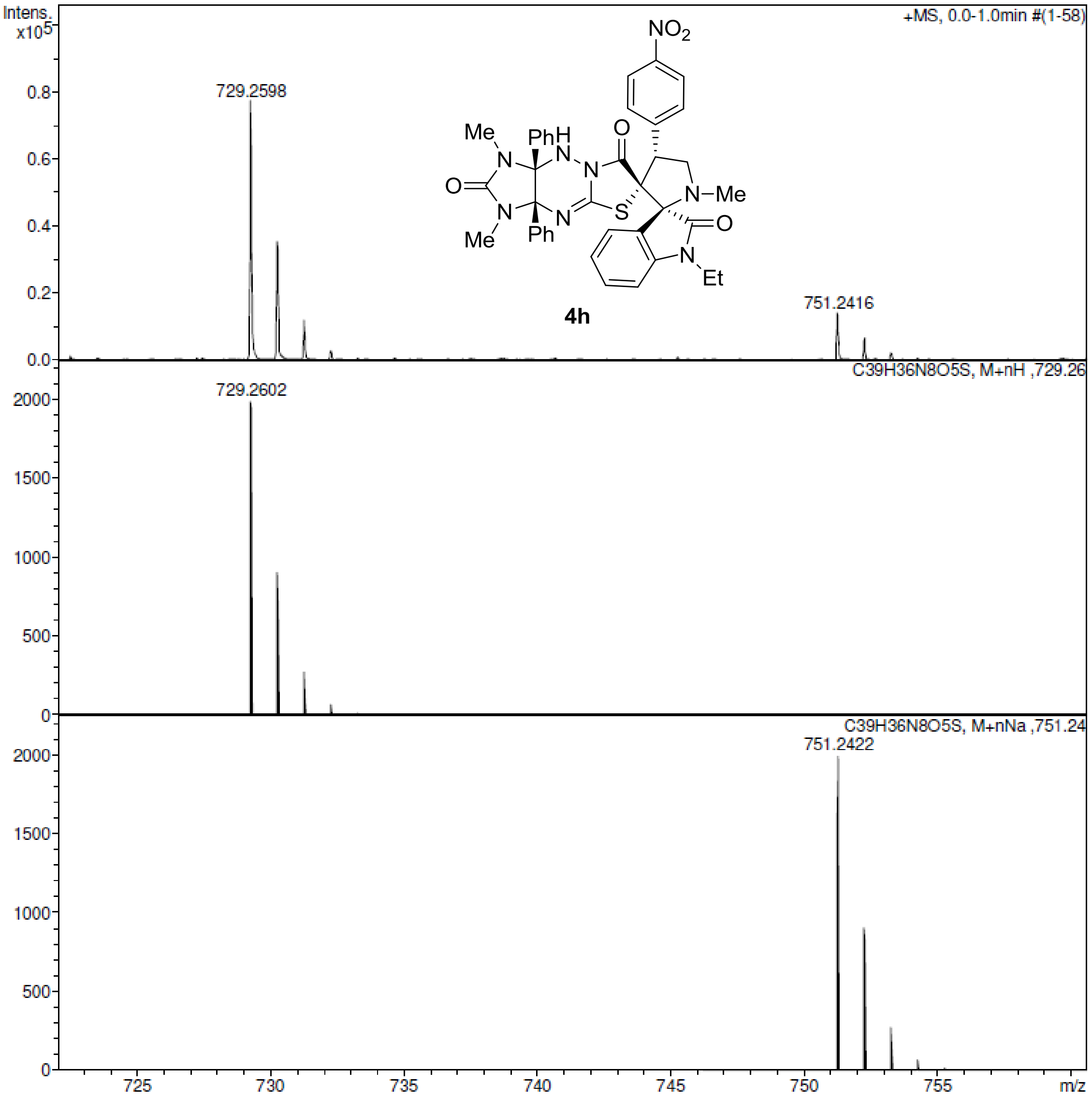

Bruker Compass DataAnalysis 4.0 printed: 19.02.2014 12:10:34 Page 1 of 1

HRMS spectrum of compounds **4h**

Display Report

Analysis Info

Analysis Name	D:\Data\Kolotyrkina\2013\Gazieva\0515002.d	Acquisition Date	15.05.2013 14:03:25
Method	tune_wide.m	Operator	BDAL@DE
Sample Name	/KANI GAZ493.03	Instrument / Ser#	micrOTOF 10248
Comment	C40H37N7O3S mw 695 calibrant added		

Acquisition Parameter

Source Type	ESI	Ion Polarity	Positive	Set Nebulizer	0.4 Bar
Focus	Not active			Set Dry Heater	180 °C
Scan Begin	50 m/z	Set Capillary	4500 V	Set Dry Gas	4.0 l/min
Scan End	3000 m/z	Set End Plate Offset	-500 V	Set Divert Valve	Waste

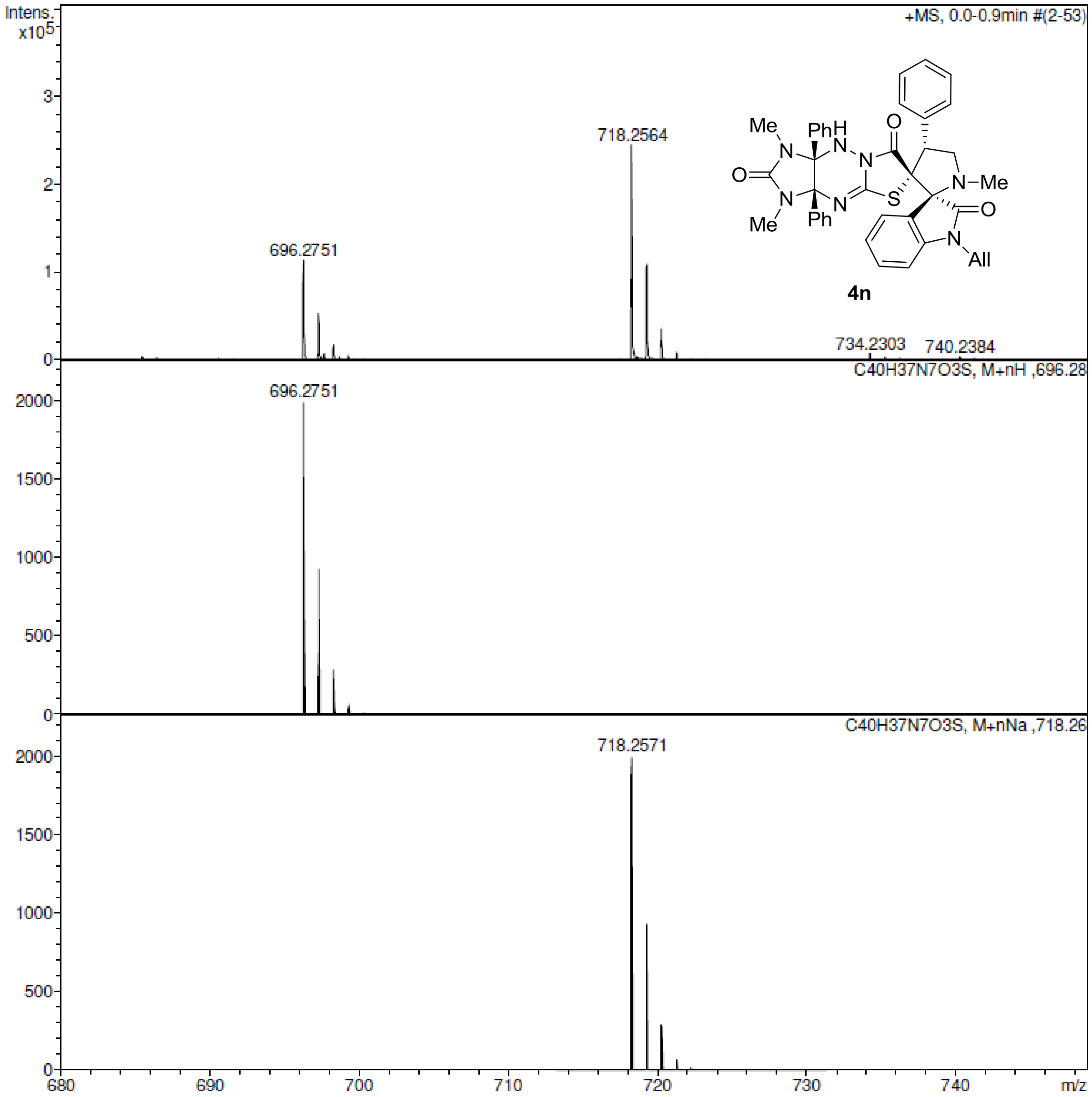

Bruker Compass DataAnalysis 4.0 printed: 15.05.2013 15:07:14 Page 1 of 1

HRMS spectrum of compounds **4n**

Powder diffraction pattern for compounds **4b–d,f–i,l–n**

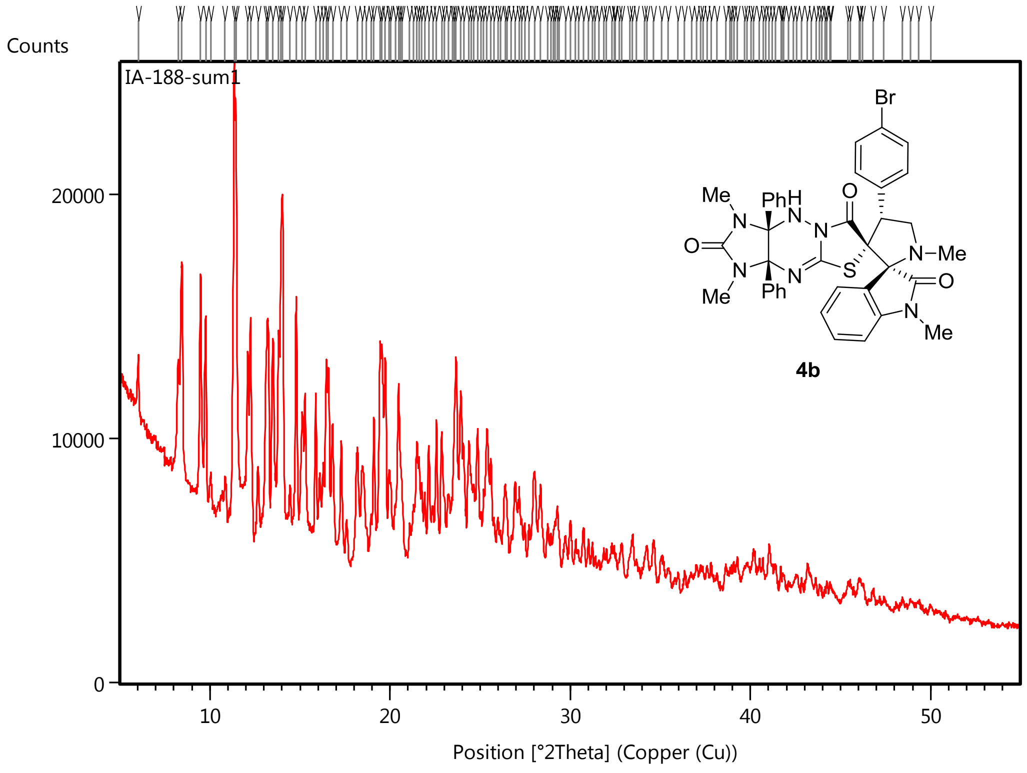

Figure S1: Powder diffraction pattern of **4b** (IA188)

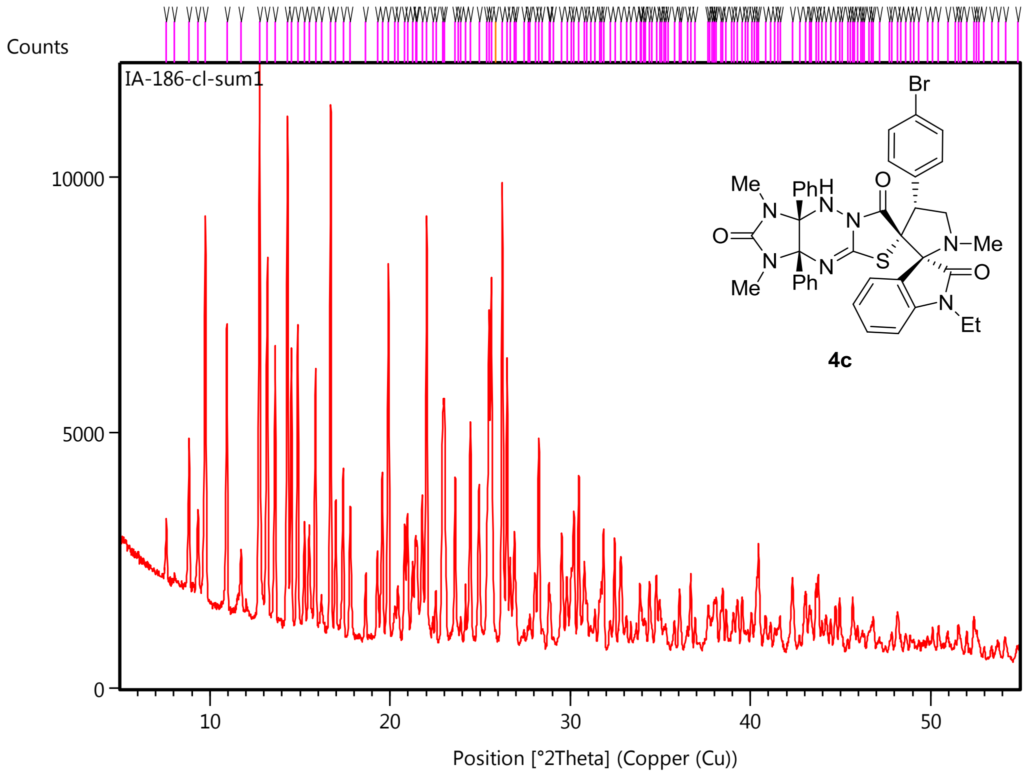

Figure S2: Powder diffraction pattern of **4c** (IA-186)

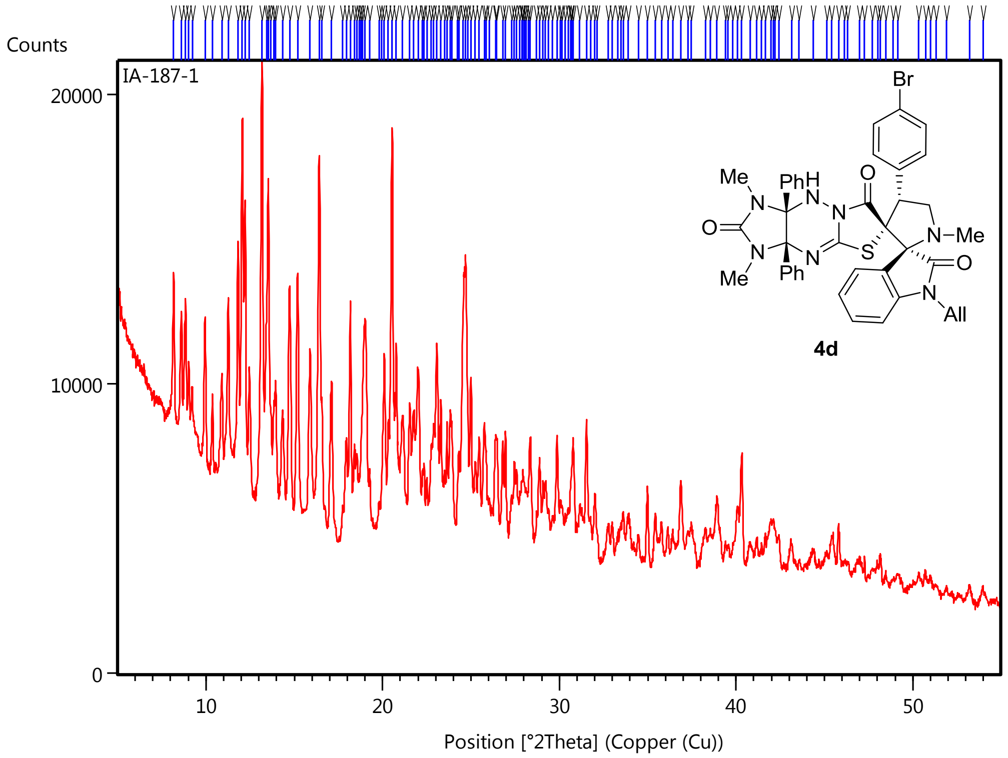

Figure S3. Powder diffraction pattern of **4d** (IA-187-1)

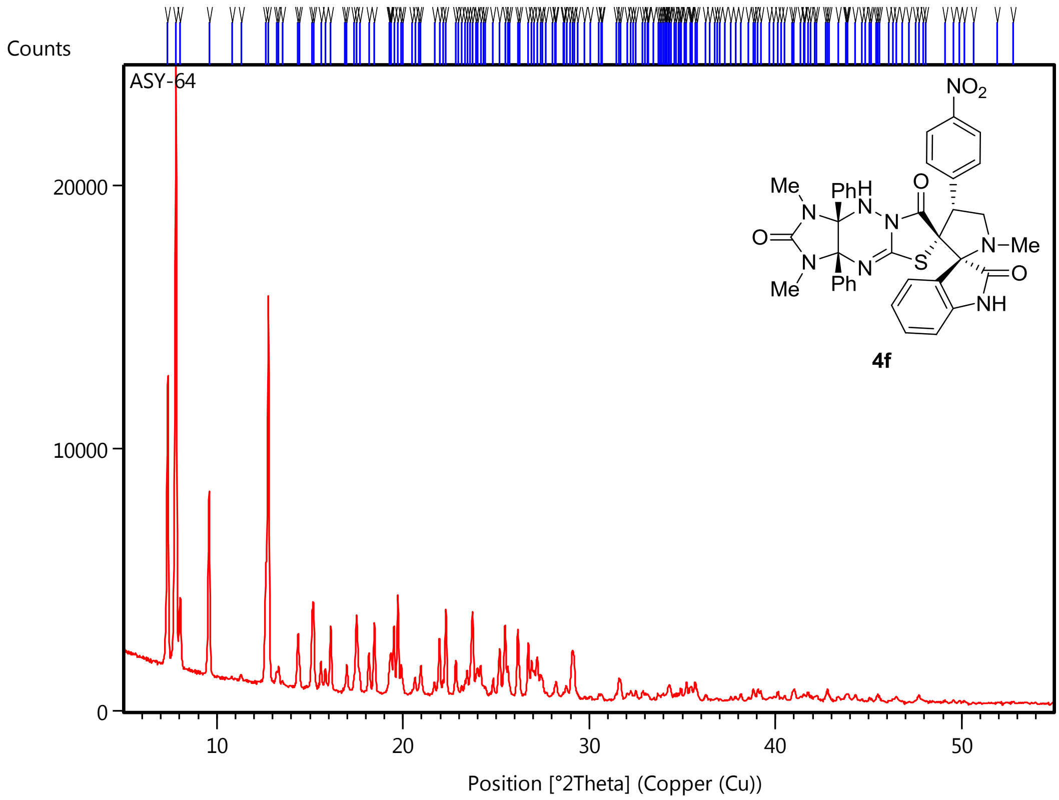

Figure S4: Powder diffraction pattern of **4f** (Asy-64)

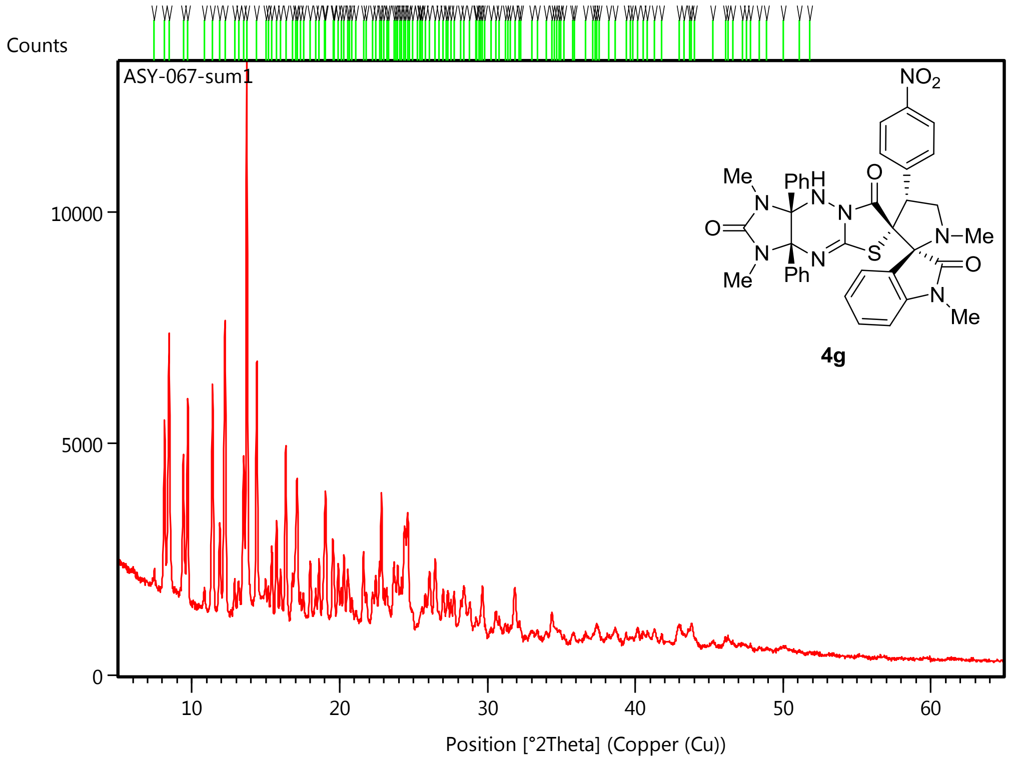

Figure S5: Powder diffraction pattern of **4g** (Asy-067)

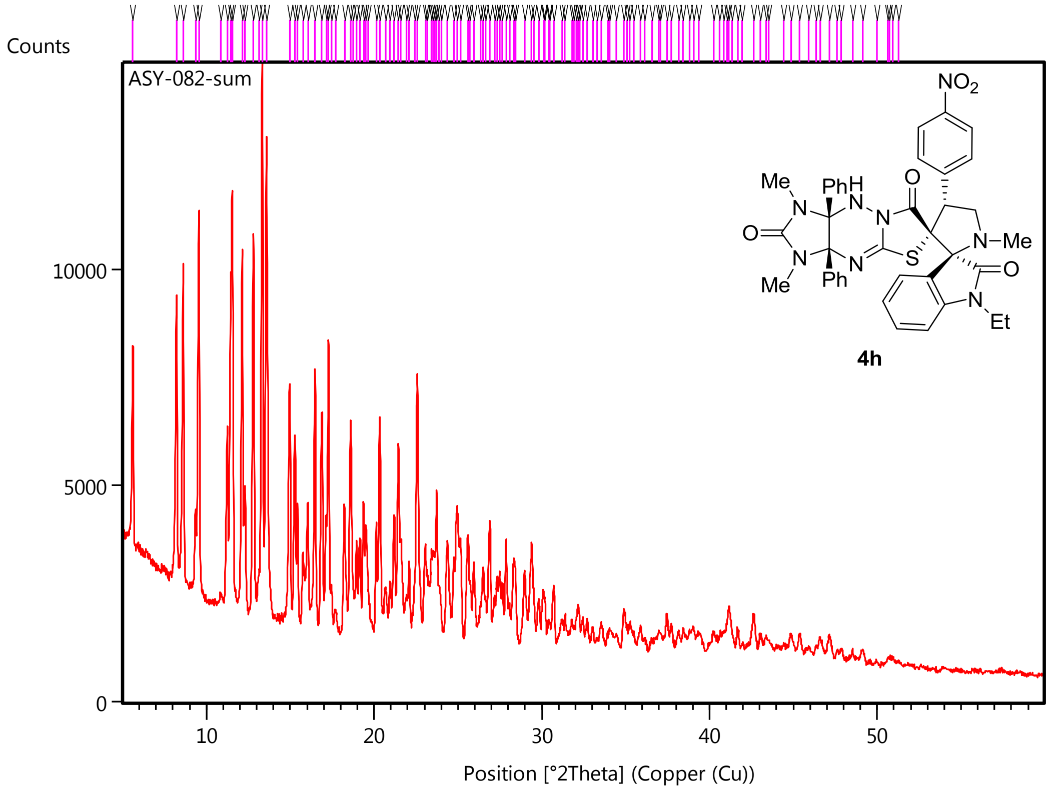

Figure S6: Powder diffraction pattern of **4h** (Asy-082)

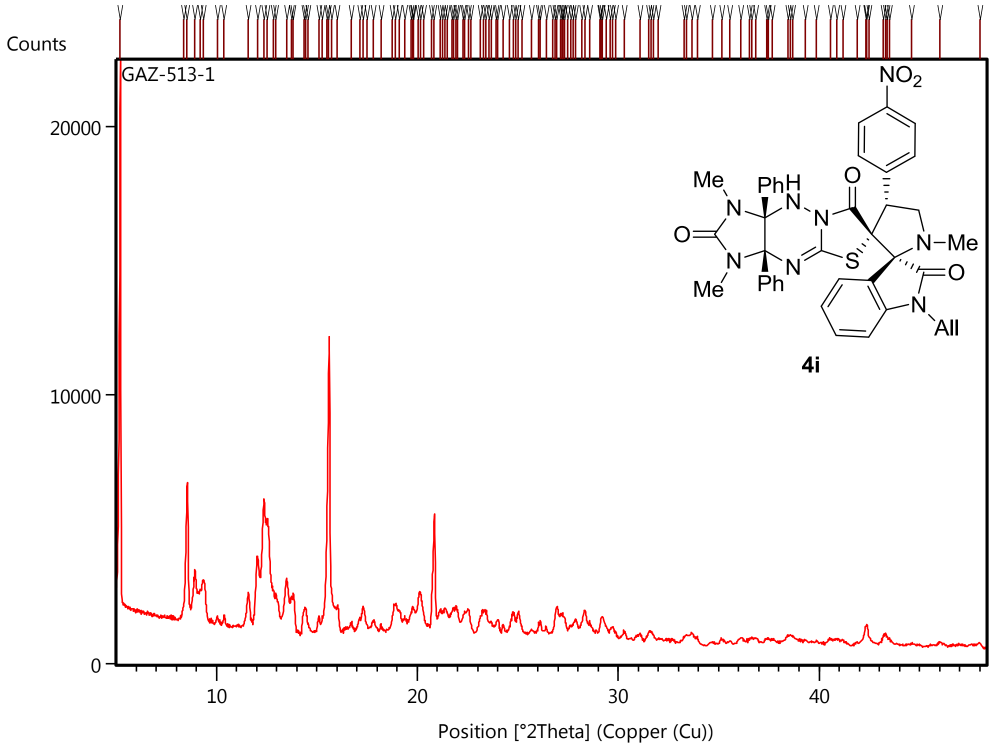

Figure S7: Powder diffraction pattern of **4i** (GAZ-513-1)

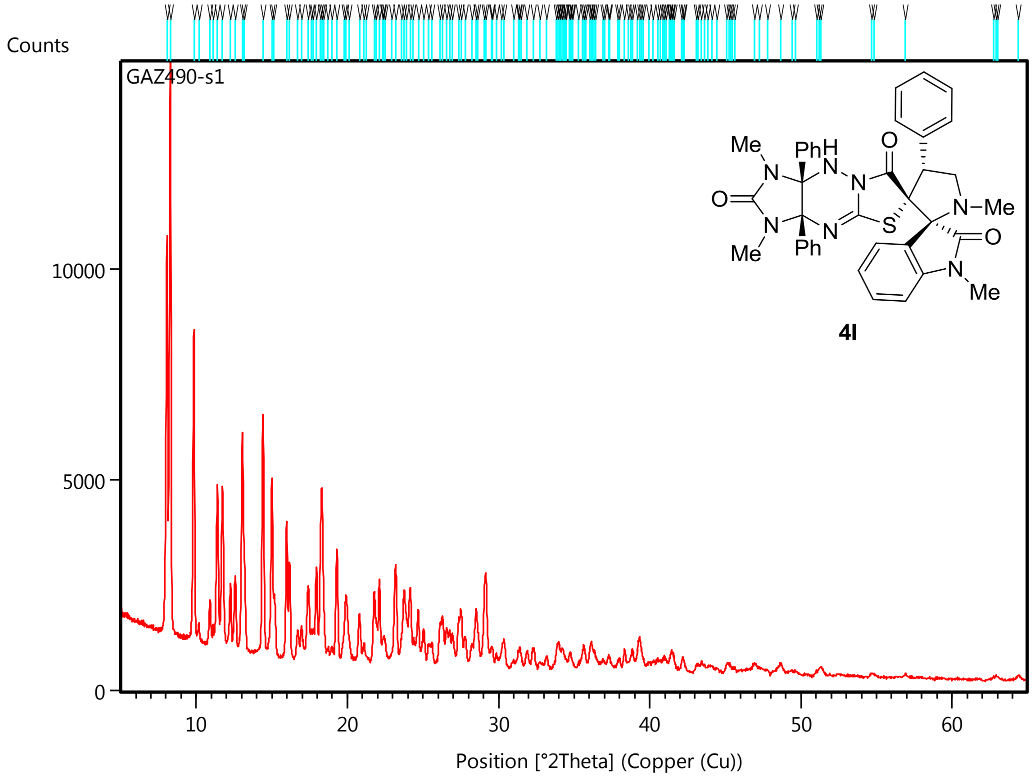

Figure S8: Powder diffraction pattern of **4l** (GAZ-490-1)

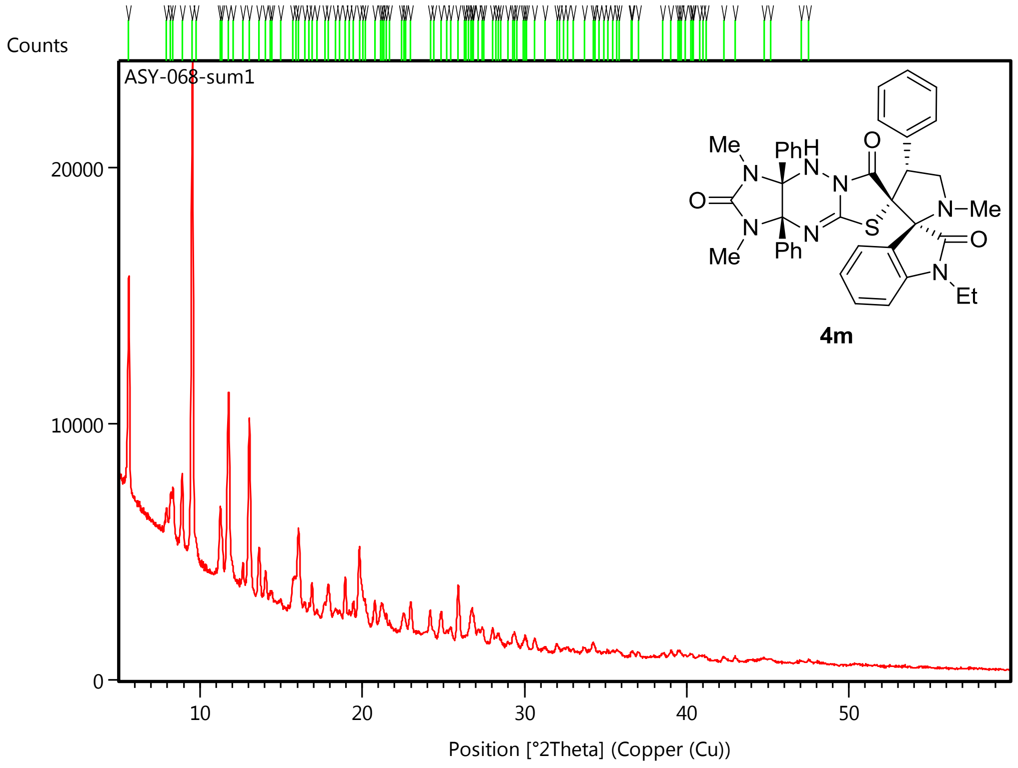

Figure S9: Powder diffraction pattern of **4m** (Asy-068)

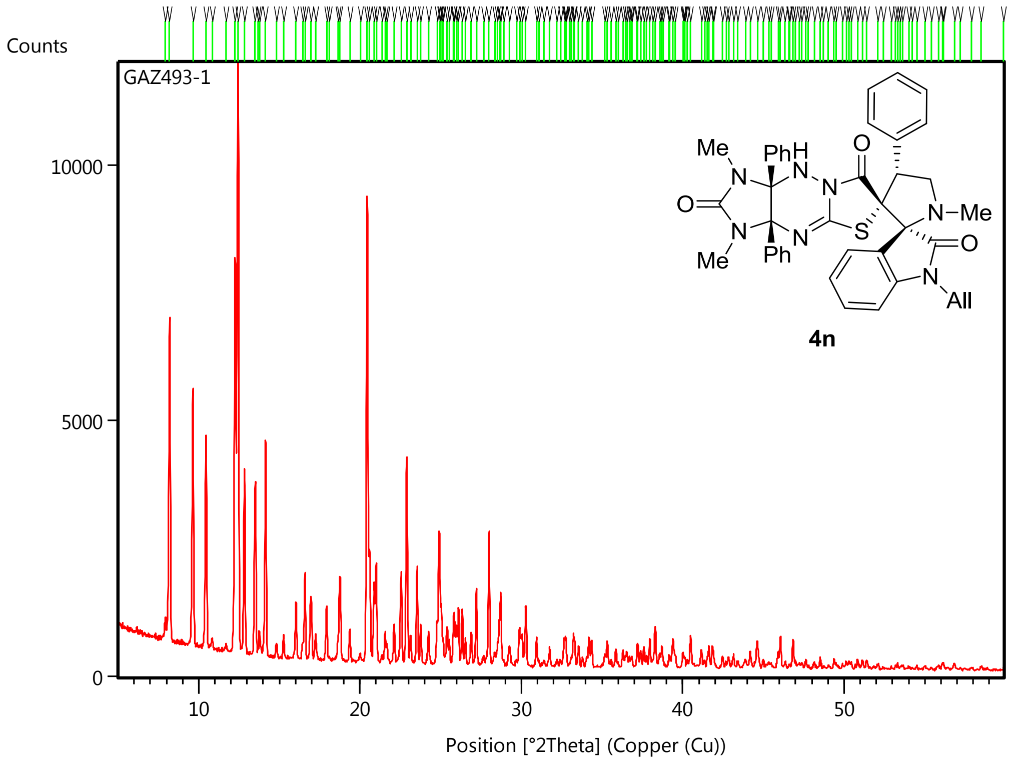

Figure S10: Powder diffraction pattern of **4n** (GAZ493-1)

Table S2. Space groups, unit cell parameters and characteristics of the investigated verification phases of compounds **4b-d,f-i,l-n**

Compound	**4b**	**4c**[a]	**4d**	**4f**	**4g**	**4h**	**4i**	**4l**	**4m**	**4n**
Sp. gr, Z	P-1, Z=4	P-1, Z=4	P-1, Z=4	P-1, Z=2	P-1, Z=4	$P2_1/m$, Z=4	P-1, Z=4	$P\ 1\ 2_1/a\ 1$, Z=4	P-1, Z=4	P-1, Z=4
a, (Å)	17.890(8)	12.597(2)	10.982(1)	11.46(2)	11.152(5)	31.787(3)	16.793(3)	27.152(2)	12.83(3)	14.356(1)
b, (Å)	18.432(5)	17.712(4)	19.811(4)	13.28(3)	13.570(5)	11.435(1)	18.040(3)	11.980(1)	15.94(3)	16.289(2)
c, (Å)	15.718(8)	20.811(5)	19.751(3)	14.01(3)	23.619(6)	10.442(1)	12.843(2)	10.6872(8)	17.88(3)	15.219(2)
α, (°)	108.7(1)	119.4(2)	116.4(1)	61.32(5)	85.40(2)	90	92.74(8)	90	96.2(1)	90.597(6)
β, (°)	69.073(4)	75.985(3)	90.041(2)	73.292(2)	81.263(2)	97.802(2)	92.949(2)	94.144(1)	85.843(3)	96.806(1)
γ, (°)	133.072(4)	113.666(3)	80.919(2)	79.744(2)	79.570(2)	90	108.572(3)	90	82.709(3)	90.366(1)
V(Å^3)	3514.00	3698.69	3787.30	1791.17	3453.22	3760.34	3674.80	3467.14	3592.61	3533.58
No. Indexed Lines	163	172	155	174	132	140	133	156	108	180
Total No. Calculated Lines	14789	14788	14905	7837	13041	6105	15755	6113	15487	15477
Snyder's FOM	13.4966	10.5108	12.1894	26.2624	10.9476	22.9330	13.1462	16.8326	10.9729	11.2313

[a]Unit cell parameters of **4c** are different from those obtained by single crystal X-ray analysis due to the absence of solvent molecules in the investigated powder sample.
